# Supplementary material for: An exploratory open-label multicentre phase I/II trial evaluating the safety and efficacy of postnatal or prenatal and postnatal administration of allogeneic expanded fetal mesenchymal stem cells for the treatment of severe osteogenesis imperfecta in infants and fetuses: the BOOSTB4 trial protocol
Source: BMJ Open. 2024 Jun 4;14(6):e079767. doi: 10.1136/bmjopen-2023-079767 (PMC11163617; doi:10.1136/bmjopen-2023-079767)
Supplement: Supplementary data [file bmjopen-2023-079767supp001.pdf]

CLINICAL TRIAL SYNOPSIS

Boost Brittle Bones Before Birth

TRIAL REGISTRATION DATA SET	
**Data category**	**Information**
Registry trial identifying number	EudraCT no: 2015-003699-60, ClinicalTrials.gov ID: NCT03706482
Secondary identifying numbers	KIBB01
Sources of monetary support	Academic: European Union's Horizon 2020 research and innovation programme under grant agreement No 681045, the Swedish Research Council, Region Stockholm in Sweden
Sponsor	Karolinska Institutet
Contact for queries	boostb4@clintec.ki.se
Title	An exploratory, open label, multiple dose, multicentre phase I/II trial evaluating safety and efficacy of postnatal or prenatal and postnatal intravenous administration of allogeneic expanded fetal mesenchymal stem cells for the treatment of severe Osteogenesis Imperfecta compared with a combination of historical and untreated prospective controls.
Trial code	BOOSTB4
Countries of recruitment	Sweden, Netherlands, United Kingdom
Health condition studied	Osteogenesis Imperfecta type 3 and severe type 4
Intervention	Intravenous administration of BOOST cells
Investigational Medicinal Product	BOOST cells (expanded human 1st trimester fetal liver-derived mesenchymal stem cells)
Study type	Interventional
	Open label
	Primary purpose: Treatment of chronic condition
	Phase I/II
Trial groups	Postnatal group (4 postnatal doses), n=15
	Prenatal group (1 prenatal dose & 3 postnatal doses), n=3
	Prospective control group, n=up to 30
	Historical control group, n=1–5 per subject
Date of first enrolment	March 2020
Target sample size	Initially n=15 in the postnatal group and n=15 in the prenatal group, later amended to n=15 in the postnatal group and n=3 in the prenatal group
Recruitment status	Completed
Primary outcomes	Safety and tolerability
Key secondary outcomes	Efficacy: fracture incidence, growth, bone mineral density

Sagar RL, *et al. BMJ Open* 2024; 14:e079767. doi: 10.1136/bmjopen-2023-079767

Protocol Summary

PROTOCOL IDENTITY

Trial Title: Boost Brittle Bones Before Birth (BOOSTB4)

An exploratory, open label, multiple dose, multicentre phase I/II trial evaluating safety and efficacy of postnatal or prenatal and postnatal intravenous administration of allogeneic expanded fetal mesenchymal stem cells for the treatment of severe Osteogenesis Imperfecta compared with a combination of historical and untreated prospective controls.

EudraCT number	Protocol Number	Trial Code	Phase of Development
2015-003699-60	KIBB01	BOOSTB4	I/II

Sponsor: Karolinska Institutet, Stockholm, Sweden

Sponsor's representative: Associate Professor Cecilia Götherström, Karolinska Institutet, Department of Clinical Science, Technology and Intervention, Division of Obstetrics and Gynaecology, Stockholm, Sweden

Principal Coordinating Investigator: Dr Eva Åström, Pediatric Neurology and Musculoskeletal disorders and Home care, Astrid Lindgren Children's Hospital at Karolinska University Hospital, Stockholm, Sweden

Principal Investigator: Dr Eva Åström, Pediatric Neurology and Musculoskeletal disorders and Home care, Astrid Lindgren Children's Hospital at Karolinska University Hospital, Stockholm, Sweden

Trial centres: Karolinska University Hospital, Sweden[1]; University College London Hospitals NHS Foundation Trust[2] and Great Ormond Street Hospital for Children NHS Foundation Trust[2], the United Kingdom; University Hospital Cologne[2], Germany and Leiden University Medical Centre[3] and University Medical Centre Utrecht[3], the Netherlands.

[1]Performing the active part of the trial, i.e. dose administration and immediate, primary and long-time follow-up, and inclusion of historical and prospective controls.

[2]Performing inclusion of historical controls.

[3]Performing inclusion of historical and prospective controls and primary and long-time follow-up of two Dutch subjects.

INVESTIGATIONAL MEDICINAL PRODUCT (IMP)

Test Product: BOOST cells (allogeneic expanded human 1st trimester liver-derived mesenchymal stem cells [MSC])

Pharmaceutical Form: Cell suspension

Dose: $3x10^6$ viable BOOST cells/kg body weight

Route of Administration: Intravenous administration postnatally or prenatally

Manufacturer: Karolinska Cell Therapy Centre, Vecura at Karolinska University Hospital in Sweden

Sagar RL, *et al. BMJ Open* 2024; 14:e079767. doi: 10.1136/bmjopen-2023-079767

BOOSTB4

TRIAL DESIGN

Hypothesis: Early administration of MSC will ameliorate severe types of OI.

Trial design:
This will be a multi-centre, open label, multiple-dose, phase I/II trial of allogeneic expanded human first trimester fetal MSC administration for the treatment of Osteogenesis Imperfecta (OI) type 3 or severe type 4 compared with historical and untreated prospective controls.

The trial is divided into 2 parts where Part 1 includes administration of 4 postnatal doses per subject and Part 2 includes administration of one prenatal and 3 postnatal doses per subject:

Part 1: Postnatal administration (n=15 in total)

- Administration of four postnatal doses of MSC to 15 subjects
- A safety assessment is performed by the Data Safety Monitoring Board (DSMB) of the first MSC administration to the first 5 subjects[1] in any participating country treated postnatally.

[1]The 5 subjects included and administered with 1 MSC dose postnatally in Part 1 of the trial will receive the additional 3 MSC doses as planned, i.e. at +4, +8 and +12 months after the first MSC dose. Available data from such administration will be included in the data set sent to the DSMB for the safety assessment. There will be no planned pause in the postnatal administration during the safety assessment by the DSMB for opening of Part 2 of the trial. The Stopping rules on subject level apply to all steps (see Section 10.9).

Part 2: Prenatal administration (n=3 in total)

- One prenatal MSC dose to 3 subjects[2].

[2]The 3 subjects included and administered with 1 MSC dose prenatally in Part 2 of the trial will receive the additional 3 postnatal MSC doses as planned, i.e. at +4, +8 and +12 months after the prenatal dose. The Stopping rules on subject level apply to all steps (see Section 10.9).

Duration:
The trial is divided into two periods where Period 1 includes the administration of MSC and immediate and primary follow-up, and Period 2 that includes the long-time follow-up (see the Trial schedule below):

Period 1: Four doses of MSC every 4 months with immediate follow-up after each MSC dose, and primary follow-up at 6 and 12 months after the fourth and last MSC dose

Period 2: Yearly long-time follow-up until 10 years after the first MSC dose

Subjects in the postnatal group will be included, receive the first dose and evaluated as soon as possible after birth up to 18 months of corrected age. They will receive 3 additional doses at +4, +8 and +12 months (±1 month) after the 1st dose.

Subjects in the prenatal group will be included in the trial and receive the 1st dose prenatally (between 16+0 – 35+6 weeks+days of gestation) and receive 3 additional postnatal doses at +4 (or as soon as possible after birth), +8 and +12 months (±1 month) after the 1st dose.

Subjects can be enrolled during the initial 3 years of the trial. The expected duration of treatment is 12 months, the minimum treatment time is 9 months and the maximum treatment time is 15 months.

Sagar RL, *et al. BMJ Open* 2024; 14:e079767. doi: 10.1136/bmjopen-2023-079767

BOOSTB4

Trial Schedules in Period 1 (GW=gestation weeks in weeks+days. All postnatal time points are ± 1 month)**:**

Postnatal group

Diagnosis	Dose 1 & immediate follow-up	Dose 2 & immediate follow-up	Dose 3 & immediate follow-up	Dose 4 & immediate follow-up	Primary follow-up	Primary follow-up
Clinical & molecular diagnosis of type 3 / severe type 4 OI	As soon as possible after birth up to 18 months of age	+4 months after dose 1	+4 months after dose 2	+4 months after dose 3	+6 months after dose 4	+12 month after dose 4

Prenatal group

Diagnosis	Dose 1 & immediate follow-up	Post prenatal dose follow-up	Birth	Dose 2 & immediate follow-up	Dose 3 & immediate follow-up	Dose 4 & immediate follow-up	Primary follow-up	Primary follow-up
Clinical & molecular diagnosis of type 3 / severe type 4 OI	Gestation weeks 16+0 – 35+6	Every 2 weeks up to birth		+4 months after dose 1	+4 months after dose 2	+4 months after dose 3	+6 months after dose 4	+12 months after dose 4

TRIAL OBJECTIVES

Primary objectives:

To assess safety and tolerability in the child, fetus and woman after postnatal or prenatal and postnatal intravenous administration of 4 doses of BOOST cells every 4 months in subjects with OI type 3 or severe type 4.

Secondary objectives:

To assess the effect of intravenous administration of 4 doses of BOOST cells every 4 months in subjects with OI type 3 or severe type 4 on:

1. Number of fractures from baseline to primary and long-time follow-up
2. Time (days) to first fracture after last dose
3. Number of fractures at birth (prenatal treatment group only, and postnatal treatment group when available)
4. Bone mineral density (BMD)
5. Growth
6. Clinical status of OI
7. Biochemical bone turnover

Exploratory objectives

1. To study the impact of 4 doses of fetal MSC on Quality of Life (QoL)
2. To study the extent of donor cell engraftment in tissue samples
3. To study paracrine effects of 4 doses of fetal MSC
4. To study the effect of 4 doses of fetal MSC on endogenous immune cells
5. To study non-invasive prenatal diagnosis of OI (will not be used in the diagnostic procedure in the trial)

TRIAL ENDPOINTS

Primary Endpoints:

The primary endpoint is safety and tolerability measured as seriousness, severity and frequency of MSC administration related adverse events (AEs), with specific focus on the following:

Sagar RL, *et al. BMJ Open* 2024; 14:e079767. doi: 10.1136/bmjopen-2023-079767

1. Vital signs in conjunction with the MSC administration
2. Transfusion reactions (administration toxicity, allergy, embolism)
3. Immune reaction with or without symptoms of inflammation, potentially resulting in rejection of the cells or development of donor-specific antibodies:
   - Allergy or Hypersensitivity responses to antibiotics or antimycotics
   - Development of Fetal Bovine Serum-specific antibodies
   - Hypersensitivity responses to Human Serum Albumin
   - Hypersensitivity to impurities in the IMP
4. Prenatal complications (miscarriage/intrauterine fetal death, premature birth, infection *in utero* or persistent [>1 min] fetal bradycardia) in the prenatal group
5. Adverse effects of feto-maternal transmission of donor cells in the prenatal group
6. Tumourigenicity
7. Mortality/morbidity

Key Secondary Endpoint:

1. Number of fractures from baseline to primary and long-time follow-up

Secondary Endpoints:

1. Time (days) to first fracture after last dose
2. Number of fractures at birth (prenatal group, and postnatal group when available)
3. Change in BMD (g/cm^2)
4. Growth (cm and kg)
5. Change in clinical status of OI based on parameters defined under efficacy assessments described below
6. Change in biochemical bone turnover

Exploratory endpoints:

1. The QoL will be assessed through the Infant Toddler Quality of Life Questionnaire™ (ITQOL)
2. Tissue samples (bone, bone marrow, muscle and skin, placenta, amniotic fluid, umbilical cord, umbilical cord blood, peripheral blood) will be collected in conjunction with clinically indicated surgery and at birth (prenatal group only) and analysed for donor cell engraftment
3. Paracrine effects will be analysed from plasma isolated from peripheral blood
4. Effect on immune cells will be analysed from peripheral blood
5. Non-invasive prenatal diagnosis will be studied during the trial

POPULATION OF TRIAL SUBJECTS

Description of Trial Subjects:

1. Infants diagnosed with OI type 3 or severe type 4 on clinical grounds with a confirmatory sequence variant (mutation) in one or other of the type I collagen genes (*COL1A1* and *COL1A2*) (n=15)
2. Pregnant women whose fetus has been diagnosed with OI type 3 or severe type 4 prenatally on ultrasound parameters with a confirmatory sequence variant (mutation) in one or other of the type I collagen genes (*COL1A1* and *COL1A2*) (n=3)
3. Controls diagnosed with OI type 3 or severe type 4 on clinical grounds with a confirmatory sequence variant (mutation) in one or other of the type I collagen genes (*COL1A1* and *COL1A2*):
   a) Matched historical controls (n=30-150) will be identified in national registries
   b) Prospective untreated controls (n=up to 30). Subjects eligible for the trial but not willing/able to participate.

Sagar RL, *et al. BMJ Open* 2024; 14:e079767. doi: 10.1136/bmjopen-2023-079767

Only patients diagnosed with qualitative (Glycine) substitution in the collagen triple-helix encoding region of either the *COL1A1* or *COL1A2* gene will be included in the trial. The diagnosis will be made both clinically and with molecular analysis by a multidisciplinary team.

No formal power calculations have been performed. OI is a rare disease and a limited number of subjects can be included each year. Based on the incidence, it is reasonable to include 30 patients in this first trial on the grounds of feasibility, suitable time period and for the application of descriptive statistical analyses.

INCLUSION CRITERIA

For inclusion, all criteria should apply.

Postnatal group

1. Parent's/legal guardian's signed informed-consent form
2. Clinical diagnosis of OI type 3 or 4 AND
3. Molecular diagnosis of OI (Glycine substitution in the collagen triple-helix encoding region of either the *COL1A1* or *COL1A2* gene)
4. Age less than 18 months (calculated from gestational week 40+0, i.e. the corrected age)
5. Parent/legal guardian over 18 years of age

Prenatal group:

1. Woman has signed the patient consent form
2. Only women where termination of the pregnancy is no longer possible or where the women are committed to continue the pregnancy may be included in the trial
3. Suspicion of OI type 3 or 4 in the fetus on ultrasound findings AND
4. Molecular diagnosis of OI in the fetus (Glycine substitution in the collagen triple-helix encoding region of either the *COL1A1* or *COL1A2* gene)
5. Gestation age between 16+0 and 35+6 weeks+days
6. Pregnant women over 18 years of age

After birth and before the 2nd dose, subjects in the prenatal group will be assessed for the inclusion criteria for the postnatal group.

Control group:

a) Matched historical controls

1. Parent's/legal guardian's signed informed-consent form
2. Clinical and molecular diagnosis of OI (Glycine substitution in the collagen triple-helix encoding region of either the *COL1A1* or *COL1A2* gene)
3. Data on fractures and growth is available
4. Parent/legal guardian over 18 years of age

b) Prospective untreated controls

Postnatal participation: The inclusion criteria for the postnatal group apply

Prenatal participation: The inclusion criteria for the prenatal group apply, except inclusion criteria 2.

Sagar RL, *et al. BMJ Open* 2024; 14:e079767. doi: 10.1136/bmjopen-2023-079767

EXCLUSION CRITERIA

For exclusion, one is enough to exclude.

Postnatal group:

1. Existence of other known disorder that might interfere with the treatment, such as, but not limited to organ disfunction (for example liver or renal failure or bronchopulmonary dysplasia), congenital heart defect, hypoxic encephalopathy I-III, severe neurological problems, immune deficiencies, muscle diseases, severe malformations or syndromes diagnosed by clinical examination
2. Any contraindication for invasive procedures such as a moderate/severe bleeding tendency
3. Known risk factors for clotting, such as, but not limited to previous blood clot, family history of clots, clotting disorder (inherited or acquired), heart failure, inflammatory disorders (for example lupus, rheumatoid arthritis, inflammatory bowel disease)
4. Positive Donor Specific Antibody-test
5. Known allergy/hypersensitivity to Fungizone and/or Gensumycin
6. Abnormal karyotype or other confirmed genetic syndromes
7. Oncologic disease (previous or current malignancy)
8. Inability to comply with the trial protocol and follow-up schedule
9. Inability to understand the information and to provide informed consent

Prenatal group:

1. Multiple pregnancy
2. Co-existence of other disorder that might interfere with the treatment, as judged by the Investigator or the patient's obstetrician
3. Abnormal fetal karyotype or other confirmed genetic syndrome
4. Any contraindication for invasive procedures such as a bleeding tendency or contagious infections, such as, but not limited to HIV, Syphilis, Hepatitis B, Hepatitis C or other known infectious diseases that can harm the fetus
5. Known risk factors for clotting, such as, but not limited to previous blood clot, family history of clots, clotting disorder (inherited or acquired), heart failure, inflammatory disorders (for example lupus, rheumatoid arthritis, inflammatory bowel disease)
6. Positive Donor Specific Antibody-test
7. Known allergy/hypersensitivity to Fungizone and/or Gensumycin
8. Oncologic disease in woman or fetus (previous or current malignancy)
9. Unwilling to or cannot undergo delivery by Caesarean section
10. Inability to comply with the trial protocol and follow-up schedule
11. Inability to understand the information and to provide informed consent

After birth and before the 2nd dose, subjects in the prenatal group will be assessed for the exclusion criteria for the postnatal group.

Control group:

a) Matched historical controls

1. Existence of other disorder that might interfere with the trial. No lung hypoplasia (type 2 OI).
2. Abnormal karyotype

b) Prospective untreated controls

Postnatal participation: The exclusion criteria, except exclusion criterium 2, 3, 4, and 5 (Contraindication for invasive procedure, Known risk factor for clotting, Positive Donor Specific

Sagar RL, *et al. BMJ Open* 2024; 14:e079767. doi: 10.1136/bmjopen-2023-079767

BOOSTB4

Antibody-test and Known allergy/hypersensitivity to Fungizone and/or Gensumycin), for the postnatal group apply.

Prenatal participation: The exclusion criteria, except exclusion criterium 1, 4, 5, 6 and 7 (Multiple pregnancy, Contraindication for invasive procedure, Known risk factor for clotting, Positive Donor Specific Antibody-test and Known allergy/hypersensitivity to Fungizone and/or Gensumycin), for the prenatal group apply.

MATCHING OF THE CONTROLS

Parents/authorized representatives of children diagnosed with OI type 3 or severe OI type 4 will be informed about the trial and asked to give consent to the child's participation in the trial as a matched historical control. Subjects eligible for the trial but not willing/able to participate in the trial will be asked for consent to be included as prospective controls.

The controls (historical and untreated prospective) will be individually matched to each trial subject:

1. Type of OI*:
   Type 3 with Type 3
   Severe Type 4 with Severe Type 4
   *No mixed OI types (for e.g. OI type 3/4, is allowed)
2. Age at onset of BP therapy:
   Start of BP treatment in the first 2 months of life.
   Start of BP treatment from 2–12 months of life.

If possible (if more than 5 controls have been identified), matching will also be made on:

3. Sex
4. Identical OI mutation
5. Time for diagnosis:
   A control matched with a subject in the postnatal group was diagnosed** postnatally
   A control matched with a subject in the prenatal group was diagnosed** prenatally
   **Clinical diagnosis, molecular diagnosis not required at this time point.

Sagar RL, *et al. BMJ Open* 2024; 14:e079767. doi: 10.1136/bmjopen-2023-079767
