# Supplementary material for: An exploratory open-label multicentre phase I/II trial evaluating the safety and efficacy of postnatal or prenatal and postnatal administration of allogeneic expanded fetal mesenchymal stem cells for the treatment of severe osteogenesis imperfecta in infants and fetuses: the BOOSTB4 trial protocol
Source: BMJ Open. 2024 Jun 4;14(6):e079767. doi: 10.1136/bmjopen-2023-079767 (PMC11163617; doi:10.1136/bmjopen-2023-079767)
Supplement: Supplementary data [file bmjopen-2023-079767supp002.pdf]

BOOSTB4: A clinical research trial of a new intervention for brittle bone disease

SCREENING CONSENT FORM FOR THE POSTNATAL TRIAL GROUP

TO BE COMPLETED BEFORE SCREENING INVESTIGATIONS TAKE PLACE

Consent part 1

I confirm I have read and understood the Participant Information for the above trial. I confirm that I have received sufficient explanation of the BOOSTB4 Clinical Trial and have had the opportunity to consider the information and ask questions which have been answered satisfactorily. I have been given the Participant Information to keep.

I understand that my child's participation is voluntary and that I am free to withdraw them at any time without giving any reason, without their medical care or legal rights being affected.

I give consent for members of the BOOSTB4 Clinical Trial Team to review my child's medical records and use the data contained within, including access to all investigation results relevant for the purposes of this trial.

I give consent for members of the BOOSTB4 Clinical Trial Team to perform a screening investigation and a screening examination of my child as detailed in the Participant Information.

I agree that biological samples collected from my child are a gift, and can be used in the research described in the Participant Information.

I agree that all samples can be stored coded in the Stockholm Medical Biobank (reg. no. 914).

I agree that all coded samples can be transferred to other hospitals or laboratories in other countries for specialist tests.

2 copies required: Original for Investigator Site File and copy for participant. Page 1 of 3

#1 BOOSTB4 Screening Consent Form Postnatal Trial Group [29-10-2020] v1.1 SE to the
#1 BOOSTB4 Participant Information Postnatal Trial Group [29-10-2020] v1.1 SE

Consent part 1

Patient Trial Identification Number: __________________________ (filled in by the trial)

Date of Birth (dd/mm/yyyy) / social security no (personnummer): ___________________

Name of parent	Date (dd/mm/yyyy)	Signature
Name of parent (optional)	Date (dd/mm/yyyy)	Signature
Name of person taking consent	Date (dd/mm/yyyy)	Signature
Name of interpreter (if applicable)	Date (dd/mm/yyyy)	Signature

2 copies required: Original for Investigator Site File and copy for participant.

#1 BOOSTB4 Screening Consent Form Postnatal Trial Group [29-10-2020] v1.1 SE to the
#1 BOOSTB4 Participant Information Postnatal Trial Group [29-10-2020] v1.1 SE

Consent part 2

Voluntary, mark the Yes/No boxes according to your consent.

	YES	NO
I agree that all biological samples can be stored coded at the Stockholm Medical Biobank (reg. no. 914) for future ethically approved research.	☐	☐

Name of parent	Date (dd/mm/yyyy)	Signature
Name of parent (optional)	Date (dd/mm/yyyy)	Signature
Name of person taking consent	Date (dd/mm/yyyy)	Signature
Name of interpreter (if applicable)	Date (dd/mm/yyyy)	Signature

2 copies required: Original for Investigator Site File and copy for participant.

#1 BOOSTB4 Screening Consent Form Postnatal Trial Group [29-10-2020] v1.1 SE to the
#1 BOOSTB4 Participant Information Postnatal Trial Group [29-10-2020] v1.1 SE

BOOSTB4: A clinical research trial of a new intervention for brittle bone disease

MAIN TRIAL CONSENT FORM FOR THE POSTNATAL TRIAL GROUP

TO BE COMPLETED AFTER SCREENING INVESTIGATIONS HAVE BEEN PERFORMED AND ELIGIBILITY CONFIRMED

Consent part 1

I confirm I have read and understood the Participant Information for the above trial. I confirm that I have received sufficient explanation of the BOOSTB4 Clinical Trial and have had the opportunity to consider the information and ask questions which have been answered satisfactorily. I have been given the Participant Information to keep.

I understand that my child's participation is voluntary and that I am free to withdraw them at any time without giving any reason, without their medical care or legal rights being affected.

I understand that relevant sections of my child's medical notes and data collected during the trial may be looked at by responsible individuals from national and international competent authorities, the BOOSTB4 team medical doctors and clinical trial monitors that inspect trial safety, where it is relevant to my child's taking part in this research. I give permission for these individuals to have access to my child's records. This includes medical notes and data collected at the clinical research site, medical notes and data collected within the health care (i.e. the general/family doctor and other community services). I consent to that data from my child are treated as described in the Participant Information.

I agree to my child's doctor and local hospital being informed of my child's participation in the trial. I agree that they can be contacted for medical information or obtaining up-to-date contact details.

I agree to my child taking part in the BOOSTB4 Clinical Trial.

I agree that my child can not participate in another clinical trial before the 12-month follow-up after the last stem cell dose, and that any participation in other trials must be discussed with the trial doctor.

I agree that biological samples collected from my child are a gift as described in the Participant Information.

I agree that all samples can be stored coded in the Stockholm Medical Biobank (reg. no. 914).

2 copies required: Original for Investigator Site File and copy for participant. Page 1 of 3

#2 BOOSTB4 Consent Form Postnatal Trial Group [10-11-2021] v1.2 SE to the
#1 BOOSTB4 Participant Information Postnatal Trial Group [29-10-2020] v1.1 SE

I agree that all coded samples can be transferred to other hospitals or laboratories in other countries for specialist tests.

I understand that this study using the samples may include genetic research aimed at understanding the genes related to OI and how the environment affects them (epigenetics) but that the results of these investigations are unlikely to have any implications for my child personally.

I agree that all discoveries (intellectual property) are a gift to the researchers and understand that my child will not benefit financially if the research leads to a new treatment or medical test.

I agree that I can be contacted by telephone to collect follow-up information about my child.

Consent part 1

Patient Trial Identification Number: ___________________________ (filled in by the trial)

Date of Birth (dd/mm/yyyy) / social security no: __________________________________

Name of parent	Date (dd/mm/yyyy)	Signature
Name of parent (optional)	Date (dd/mm/yyyy)	Signature
Name of person taking consent	Date (dd/mm/yyyy)	Signature
Name of interpreter (if applicable)	Date (dd/mm/yyyy)	Signature

2 copies required: Original for Investigator Site File and copy for participant.

#2 BOOSTB4 Consent Form Postnatal Trial Group [10-11-2021] v1.2 SE to the
#1 BOOSTB4 Participant Information Postnatal Trial Group [29-10-2020] v1.1 SE

Consent part 2

Voluntary, mark the Yes/No boxes according to your consent.	YES	NO
I agree that all biological samples can be stored coded in the Stockholm Medical Biobank (reg. no. 914) for future ethically approved research.	☐	☐
I agree to give consent for photographs and video footage to be taken of trial procedures; the anonymised images may be used for educational purposes or be published.	☐	☐
I agree to give contact details for a relative / friend that the BOOSTB4 trial team can contact if they are unable to get in touch with me in the future.	☐	☐
I agree to the BOOSTB4 trial team contacting me in the future about trial related activities and events.	☐	☐
I would like to receive a summary of the trial results (it will take up to five years).	☐	☐

Name of parent	Date (dd/mm/yyyy)	Signature
Name of parent (optional)	Date (dd/mm/yyyy)	Signature
Name of person taking consent	Date (dd/mm/yyyy)	Signature
Name of interpreter (if applicable)	Date (dd/mm/yyyy)	Signature

2 copies required: Original for Investigator Site File and copy for participant.

#2 BOOSTB4 Consent Form Postnatal Trial Group [10-11-2021] v1.2 SE to the
#1 BOOSTB4 Participant Information Postnatal Trial Group [29-10-2020] v1.1 SE

BOOSTB4: A clinical research trial of a new intervention for brittle bone disease

SCREENING CONSENT FORM FOR THE PRENATAL TRIAL GROUP

TO BE COMPLETED BEFORE SCREENING INVESTIGATIONS TAKE PLACE

Consent part 1

I confirm I have read and understood the Participant Information for the above trial. I confirm that I have received sufficient explanation of the BOOSTB4 Clinical Trial and have had the opportunity to consider the information and ask questions which have been answered satisfactorily. I have been given the Participant Information to keep.

I understand that my participation is voluntary and that I am free to withdraw my consent at any time without giving any reason, without my medical care or legal rights being affected.

I give consent for members of the BOOSTB4 Clinical Trial Team to review my medical records and use the data contained within, including access to all prenatal diagnostic tests. I consent to that information about me is processed as described in the participant information.

I give consent for members of the BOOSTB4 Clinical Trial Team to perform a pre investigation (screening) for the above mentioned trial, including investigations such as ultrasound, as it is described in the participant information.

I agree that biological samples collected from me and my child are a gift, and can be used in the research on stem cell infusion and OI as described in the Participant Information.

I agree that all samples can be stored coded in the Stockholm Medical Biobank (reg. no. 914).

I agree that all coded samples can be transferred to other hospitals or laboratories in other countries for specialist tests.

2 copies required: Original for Investigator Site File and copy for participant.

Sagar RL, *et al. BMJ Open* 2024; 14:e079767. doi: 10.1136/bmjopen-2023-079767

Consent part 1

Patient Trial Identification Number: ____________________________ (filled in by the trial)

Date of Birth (dd/mm/yyyy) / social security number pregnant woman:

____

Name of patient (pregnant woman)	Date (dd/mm/yyyy)	Signature
Name of parent (optional)	Date (dd/mm/yyyy)	Signature
Name of person taking consent	Date (dd/mm/yyyy)	Signature
Name of interpreter (if applicable)	Date (dd/mm/yyyy)	Signature

2 copies required: Original for Investigator Site File and copy for participant.

#3A BOOSTB4 Screening Consent Form Prenatal Trial Group [29-10-2020] v1.1 SE to the
#2 BOOSTB4 Participant Information Prenatal Trial Group [29-10-2020] v1.1 SE

Consent part 2

Voluntary, mark the Yes/No boxes according to your consent.

	YES	NO
I agree that the following samples are used for the development of a non-invasive prenatal test of OI:<br>- A blood sample<br>- Leftover amniotic fluid / placenta / DNA (no new samples are taken)<br>I also agree that the biological samples can be stored coded in Stockholm's medical biobank (reg. No. 914) to be used for research related to the development of a non-invasive prenatal test, as described in the participant information, and that coded samples can sent to hospitals or laboratories in other countries for specific tests.	☐	☐
I agree that all biological samples can be stored coded at the Stockholm Medical Biobank (reg. no. 914) for future ethically approved research.	☐	☐

Name of patient (pregnant woman)	Date (dd/mm/yyyy)	Signature
Name of parent (optional)	Date (dd/mm/yyyy)	Signature
Name of person taking consent	Date (dd/mm/yyyy)	Signature
Name of interpreter (if applicable)	Date (dd/mm/yyyy)	Signature

2 copies required: Original for Investigator Site File and copy for participant.

#3A BOOSTB4 Screening Consent Form Prenatal Trial Group [29-10-2020] v1.1 SE to the
#2 BOOSTB4 Participant Information Prenatal Trial Group [29-10-2020] v1.1 SE

BOOSTB4: A clinical research trial of a new intervention for brittle bone disease

<u>SCREENING</u> CONSENT FORM FOR THE PRENATAL TRIAL GROUP (FATHER)

TO BE COMPLETED BEFORE SCREENING INVESTIGATIONS TAKE PLACE

Consent part 1

I confirm I have read and understood the Participant Information for the above trial. I confirm that I have received sufficient explanation of the BOOSTB4 Clinical Trial and have had the opportunity to consider the information and ask questions which have been answered satisfactorily. I have been given the Participant Information to keep.

I understand that my participation is voluntary and that I am free to withdraw my consent at any time without giving any reason, without my medical care or legal rights being affected.

I give consent for members of the BOOSTB4 Clinical Trial Team to review my medical records and use the data contained within. I consent to that information about me is processed as described in the participant information.

I give consent to donate the following for the development of a non-invasive prenatal test: A blood sample.

I give consent to that the blood sample collected from me may be used for the described research related to the development of a non-invasive prenatal test, as described in the participant information.

I agree that all samples can be stored coded in the Stockholm Medical Biobank (reg. no. 914).

I agree that all coded samples can be transferred to other hospitals or laboratories in other countries for specialist tests.

2 copies required: Original for Investigator Site File and copy for participant.

#2 BOOSTB4 Screening Consent Form Prenatal Trial Group, Father [29-10-2020] v1.1 SE to the
#3B BOOSTB4 Participant Information Prenatal Trial Group [29-10-2020] v1.1 SE

Consent part 1

Patient Trial Identification Number: ___________________________ (filled in by the trial)

Date of Birth (dd/mm/yyyy) / social security number: ______________________________

Name of patient (father)	Date (dd/mm/yyyy)	Signature
Name of person taking consent	Date (dd/mm/yyyy)	Signature
Name of interpreter (if applicable)	Date (dd/mm/yyyy)	Signature

Consent part 2

Voluntary, mark the Yes/No boxes according to your consent.

	YES	NO
I agree that all biological samples can be stored coded at the Stockholm Medical Biobank (reg. no. 914) for future ethically approved research.	☐	☐

Name of patient (father)	Date (dd/mm/yyyy)	Signature
Name of person taking consent	Date (dd/mm/yyyy)	Signature
Name of interpreter (if applicable)	Date (dd/mm/yyyy)	Signature

2 copies required: Original for Investigator Site File and copy for participant. Page 2 of 2

#2 BOOSTB4 Screening Consent Form Prenatal Trial Group, Father [29-10-2020] v1.1 SE to the
#3B BOOSTB4 Participant Information Prenatal Trial Group [29-10-2020] v1.1 SE

BOOSTB4: A clinical research trial of a new intervention for brittle bone disease

MAIN TRIAL CONSENT FORM FOR THE PRENATAL TRIAL GROUP

TO BE COMPLETED AFTER SCREENING INVESTIGATIONS HAVE BEEN PERFORMED AND ELIGIBILITY CONFIRMED

Consent part 1

I confirm I have read and understood the Participant Information for the above trial. I confirm that I have received sufficient explanation of the BOOSTB4 clinical trial and have had the opportunity to consider the information and ask questions which have been answered satisfactorily. I have been given the Participant Information to keep.

I understand that mine and my child's participation is voluntary and that I am free to withdraw them at any time without giving any reason, without our medical care or legal rights being affected.

I understand that relevant sections of mine and my child's medical notes and data collected during the trial may be looked at by responsible individuals from national and international competent authorities, the BOOSTB4 team medical doctors and clinical trial monitors that inspect trial safety, where it is relevant to mine and my child's participation in this research. I give permission for these individuals to have access to mine and my child's records. This includes medical notes and data collected at the clinical research site, medical notes and data collected within the health care (i.e. the general/family doctor and other community services). I consent to that data from me and my child are treated as described in the Participant Information.

I agree to mine and my child's doctor and local hospital being informed of our participation in the trial. I agree that they can be contacted for medical information or obtaining up-to-date contact details.

I agree to me and my child taking part in the BOOSTB4 clinical trial including the follow-up.

I agree that my child maybe can not participate in another clinical trial before the 12-month follow-up after the last stem cell dose, and that any participation in other trials must be discussed with the trial doctor.

I agree that biological samples collected from me and my child, including the placenta, amniotic fluid and umbilical cord, can be used in the described research related to stem cell infusion or OI, as described in the Participant Information.

2 copies required: Original for Investigator Site File and copy for participant.

#4 BOOSTB4 Consent Form Prenatal Trial Group [29-10-2020] v1.1 SE to the
#2 BOOSTB4 Participant Information Prenatal Trial Group [29-10-2020] v1.1 SE

Karolinska Institutet — BOOSTB4 Boost Brittle Bones Before Birth — KAROLINSKA UNIVERSITETSSJUKHUSET

I agree that all samples can be stored coded in the Stockholm Medical Biobank (reg. no. 914).

I agree that all coded samples can be transferred to other hospitals or laboratories in other countries for specialist tests.

I understand that this study using the samples may include genetic research aimed at understanding the genes related to OI and how the environment affects them (epigenetics) but that the results of these investigations are unlikely to have any implications for me or my child personally.

I agree that all discoveries (intellectual property) are a gift to the researchers and understand that I and my child will not benefit financially if the research leads to a new treatment or medical test.

I agree that I can be contacted by telephone to collect follow-up information about myself and my child.

Consent part 1

Patient Trial Identification Number: ___________________________ (filled in by the trial)

Date of Birth (dd/mm/yyyy) / social security number: ______________________________

Name of patient (pregnant woman)	Date (dd/mm/yyyy)	Signature
Name of parent (optional)	Date (dd/mm/yyyy)	Signature
Name of person taking consent	Date (dd/mm/yyyy)	Signature
Name of interpreter (if applicable)	Date (dd/mm/yyyy)	Signature

2 copies required: Original for Investigator Site File and copy for participant.

#4 BOOSTB4 Consent Form Prenatal Trial Group [29-10-2020] v1.1 SE to the
#2 BOOSTB4 Participant Information Prenatal Trial Group [29-10-2020] v1.1 SE

Karolinska Institutet — BOOSTB4 Boost Brittle Bones Before Birth — KAROLINSKA UNIVERSITETSSJUKHUSET

Consent part 2

Voluntary, mark the Yes/No boxes according to your consent.

	YES	NO
I agree that all biological samples can be stored coded in the Stockholm Medical Biobank (reg. no. 914) for future ethically approved research.	☐	☐
I agree to give consent for photographs and video footage to be taken of trial procedures; the anonymised images may be used for educational purposes or be published.	☐	☐
I agree to give contact details for a relative / friend that the BOOSTB4 trial team can contact if they are unable to get in touch with me in the future.	☐	☐
I agree to the BOOSTB4 trial team contacting me in the future about trial related activities and events.	☐	☐
I would like to receive a summary of the trial results (it will take up to five years).	☐	☐

Name of patient (pregnant woman)	Date (dd/mm/yyyy)	Signature
Name of parent (optional)	Date (dd/mm/yyyy)	Signature
Name of person taking consent	Date (dd/mm/yyyy)	Signature
Name of interpreter (if applicable)	Date (dd/mm/yyyy)	Signature

2 copies required: Original for Investigator Site File and copy for participant.

#4 BOOSTB4 Consent Form Prenatal Trial Group [29-10-2020] v1.1 SE to the
#2 BOOSTB4 Participant Information Prenatal Trial Group [29-10-2020] v1.1 SE

BOOSTB4: A clinical research trial of a new intervention for brittle bone disease

POST BIRTH CONSENT CONFIRMATION FOR THE PRENATAL TRIAL GROUP

TO BE COMPLETED AFTER A BABY WHICH RECEIVED BOOSTB4 PRENATALLY HAS BEEN BORN AND ELIGIBILITY RE-CONFIRMED

Consent part 1

I confirm I have read and understood the Participant Information for the above trial. I confirm that I have received sufficient explanation of the BOOSTB4 clinical trial and have had the opportunity to consider the information and ask questions which have been answered satisfactorily. I have been given the Participant Information to keep.

I understand that my child's participation is voluntary and that I am free to withdraw them at any time without giving any reason, without their medical care or legal rights being affected.

I understand that relevant sections of my child's medical notes and data collected during the trial may be looked at by responsible individuals from national and international competent authorities, the BOOSTB4 team medical doctors and clinical trial monitors that inspect trial safety, where it is relevant to my child's taking part in this research. I give permission for these individuals to have access to my child's records. This includes medical notes and data collected at the clinical research site, medical notes and data collected within the health care (i.e. the general/family doctor and other community services). I consent to that data from my child are treated as described in the Participant Information.

I agree to my child's doctor and local hospital being informed of my child's participation in the trial. I agree that they can be contacted for medical information or obtaining up-to-date contact details.

I agree to my child taking part in the BOOSTB4 clinical trial.

I agree that my child can not participate in another clinical trial before the 12-month follow-up after the last stem cell dose, and that any participation in other trials must be discussed with the trial doctor.

I agree that biological samples collected from my child are a gift as described in the Participant Information.

I agree that all samples can be stored coded in the Stockholm Medical Biobank (reg. no. 914).

2 copies required: Original for Investigator Site File and copy for participant.

#5 BOOSTB4 Consent Confirmation Form Prenatal Trial Group [29-10-2020] v1.1 SE to the
#2 BOOSTB4 Participant Information Prenatal Trial Group [29-10-2020] v1.1 SE

I agree that all coded samples can be transferred to other hospitals or laboratories in other countries for specialist tests.

I understand that this study using the samples may include genetic research aimed at understanding the genes related to OI and how the environment affects them (epigenetics) but that the results of these investigations are unlikely to have any implications for my child personally.

I agree that all discoveries (intellectual property) are a gift to the researchers and understand that my child will not benefit financially if the research leads to a new treatment or medical test.

I agree that I can be contacted by telephone to collect follow-up information about my child.

Consent part 1

Patient Trial Identification Number: ____________________________ (filled in by the trial)

Date of Birth (dd/mm/yyyy) / social security number: ________________________________

Name of parent	Date (dd/mm/yyyy)	Signature
Name of parent	Date (dd/mm/yyyy)	Signature
Name of person taking consent	Date (dd/mm/yyyy)	Signature
Name of interpreter (if applicable)	Date (dd/mm/yyyy)	Signature

2 copies required: Original for Investigator Site File and copy for participant. Page 2 of 3

#5 BOOSTB4 Consent Confirmation Form Prenatal Trial Group [29-10-2020] v1.1 SE to the
#2 BOOSTB4 Participant Information Prenatal Trial Group [29-10-2020] v1.1 SE

Consent part 2

Voluntary, mark the Yes/No boxes according to your consent.	YES	NO
I agree that all biological samples can be stored coded in the Stockholm Medical Biobank (reg. no. 914) for future ethically approved research.	☐	☐
I agree to give consent for photographs and video footage to be taken of trial procedures; the anonymised images may be used for educational purposes or be published.	☐	☐
I agree to give contact details for a relative / friend that the BOOSTB4 trial team can contact if they are unable to get in touch with me in the future.	☐	☐
I agree to the BOOSTB4 trial team contacting me in the future about trial related activities and events.	☐	☐
I would like to receive a summary of the trial results (it will take up to five years).	☐	☐

Name of parent	Date (dd/mm/yyyy)	Signature
Name of parent	Date (dd/mm/yyyy)	Signature
Name of person taking consent	Date (dd/mm/yyyy)	Signature
Name of interpreter (if applicable)	Date (dd/mm/yyyy)	Signature

2 copies required: Original for Investigator Site File and copy for participant.

#5 BOOSTB4 Consent Confirmation Form Prenatal Trial Group [29-10-2020] v1.1 SE to the
#2 BOOSTB4 Participant Information Prenatal Trial Group [29-10-2020] v1.1 SE
