# Supplementary material for: An exploratory open-label multicentre phase I/II trial evaluating the safety and efficacy of postnatal or prenatal and postnatal administration of allogeneic expanded fetal mesenchymal stem cells for the treatment of severe osteogenesis imperfecta in infants and fetuses: the BOOSTB4 trial protocol
Source: BMJ Open. 2024 Jun 4;14(6):e079767. doi: 10.1136/bmjopen-2023-079767 (PMC11163617; doi:10.1136/bmjopen-2023-079767)
Supplement: Author's manuscript [file bmjopen-2023-079767.draft_revisions.pdf]

BMJ Open is committed to open peer review. As part of this commitment we make the peer review history of every article we publish publicly available.

When an article is published we post the peer reviewers' comments and the authors' responses online. We also post the versions of the paper that were used during peer review. These are the versions that the peer review comments apply to.

The versions of the paper that follow are the versions that were submitted during the peer review process. They are not the versions of record or the final published versions. They should not be cited or distributed as the published version of this manuscript.

BMJ Open is an open access journal and the full, final, typeset and author-corrected version of record of the manuscript is available on our site with no access controls, subscription charges or pay-per-view fees (http://bmjopen.bmj.com).

If you have any questions on BMJ Open's open peer review process please email info.bmjopen@bmj.com

BMJ Open

An exploratory phase I/II trial evaluating the safety and efficacy of postnatal or pre- and postnatal administration of allogeneic expanded fetal mesenchymal stem cells for the treatment of severe Osteogenesis Imperfecta: The BOOSTB4 Trial Protocol

Journal:	*BMJ Open*
Manuscript ID	bmjopen-2023-079767
Article Type:	Protocol
Date Submitted by the Author:	11-Sep-2023
Complete List of Authors:	Sagar, Rachel; University College London, Elizabeth Garrett Anderson Institute for Women's Health ; NIHR University College London Hospitals Biomedical Research Centre<br>Åström, Eva; Karolinska Institutet, Department of Women's and Children Health; Karolinska University Hospital, Astrid Lindgren Children's Hospital<br>Chitty, Lyn; Great Ormond Street Hospital for Children NHS Foundation Trust, North Thames Genomic Laboratory Hub; UCL Great Ormond Street Institute of Child Health, Genetics and Genomics<br>Crowe, Belinda; Great Ormond Street Hospital for Children NHS Foundation Trust, Department of Neurosciences<br>David, Anna; University College London, Elizabeth Garrett Anderson Institute for Women's Health; NIHR University College London Hospitals Biomedical Research Centre<br>DeVile, Catherine; Great Ormond Street Hospital for Children NHS Foundation Trust, Department of Neurosciences<br>Forsmark, Annabelle ; PharmaLex<br>Franzen, Vera; XNK Therapeutics AB<br>Hermeren, Göran; Lund University Faculty of Medicine, Department of Clinical Sciences<br>Hill, Melissa; Great Ormond Street Hospital For Children NHS Foundation Trust, North Thames Genomic Laboratory Hub; UCL Great Ormond Street Institute of Child Health, Genetics and Genomics<br>johansson, mats; Lund University Faculty of Medicine, Department of Clinical Sciences<br>Lindemans, Caroline; University Medical Centre Utrecht, Department of Pediatrics,<br>Lindgren, Peter; Karolinska University Hospital, Center for Fetal Medicine<br>Nijhuis, Wouter; University Medical Centre Utrecht, Department of Orthopedic Surgery<br>Oepkes, Dick; Leiden University Medical Center, Department of Obstetrics<br>Rehberg, Mirko; University Hospital Cologne, Department of Pediatrics<br>Sahlin, Nils-Eric; Lund University Faculty of Medicine, Department of Clinical Sciences<br>Sakkers, Ralph; University Medical Centre Utrecht, Department of Orthopedic Surgery<br>Semler, O.; University Hospital Cologne, Department of Pediatrics<br>Sundin, Mikael; Karolinska Institutet, Department of Clinical Science,

Intervention and Technology; Karolinska University Hospital, Section of Pediatic Hematology, Immunology and HCT
Walther-Jallow, Lilian; Karolinska Institutet, Department of Clinical Science, Intervention and Technology
Verweij, E.J.T; Leiden University Medical Center, Department of Obstetrics
Westgren, Magnus; Karolinska Institute, Department of Clinical Science, Intervention and Technology
Götherström, Cecilia; Karolinska Institute, Department of Clinical Science, Intervention and Technology

Keywords: Mesenchymal Stem Cells, Cell biology < NATURAL SCIENCE DISCIPLINES, Fetal medicine < OBSTETRICS, Musculoskeletal disorders < ORTHOPAEDIC & TRAUMA SURGERY, Clinical trials < THERAPEUTICS, TRANSPLANT MEDICINE

SCHOLARONE™
Manuscripts

An exploratory phase I/II trial evaluating the safety and efficacy of postnatal or pre- and postnatal administration of allogeneic expanded fetal mesenchymal stem cells for the treatment of severe Osteogenesis Imperfecta: The BOOSTB4 Trial Protocol

Rachel L Sagar (rachelsagar@nhs.net),[1,2]* Eva Åström (eva.astrom@regionstockholm.se),[3,4]* Lyn S Chitty (l.chitty@ucl.ac.uk),[5,6] Belinda Crowe (Belinda.Crowe@gosh.nhs.uk),[7] Anna L David (a.david@ucl.ac.uk),[1,2] Catherine DeVile (Catherine.DeVile@gosh.nhs.uk),[7] Annabelle Forsmark (annabelle.forsmark@pharmalex.com),[8] Vera Franzén (vera.frz@telia.com),[9] Göran Hermerén (goran.hermeren@med.lu.se),[10] Melissa Hill (melissa.hill@ucl.ac.uk),[5,6] Mats Johansson (mats.johansson@med.lu.se),[10] Caroline A Lindemans (C.A.Lindemans@prinsesmaximacentrum.nl),[11] Peter Lindgren (peter.lindgren@regionstockholm.se),[12] Wouter H Nijhuis (W.H.Nijhuis-2@umcutrecht.nl),[13] Dick Oepkes (D.Oepkes@lumc.nl),[14] Mirko Rehberg (mirko.rehberg@uk-koeln.de),[15] Nils-Eric Sahlin (nils-eric.sahlin@med.lu.se),[10] Ralph Sakkers (Sakkers@umcutrecht.nl),[13] Oliver Semler (joerg.semler@uk-koeln.de),[15] Mikael Sundin (mikael.sundin@ki.se),[16,17] Lilian Walther-Jallow (lilian.walther.jallow@ki.se),[18] E.J.T. (Joanne) Verweij (e.j.t.verweij@lumc.nl),[14] Magnus Westgren (magnus.westgren@ki.se)[18] and Cecilia Götherström (cecilia.gotherstrom@ki.se)[18]

*Shared first authorship.

[1]Elizabeth Garrett Anderson Institute for Women's Health, University College London, London, United Kingdom.

[2]NIHR University College London Hospitals Biomedical Research Centre, London, United Kingdom.

[3]Neuropediatric Unit, Astrid Lindgren Children's Hospital, Karolinska University Hospital, Stockholm, Sweden.

[4]Neuropediatric Unit, Department of Women's and Children Health, Karolinska Institutet, Stockholm, Sweden.

[5]North Thames Genomic Laboratory Hub, Great Ormond Street NHS Foundation Trust, London, United Kingdom.

[6]Genetics and Genomics, UCL Great Ormond Street Institute of Child Health, London, United Kingdom.

[7]Department of Neurosciences, Great Ormond Street Hospital for Children NHS Foundation Trust, London, United Kingdom.

[8]PharmaLex, Gothenburg, Sweden.

For peer review only - http://bmjopen.bmj.com/site/about/guidelines.xhtml

[9]XNK Therapeutics AB, Huddinge, Sweden.

[10]Medical Ethics, Department of Clinical Sciences, Lund, Faculty of Medicine, Lund University, Lund, Sweden.

[11]Department of Pediatrics, University Medical Center Utrecht, Utrecht, The Netherlands.

[12]Center for Fetal Medicine, Karolinska University Hospital, Stockholm, Sweden.

[13]Department of Orthopedic Surgery, University Medical Centre Utrecht, Wilhelmina Children's Hospital, Utrecht, The Netherlands.

[14]Department of Obstetrics, Division of Fetal Therapy, Leiden University Medical Center, Leiden, The Netherlands.

[15]Department of Pediatrics, Faculty of Medicine, University of Cologne and University Hospital Cologne, Cologne, Germany.

[16]Division of Pediatrics, Department of Clinical Science, Intervention and Technology, Karolinska Institutet, Stockholm, Sweden.

[17]Section of Pediatic Hematology, Immunology and HCT, Astrid Lindgren Children's Hospital, Karolinska University Hospital, Stockholm, Sweden.

[18]Division of Obstetrics and Gynecology, Department of Clinical Science, Intervention and Technology, Karolinska Institutet, Stockholm, Sweden.

Corresponding author: Cecilia Götherström, Division of Obstetrics and Gynecology, Department of Clinical Science, Intervention and Technology, Karolinska Institutet, ANA Futura, Alfred Nobels Allé 8, 141 52 Huddinge, Sweden. Phone +4670-4712300

Keywords: Osteogenesis Imperfecta; Brittle Bone Disease; Mesenchymal Stem Cells; Transplantation; Clinical Trial.

Word count: 3572 (not including the title page, abstract, references, figures, tables or acknowledgments.)

For peer review only

ABSTRACT

Introduction: Severe Osteogenesis Imperfecta (OI) is a debilitating disease with no cure or sufficiently effective treatment. Mesenchymal stem cells (MSCs) have good safety profile, show promising effects and can form bone. The Boost Brittle Bones Before Birth (BOOSTB4) trial evaluates administration of allogeneic expanded human first trimester fetal liver MSCs (BOOST cells) for OI type III or severe type IV.

Methods and analysis: BOOSTB4 is an exploratory, open label, multiple dose, phase I/II clinical trial evaluating safety and efficacy of postnatal (n=15) or prenatal and postnatal (n=15) administration of BOOST cells for the treatment of severe OI compared with a combination of historical (1–5 per subject) and untreated prospective controls (≥30). Infants <18 months of age and singleton pregnant women whose fetus has severe OI with confirmed glycine substitution in *COL1A1* or *COL1A2* can be included in the trial.

Each subject receives four intravenous doses of $3x10^6$/kg BOOST cells at 4-month intervals, with 48 (dose 1–2) or 24 (dose 3–4) hours in-patient monitoring, primary follow-up at 6 and 12 months after the last dose, and long-term follow-up yearly until 10 years after the first dose. Prenatal subjects receive the 1st dose via ultrasound-guided intrahepatic umbilical vein injection (16+0 to 35+6 weeks), and three doses postnatally.

The primary outcome measures are safety and tolerability of repeated BOOST cell administration. The secondary outcome measures are the number of fractures from baseline to primary and long-term follow-up, growth, change in bone mineral density, clinical OI status and biochemical bone turnover.

Ethics and dissemination: The trial is approved by competent authorities in Sweden, the United Kingdom and the Netherlands (postnatal part). Results from the trial will be disseminated on EudraCT and ClinicalTrials.gov and published in scientific open access journals.

Trial registration numbers: EudraCT (2015-00369-60), ClinicalTrials.gov (NCT03706482); Pre-results.

For peer review only

For peer review only - http://bmjopen.bmj.com/site/about/guidelines.xhtml

Strengths and limitations of this study:

- This is a protocol for an exploratory, open label, multiple dose, multi-centre phase I/II clinical trial investigating the advanced therapy medicinal product BOOST cells.
- This is the first clinical trial to assess whether postnatal or pre- and postnatal administration of BOOST cells is a safe and potentially effective treatment option for severe OI.
- We plan to recruit 15 postnatal and 15 prenatal subjects, and we have designed this trial to assess safety.
- The trial may be underpowered to show improvements in efficacy. Therefore, if the trial yields a positive result, a larger subsequent multi-centre pivotal trial may be needed.

For peer review only

For peer review only - http://bmjopen.bmj.com/site/about/guidelines.xhtml

INTRODUCTION

Osteogenesis imperfecta (OI) is a rare clinically and genetically heterogenous group of heritable disorders of connective tissue characterised by osteopenia, growth restriction, bone deformities, fractures and chronic pain.[1] Originally, OI was classified into four types according to clinical, radiological and histological findings, where type I refers to the mildest form, type II to the perinatally lethal form, type III to the most severe form compatible with life, and type IV to an intermediate group with moderate to severe types of OI.[2] This classification covers 85–90% of persons with OI and includes autosomal dominant sequence variations in the genes coding for type I collagen (*COL1A1* or *COL1A2*).[1-3] There is currently no curative treatment for OI, with the only available pharmaceutical treatments failing to address the underlying molecular pathology, the collagen defect.[4] Treatment is aimed at increasing overall bone strength to prevent fractures and maintain mobility. Bisphosphonates are used to reduce bone resorption and increase bone mass.[5] However, two meta-analyses of randomised trials on bisphosphonate treatment for OI did not demonstrate improvement in fracture rates, reduction of pain or improved functional mobility, although multiple studies report this independently.[6 7]

One child among 10–20,000 is born with OI. Diagnosis of severe types of OI is often made at the routine fetal anomaly ultrasound scan mid-pregnancy, or early if first trimester scanning is routine. Early treatment of OI is preferred to minimise irreversible pathology. Prenatal intervention offers multiple potential advantages over postnatal approaches; ameliorating and preventing damage that has begun already during fetal life, the relatively naive fetal immune system may permit the development of immune tolerance towards donor cells, infusion into the systemic fetal circulation may avoid sequestration of the cells in the lungs because circulatory shunts allow the pulmonary circulation to be bypassed, fetal life is a time of natural stem cell proliferation and migration to different anatomic compartments. Also, prenatal administration may enhance the effect of MSCs because there will be a lesser amount of poor quality OI type collagen that requires replacement, and

For peer review only - http://bmjopen.bmj.com/site/about/guidelines.xhtml

lastly, the possible psychological benefits for parents of commencing treatment as early in the child's life.[8]

The investigational medicinal product (IMP) that is investigated in the BOOSTB4 trial is an advanced therapy medicinal product (ATMP) consisting of allogeneic *ex vivo* expanded human fetal liver-derived mesenchymal stem cells (MSCs) from the first trimester of pregnancy. MSCs are multipotent stem cells and are promising candidates in regenerative medicine due to their low immunogenic profile that allows the possibility of performing cell transplantation across major histocompatibility barriers without immunosuppression.[9 10] In addition, they have the potential to differentiate along many lineages and have a good safety profile with minimal oncogenic risk,[11-15] and with almost no infusion toxicity reported.[9 16] The first clinical infusion of MSCs was performed over 20 years ago and adult MSCs have since been used in a diverse range of conditions in thousands of patients.[9 16] Studies show that MSCs derived from developmentally younger donor sources present characteristics that suggest a stronger cell therapeutic potential than adult MSCs, and this knowledge is being translated into clinical application. First trimester fetal MSCs are found at a higher frequency, are less mature and have a greater colony-forming and proliferation capacity compared to adult MSCs.[17 18] Interestingly, fetal MSCs differentiate more readily into bone than adult MSCs, and in a direct comparison of fetal MSCs and MSC derived from Wharton's Jelly, adult bone marrow and adipose tissue, fetal MSCs had higher levels of osteogenic genes under basal conditions. Upon osteogenic differentiation, fetal MSCs produced more robust osteogenic gene expression, induced more calcium deposition *in vitro* and reached higher levels of osteogenic gene upregulation *in vitro* and *in vivo*.[19-21]

The potential of MSC transplantation for the treatment of OI has been shown in different mouse models of OI, with demonstrated homing and engraftment in bone, increased collagen content and mineralisation of the bones, improved bone strength, thickness and length, and 67–84% reduction in long bone fractures.[22-24] Clinically, two studies on intravenous administration of histocompatible MSCs from sibling donors to children with severe OI have been performed (6 and 2 children treated

For peer review only

For peer review only - http://bmjopen.bmj.com/site/about/guidelines.xhtml

respectively).[25 26] The procedures were safe with no serious adverse events (SAEs) reported, and positive clinical effects were attained with donor cell engraftment, development of new compact bone, improved bone parameters, and six months following two MSC infusions there was an increase in growth velocity, an 80% reduction in fracture frequency, an increase in total bone mineral content[25] and in Quality of Life.[26 27]

We have previously reported two case studies of prenatal and postnatal transplantation of fetal MSCs for severe OI (type III and type IV OI).[28 29] The limited data show that the procedures were safe and clinically promising when comparing the clinical course to other individuals with identical OI sequence variants. Only three other individuals in the world are currently known to have a *COL1A2* variant identical to that of the patient with OI type III; one of whom died from the severe type of OI despite bisphosphonate treatment, and two individuals are described as having type II/III OI.[30 31] After one dose of fetal MSCs prenatally, donor cells were found in the recipient bones at nine months of age, and the donor cells expressed bone markers.[27] Four booster doses with same donor MSCs between 8–13 years of age show a reduction in fracture rate (from 2/year to 0/year over two years), and an increase in height, albeit 5.0–6.4 standard deviations below the mean.[28] While it is difficult to determine the definitive impact of the prenatal and postnatal MSC transplantation in these two heterogeneous cases, the findings suggest a potential clinical benefit. The children have now been followed up for over 21 and 14 years, respectively, and there has been no evidence of any early or late adverse reactions.

There is increasing interest in treatment with MSCs and a growing number of requests from patients and their physicians to provide it. In the absence of a clinical trial, prenatal and postnatal MSC transplantation for OI is likely to be practised around the world on a case-by-case basis, making it impossible to evaluate any effects the intervention might have. Considering the complexity of this field and the distribution of cases, an international trial is the only realistic and ethically correct

For peer review only - http://bmjopen.bmj.com/site/about/guidelines.xhtml

approach to enable accurate evaluation of the intervention. The BOOSTB4 trial will provide this well-defined framework for assessment of children and fetuses with OI treated with fetal MSCs.

For peer review only

For peer review only - http://bmjopen.bmj.com/site/about/guidelines.xhtml

METHODS AND ANALYSIS

Trial design

BOOST Brittle Bones Before Birth (BOOSTB4) is an exploratory, open label, multiple dose, phase I/II trial evaluating safety and efficacy of four postnatal intravenous doses (n=15) or one prenatal and three postnatal intravenous doses (n=15) of allogeneic expanded fetal MSCs (BOOST cells) for the treatment of severe OI compared with a combination of historical (1–5/subject) and up to 30 untreated prospective controls.

Eligibility and recruitment

The inclusion and exclusion criteria for the three trial groups are described in Table 1. Information about the BOOSTB4 trial has been distributed to specialist OI services and fetal medicine units in Europe, and the BOOSTB4 trial team can also be contacted directly by families or physicians via email or the trial specific website boostb4.eu where the Patient Information Leaflet is available in several languages. The potential patients are invited to visit the trial site or have an organized video meeting and provide their oral and written consent to be screened for participation. At least three days will be required for the parents to consider participation in the trial. For all children enrolled in the trial, a signed informed-consent form for the main trial is required from the mother and the father, if she/he has parental responsibility. For prenatal inclusion, only the pregnant woman needs to sign the informed-consent form, but both parents are encouraged to sign. After the birth of a child in the prenatal group the inclusion and exclusion criteria for the postnatal group must be fulfilled (Table 1), and both parents must re-consent their participation in the trial before the first postnatal dose. Consent may be withdrawn at any time without a reason being stated.

Sample size calculation

No formal power calculations have been performed. OI is a rare disease, and a limited number of subjects can be included each year. Based on the incidence, it is reasonable to include 30 patients (15

For peer review only - http://bmjopen.bmj.com/site/about/guidelines.xhtml

postnatal subjects and 15 prenatal subjects) in this first trial on the grounds of feasibility, suitable time period and for the application of descriptive statistical analyses.

Blinding, randomisation and allocation

It would not be ethically appropriate to perform a randomised double-blind placebo-control trial in this population, particularly regarding the risks of the prenatal infusion procedure and the vulnerability of the subjects. No blinding or randomisation will therefore take place.

One to five historical and/or prospective controls in the trial will be individually matched to each trial subject with the following matching criteria; **1)** Type of OI (type III with type III and severe type IV with severe type IV) and **2)** Age at onset of bisphosphonate therapy (start of bisphosphonate treatment in the first 2 months of life, or start of bisphosphonate treatment from 2–12 months of life), and if possible (if more than five historical controls have been identified) matching will also be made on **3)** Sex, **4)** Identical OI mutation, and **5)** Time for diagnosis (a control matched with a subject in the postnatal group was diagnosed postnatally, and a control matched with a subject in the prenatal group was diagnosed prenatally).

Intervention protocol

Four intravenous doses of BOOST cells are administered at four-month intervals (range 3–5 months). For postnatal subjects the 1st dose must be administered before 18 months of age. For prenatal subjects, the 1st dose will be given *in utero* during gestational weeks+days 16+0–35+6 via an intrahepatic umbilical vein infusion, and the final three doses are administered postnatally. Each dose contains $3x10^6$ allogeneic expanded human fetal liver MSCs per kg bodyweight.

In-patient monitoring is performed over 48 (dose 1–2) or 24 (dose 3–4) hours in the postnatal group and over 48 (dose 1–3) or 24 (dose 4) hours in the prenatal group after each dose. Primary follow-up is performed at 6 and 12 months after the last dose, and long-term follow up is performed yearly until

For peer review only - http://bmjopen.bmj.com/site/about/guidelines.xhtml

10 years after the first dose. At each visit and follow-up, AEs and parameters related to efficacy outcomes are recorded. Women in the prenatal group are followed under the same schedule.

Outcome measures

The primary endpoint of the BOOSTB4 trial is safety and tolerability measured as seriousness, severity and frequency of administration-related adverse events (AEs) in the child, fetus and woman after four postnatal or one prenatal and three postnatal intravenous doses of BOOST cells every four months. Specific focus will be given to vital signs, transfusion and immune reactions and maternal and fetal AEs using the Maternal and Fetal Adverse Event Terminology,[32] for example, persistent fetal bradycardia following the infusion, tumorigenicity, morbidity and mortality.

The secondary endpoints relate to efficacy of four doses of BOOST cells in children with OI. The hypothesis under investigation is that early administration of BOOST cells will ameliorate severe types of OI. The key secondary endpoint is number of fractures from baseline to primary (12 months after the last dose) and long-term (10 years after the first dose) follow-up. Fracture frequency was chosen as the key secondary endpoint because it is the most clinically relevant efficacy variable. Additional secondary endpoints are time to first fracture after each BOOST cell administration, number of fractures at birth in the prenatal group, growth (length/height and weight), change in bone mineral density measured with DXA/DEXA, clinical OI status and biochemical bone turnover in peripheral blood.

The exploratory endpoints investigated by the trial include assessing the impact of the therapy on Quality of Life using the Infant Toddler Quality of Life Questionnaire™ (ITQOL), studying the paracrine effect and the effect on endogenous immune cells of BOOST cells, studying the extent of donor cell engraftment in tissue samples, and development of a non-invasive method for prenatal diagnosis of OI (not used as the diagnostic method in the trial). In addition, there will be a sub-study to BOOSTB4, where parents will have the option to take part in two semi-structured interviews, in which their experiences of their participation in the trial are explored. This will allow us to understand their

For peer review only - http://bmjopen.bmj.com/site/about/guidelines.xhtml

reasoning for their child to participate in the trial, and what has been positive and what can be improved in future similar studies.

Setting

The Sponsor for this academic trial is Karolinska Institutet in Sweden. The trial sites are five maternity and paediatric institutions; Karolinska University Hospital in Sweden, University College London and Great Ormond Street Hospital for Children in the United Kingdom, and Leiden University Medical Center and University Medical Center Utrecht in the Netherlands. The trial has ethical and regulatory approval in Sweden (2018/1732-31, 5.1-2018-57411), United Kingdom (19/NE/0030, 44119/0002/001-001), Netherlands (NL64105.000-17, postnatal part) and NHS Research Ethics Committee approval (16/NS/0084) for the interview study in the United Kingdom.

Statistical analysis

The primary endpoint of the BOOSTB4 trial is the safety of postnatal or prenatal and postnatal administration of BOOST cells. It involves investigation of the seriousness, severity and frequency of treatment-related AEs in the child, woman and fetus. All reported AEs (regardless of the cause) will be included in the statistical analysis, and all analyses will be performed separately for the infant/child, fetus and pregnant woman.

The BOOSTB4 trial is a non-randomised trial and specific caution will be taken to control for problems with bias. The statistical analysis will include descriptive statistics of the data that will be discussed in relation to the general knowledge of the condition, to generate an overall picture of the safety and efficacy profile of the treatment. All trial subjects will be compared to himself or herself as his or her own control over time (longitudinally from baseline to the primary follow-up at 12 months after the last dose and to the long-term follow-up until 10 years after the first dose). One to five matched historical controls (30–150 in total) will, as far as possible, be included per trial subject to increase the statistical precision, and up to 30 prospective untreated controls will also be included.

For peer review only - http://bmjopen.bmj.com/site/about/guidelines.xhtml
For peer review only

Patient and public involvement

The trial has been discussed with patient support groups including the OI Federation Europe (OIFE) and Care 4 Brittle Bones (C4BB) from early in the planning stage. We are grateful for their input, and for the joint dissemination of the trial in 2019.

In a separate study we explored stakeholder views to understand perceived benefits or concerns, identify ethical issues and establish protocols for support and counselling. Semi-structured qualitative interviews were conducted with 56 participants from three groups: adults affected with OI, with and without children, and parents of children affected with OI, health professionals who work with patients with OI, and patient advocates from relevant patient support groups.[8] There were generally positive views towards using fetal MSC transplantation to treat OI, with early treatment considered to be advantageous for preventing fractures and reducing severity, as well as potentially bringing psychological benefits for parents. Participants were somewhat concerned about procedure safety, short and long-term side effects and the effectiveness of transplantation. The difficulties inherent in decision-making were frequently raised, as well as the importance of explaining fetal MSC transplantation in a way that all parents can understand, setting clear expectations, providing psychological support and allowing time to reflect during the decision-making process. The feedback received has been incorporated into the design and planning of the BOOSTB4 trial.

Health economic assessment

A health economic assessment is being undertaken with the objective to describe the costs and effects of BOOST cell administration as a potential standard treatment for severe forms of OI. Published data on the impact of OI on healthcare burden are scarce,[33] with available studies reporting a high cost for e.g. frequent hospital admissions.[34 35] Also, two sets of questionnaires have been developed to capture dependency on healthcare and/or community services, with one focusing on patient/family (postnatal and prenatal groups) and the other on maternal health (prenatal group). These questionnaires will be

For peer review only - http://bmjopen.bmj.com/site/about/guidelines.xhtml

used to further assess the costs and effects of BOOST cell transplantation for severe forms of OI, in addition to further exploring the economic burden of OI overall.

Trial status

Recruitment commenced in Sweden after the site opened on 12th of August 2019. The first subject in the postnatal group was included and received the 1st dose on 17th of March 2020. Due to the SARS-CoV-2 pandemic only two subjects were included in the trial during 2020, and due to time restrictions for the project, the dose administrations are only performed at Karolinska University Hospital in Sweden. After a safety assessment by the Data and Safety Monitoring Board of at least one dose administered to the 1st five postnatal subjects in the trial, the prenatal group of the trial was opened 17th of June 2021, and the 1st prenatal dose was administered on 18th of November 2021. By 2nd of March 2022, all 15 of 15 subjects in the postnatal group had been included and received their 1st dose, and by 14th of December 2021, three of 15 prenatal subjects had been included and administered with one prenatal dose each. No more subjects will be included in the prenatal group due to time restrictions in funding of the trial. The primary follow-up is currently being performed and is expected to be completed in January 2024, and the long-term follow-up will be completed in 2032.

ETHICS

The BOOSTB4 clinical trial aims to investigate fetal-derived stem cell treatment in young children and unborn children (which includes the pregnant woman as a research subject) suffering from a chronic disease. Several ethical issues arise when considering stem cell transplantation for OI in these study populations. Therefore, a detailed and systematic risk – benefit assessment was performed before initiation of the trial, which is updated when needed, for example during the COVID pandemic. The assessment considers many areas; risks related to handling, administration and exposure to the IMP, and risks for the child, fetus and pregnant woman. The assessment also includes potential benefits for the child, fetus and the parents. All risks and benefits are discussed in relation to the Sponsor's

For peer review only

For peer review only - http://bmjopen.bmj.com/site/about/guidelines.xhtml

experience with administration of fetal MSCs for treatment of OI. In other words, this therapy is believed to be directly beneficial to the fetus and child, but there are also significant unknowns.

In the BOOSTB4 study there have been several separate considerations of diverse ethical issues. They include reviewing the ethical and legal standards relevant to prenatal and postnatal stem cell transplantation, mapping the rights and interests of the research subjects, investigating stakeholder's views,[8] as well as testing the Patient Information Leaflet and the communication of risks.

Identifying the main stakeholders is fundamental since the safety and rights of the research subjects should be the main concern. At the prenatal stage the main stakeholders are the pregnant woman and the fetus. At the postnatal stage, only the minor is the research subject, but the parents are also stakeholders, since the infant cannot consent to the participation. We have also discussed the risk of overprotection of pregnant women since they are considered to be a vulnerable population. This may violate their autonomy, harm their interests, and prevent research that could help children and possibly improve their own Quality of Life. Overprotection may signal good intentions and an ambition to minimise risks, but it could also result from a tendency to underestimate the benefits the therapy may bring to the women from whom consent is being sought. Partner consent is not legally required prenatally, although there are exceptions (discussed in [36]). Nonetheless, it is recommended if the partner is expected to become the child's legal representative after birth. It will also be impossible to complete the trial if the partner dissents after birth, and the trial concerns the partner in many ways.

As a sub-study to BOOSTB4, the parents can consent to take part in two qualitative interviews. This will allow us to understand their reasoning for their child to participate in the trial, and what has been positive and what can be improved in future similar studies.

DISSEMINATION

Trial results will be uploaded on EudraCT and ClinicalTrials.gov and published in scientific open access journals when the primary follow-up has been completed, and also when and the long-term follow-

For peer review only - http://bmjopen.bmj.com/site/about/guidelines.xhtml

up has been completed. Lay summaries of the findings will be shared via the networks of the OI patient organisations OIFE and C4BB, and distributed to the families in the BOOSTB4 trial that have opted to receive such summaries. Details of the trial and progress will be shared by presentation at relevant national and international meetings.

Authors' contributions

CG, MW, EÅ, OS and ALD conceived the idea for the trial. All authors developed the trial protocol. CG, MW, EÅ, and LWJ wrote the initial trial protocol, which was subsequently revised by all authors. GH, MJ and NES wrote the initial texts on ethics and regulatory issues. EÅ developed the clinical practice of OI follow-up. MS developed the clinical practice and clinical safety parameters, and developed the study database. MH and LSC designed the research with stakeholders, MH conducted the research with stakeholders and contributed to the development of parent facing study materials. RS, EÅ and CG wrote the draft of the manuscript and all authors revised, reviewed and approved the final manuscript.

Funding statement

This project has received funding from the European Union's Horizon 2020 research and innovation programme under grant agreement No 681045, the Swedish Research Council (921-2014-7209, and 2020-01666), Region Stockholm in Sweden (20200365, FoUI-975495), the Center for Medical Innovation in Sweden (FoUI-962010), and the Foundation for Children of the Freemasons in Stockholm. Parts of this work were supported by grants from the German Science Foundation (Deutsche Forschungsgemeinschaft, DFG) to the Research Unit FOR 2722; Refnr SE2373/1-2, Project 384170921. LSC is partially funded by the NIHR Great Ormond Street Hospital (GOSH) Biomedical Research Centre (BRC). All research at Great Ormond Street Hospital NHS Foundation Trust and UCL Great Ormond Street Institute of Child Health is made possible by the NIHR Great Ormond Street

For peer review only - http://bmjopen.bmj.com/site/about/guidelines.xhtml

Hospital Biomedical Research Centre. The views expressed are those of the authors and not necessarily those of the NHS, the NIHR, or the UK Department of Health.

Competing interest statement

ALD is a consultant for Esperare Foundation for a clinical trial unrelated to this work. CG, LWJ and MW are co-founders and co-owners of BOOST Pharma ApS founded in 2020. OS is a scientific advisor for BOOST Pharma ApS. AF, BC, CAL, CDV, DO, EJTV, EÅ, GH, LSC, MH, MJ, MR, MS, NES, PL, RLS, RS, VF and WN declare no competing interests.

Data statement

Trial data is found at ClinicalTrials.gov ID NCT03706482.

Acknowledgements

We thank all families taking part in the BOOSTB4 trial, and physicians collaborating in accomplishing the trial. We also thank OIFE and C4BB for their never-ending support.

For peer review only

For peer review only - http://bmjopen.bmj.com/site/about/guidelines.xhtml

REFERENCES

1. Van Dijk FS, Sillence DO. Osteogenesis imperfecta: clinical diagnosis, nomenclature and severity assessment. *Am J Med Genet A* 2014;164A:1470-81.

2. Sillence DO, Senn A, Danks DM. Genetic heterogeneity in osteogenesis imperfecta. *J Med Genet* 1979;16:101-16.

3. Van Dijk FS, Pals G, Van Rijn RR, et al. Classification of Osteogenesis Imperfecta revisited. *Eur J Med Genet* 2010;53:1-5.

4. Thomas IH, DiMeglio LA. Advances in the Classification and Treatment of Osteogenesis Imperfecta. *Curr Osteoporos Rep* 2016;14:1-9.

5. Glorieux FH, Bishop NJ, Plotkin H, et al. Cyclic administration of pamidronate in children with severe osteogenesis imperfecta. *N Engl J Med* 1998;339:947-52.

6. Dwan K, Phillipi CA, Steiner RD, et al. Bisphosphonate therapy for osteogenesis imperfecta. *Cochrane Database Syst Rev* 2014;7:CD005088.

7. Hald JD, Evangelou E, Langdahl BL, et al. Bisphosphonates for the prevention of fractures in osteogenesis imperfecta: meta-analysis of placebo-controlled trials. *J Bone Miner Res* 2015;30:929-33.

8. Hill M, Lewis C, Riddington M, et al. Stakeholder views and attitudes towards prenatal and postnatal transplantation of fetal mesenchymal stem cells to treat Osteogenesis Imperfecta. *Eur J Hum Genet* 2019;27:1244-53.

9. Squillaro T, Peluso G, Galderisi U. Clinical Trials With Mesenchymal Stem Cells: An Update. *Cell Transplant* 2016;25:829-48.

10. Götherström C, Ringden O, Westgren M, et al. Immunomodulatory effects of human foetal liver-derived mesenchymal stem cells. *Bone Marrow Transplant* 2003;32:265-72.

11. Prockop DJ, Brenner M, Fibbe WE, et al. Defining the risks of mesenchymal stromal cell therapy. *Cytotherapy* 2010;12:576-8.

12. Pittenger MF, Mackay AM, Beck SC, et al. Multilineage potential of adult human mesenchymal stem cells. *Science* 1999;284:143-7.

13. Bernardo ME, Zaffaroni N, Novara F, et al. Human bone marrow derived mesenchymal stem cells do not undergo transformation after long-term in vitro culture and do not exhibit telomere maintenance mechanisms. *Cancer Res* 2007;67:9142-9.

14. Barkholt L, Flory E, Jekerle V, et al. Risk of tumorigenicity in mesenchymal stromal cell-based therapies –bridging scientific observations and regulatory viewpoints. *Cytotherapy* 2013;15:753-9.

15. von Bahr L, Batsis I, Moll G, et al. Analysis of tissues following mesenchymal stromal cell therapy in humans indicates limited long-term engraftment and no ectopic tissue formation. *Stem Cells* 2012;30:1575-8.

For peer review only

For peer review only - http://bmjopen.bmj.com/site/about/guidelines.xhtml

16. Lalu MM, McIntyre L, Pugliese C, et al. Safety of cell therapy with mesenchymal stromal cells (SafeCell): a systematic review and meta-analysis of clinical trials. *PLoS One* 2012;7:e47559.

17. Guillot PV, Gotherstrom C, Chan J, et al. Human first-trimester fetal MSC express pluripotency markers and grow faster and have longer telomeres than adult MSC. *Stem Cells* 2007;25:646-54.

18. Götherström C, West A, Liden J, et al. Difference in gene expression between human fetal liver and adult bone marrow mesenchymal stem cells. *Haematologica* 2005;90:1017-26.

19. Zhang ZY, Teoh SH, Chong MS, et al. Superior osteogenic capacity for bone tissue engineering of fetal compared with perinatal and adult mesenchymal stem cells. *Stem Cells* 2009;27:126-37.

20. Guillot PV, De Bari C, Dell'Accio F, et al. Comparative osteogenic transcription profiling of various fetal and adult mesenchymal stem cell sources. *Differentiation* 2008;76:946-57.

21. Zhang ZY, Teoh SH, Chong MS, et al. Neo-vascularization and bone formation mediated by fetal mesenchymal stem cell tissue-engineered bone grafts in critical-size femoral defects. *Biomaterials* 2010

22. Guillot PV, Abass O, Bassett JH, et al. Intrauterine transplantation of human fetal mesenchymal stem cells from first-trimester blood repairs bone and reduces fractures in osteogenesis imperfecta mice. *Blood* 2008;111:1717-25.

23. Panaroni C, Gioia R, Lupi A, et al. In utero transplantation of adult bone marrow decreases perinatal lethality and rescues the bone phenotype in the knockin murine model for classical, dominant osteogenesis imperfecta. *Blood* 2009;114:459-68.

24. Vanleene M, Saldanha Z, Cloyd KL, et al. Transplantation of human fetal blood stem cells in the osteogenesis imperfecta mouse leads to improvement in multiscale tissue properties. *Blood* 2011;117:1053-60.

25. Horwitz EM, Gordon PL, Koo WK, et al. Isolated allogeneic bone marrow-derived mesenchymal cells engraft and stimulate growth in children with osteogenesis imperfecta: Implications for cell therapy of bone. *Proc Natl Acad Sci U S A* 2002;99:8932-7.

26. Infante A, Gener B, Vazquez M, et al. Reiterative infusions of MSCs improve pediatric osteogenesis imperfecta eliciting a pro-osteogenic paracrine response: TERCELOI clinical trial. *Clin Transl Med* 2021;11:e265.

27. Infante A, Cabodevilla L, Gener B, et al. Circulating TGF-beta Pathway in Osteogenesis Imperfecta Pediatric Patients Subjected to MSCs-Based Cell Therapy. *Front Cell Dev Biol* 2022;10:830928.

28. Götherström C, Westgren M, Shaw SW, et al. Pre- and postnatal transplantation of fetal mesenchymal stem cells in osteogenesis imperfecta: a two-center experience. *Stem Cells Transl Med* 2014;3:255-64.

29. Le Blanc K, Gotherstrom C, Ringden O, et al. Fetal mesenchymal stem-cell engraftment in bone after in utero transplantation in a patient with severe osteogenesis imperfecta. *Transplantation* 2005;79:1607-14.

For peer review only - http://bmjopen.bmj.com/site/about/guidelines.xhtml

30. Pollitt R, McMahon R, Nunn J, et al. Mutation analysis of COL1A1 and COL1A2 in patients diagnosed with osteogenesis imperfecta type I-IV. *Hum Mutat* 2006;27:716.

31. Kidszun A, Linebarger J, Walter JK, et al. What If the Prenatal Diagnosis of a Lethal Anomaly Turns Out to Be Wrong? *Pediatrics* 2016;137

32. Spencer RN, Hecher K, Norman G, et al. Development of standard definitions and grading for Maternal and Fetal Adverse Event Terminology. *Prenat Diagn* 2022;42:15-26.

33. Rapoport M, Bober MB, Raggio C, et al. The patient clinical journey and socioeconomic impact of osteogenesis imperfecta: a systematic scoping review. *Orphanet J Rare Dis* 2023;18:34.

34. Kolovos S, Javaid MK, Pinedo-Villanueva R. Hospital admissions of patients with osteogenesis imperfecta in the English NHS. *Osteoporos Int* 2021;32:1207-16.

35. Darba J, Marsa A. Hospital incidence, management and direct cost of osteogenesis imperfecta in Spain: a retrospective database analysis. *J Med Econ* 2020;23:1435-40.

36. Johansson M, Hermeren G, Sahlin NE. Paternal consent in prenatal research: ethical aspects. *Med Health Care Philos* 2020;23:325-31.

For peer review only

For peer review only - http://bmjopen.bmj.com/site/about/guidelines.xhtml

Table 1. Inclusion and Exclusion criteria for subjects in the BOOSTB4 trial.

For inclusion, all criteria should apply. For exclusion, one is enough to exclude.

Prenatal group: After birth and before the 2nd dose, subjects in the prenatal group will be assessed for the inclusion and exclusion criteria for the postnatal group.

INCLUSION CRITERIA		
**Postnatal group (n=15)**	**Prenatal group (n=15)**	**Control group (n=30–150 per subject)**
1. Parent's/legal guardian's signed informed-consent form,<br>2. Clinical diagnosis of OI type III or IV AND<br>3. Molecular diagnosis of OI (Glycine substitution in the collagen triple-helix encoding region of either the *COL1A1* or *COL1A2* gene),<br>4. Age less than 18 months (calculated from gestational week 40+0, i.e. the corrected age),<br>5. Parent/legal guardian over 18 years of age.	1. Woman has signed the patient consent form,<br>2. Only women where termination of the pregnancy is no longer possible or where the women are committed to continue the pregnancy may be included in the trial,<br>3. Suspicion of OI type III or IV in the fetus on ultrasound findings AND<br>4. Molecular diagnosis of OI in the fetus (Glycine substitution in the collagen triple-helix encoding region of either the *COL1A1* or *COL1A2* gene),<br>5. Gestation age between 16+0 and 35+6 weeks+days,<br>6. Pregnant women over 18 years of age.	*Matched historical controls:*<br>1. Parent's/legal guardian's signed informed-consent form,<br>2. Clinical and molecular diagnosis of OI (Glycine substitution in the collagen triple-helix encoding region of either the *COL1A1* or *COL1A2* gene),<br>3. Data on fractures and growth is available,<br>4. Parent/legal guardian over 18 years of age.<br><br>*Prospective untreated controls:*<br>- Postnatal participation: The inclusion criteria for the postnatal group apply.<br>- Prenatal participation: The inclusion criteria for the prenatal group apply, except inclusion criteria 2.

For peer review only

EXCLUSION CRITERIA		
**Postnatal group (n=15)**	**Prenatal group (n=15)**	**Control group (n=30–150)**
1. Existence of other known disorder that might interfere with the treatment, such as, but not limited to organ disfunction (for example liver or renal failure or bronchopulmonary dysplasia), congenital heart defect, hypoxic encephalopathy I-III, severe neurological problems, immune deficiencies, muscle diseases, severe malformations or syndromes diagnosed by clinical examination,<br>2. Any contraindication for invasive procedures such as a moderate/severe bleeding tendency,<br>3. Known risk factors for clotting, such as, but not limited to previous blood clot, family history of clots, clotting disorder (inherited or acquired), heart failure, inflammatory disorders (for example lupus, rheumatoid arthritis, inflammatory bowel disease),<br>4. Positive Donor Specific Antibody-test,<br>5. Known allergy/hypersensitivity to Fungizone and/or Gensumycin,<br>6. Abnormal karyotype or other confirmed genetic syndromes,<br>7. Oncologic disease (previous or current malignancy),<br>8. Inability to comply with the trial protocol and follow-up schedule,<br>9. Inability to understand the information and to provide informed consent.	1. Multiple pregnancy,<br>2. Co-existence of other disorder that might interfere with the treatment, as judged by the Investigator or the patient's obstetrician,<br>3. Abnormal fetal karyotype or other confirmed genetic syndrome,<br>4. Any contraindication for invasive procedures such as a bleeding tendency or contagious infections, such as, but not limited to HIV, Syphilis, Hepatitis B, Hepatitis C or other known infectious diseases that can harm the fetus,<br>5. Known risk factors for clotting, such as, but not limited to previous blood clot, family history of clots, clotting disorder (inherited or acquired), heart failure, inflammatory disorders (for example lupus, rheumatoid arthritis, inflammatory bowel disease),<br>6. Positive Donor Specific Antibody-test,<br>7. Known allergy/hypersensitivity to Fungizone and/or Gensumycin,<br>8. Oncologic disease in woman or fetus (previous or current malignancy),<br>9. Unwilling to or cannot undergo delivery by Caesarean section,<br>10. Inability to comply with the trial protocol and follow-up schedule,<br>11. Inability to understand the information and to provide informed consent.	*Matched historical controls (15/subject):*<br>1. Existence of other disorder that might interfere with the trial. No lung hypoplasia (type II OI),<br>2. Abnormal karyotype.<br><br>*Prospective untreated controls (n=up to 30):*<br>- Postnatal participation: The exclusion criteria, except exclusion criterium 4, 5, 6 and 7 for the postnatal group apply.<br>- Prenatal participation: The exclusion criteria, except exclusion criterium 1, 4, 5, 6 and 7 for the prenatal group apply.

For peer review only - http://bmjopen.bmj.com/site/about/guidelines.xhtml

BMJ Open

An exploratory open label multicentre phase I/II trial evaluating the safety and efficacy of postnatal or pre- and postnatal administration of allogeneic expanded fetal mesenchymal stem cells for the treatment of severe Osteogenesis Imperfecta in infants and fetuses: The BOOSTB4 Trial Protocol

Journal:	*BMJ Open*
Manuscript ID	bmjopen-2023-079767.R1
Article Type:	Protocol
Date Submitted by the Author:	03-Jan-2024

Complete List of Authors:	Sagar, Rachel; University College London, Elizabeth Garrett Anderson Institute for Women's Health ; NIHR University College London Hospitals Biomedical Research Centre<br>Åström, Eva; Karolinska Institutet, Department of Women's and Children Health; Karolinska University Hospital, Astrid Lindgren Children's Hospital<br>Chitty, Lyn; Great Ormond Street Hospital for Children NHS Foundation Trust, North Thames Genomic Laboratory Hub; UCL Great Ormond Street Institute of Child Health, Genetics and Genomics<br>Crowe, Belinda; Great Ormond Street Hospital for Children NHS Foundation Trust, Department of Neurosciences<br>David, Anna; University College London, Elizabeth Garrett Anderson Institute for Women's Health; NIHR University College London Hospitals Biomedical Research Centre<br>DeVile, Catherine; Great Ormond Street Hospital for Children NHS Foundation Trust, Department of Neurosciences<br>Forsmark, Annabelle ; PharmaLex<br>Franzen, Vera; XNK Therapeutics AB<br>Hermeren, Göran; Lund University Faculty of Medicine, Department of Clinical Sciences<br>Hill, Melissa; Great Ormond Street Hospital For Children NHS Foundation Trust, North Thames Genomic Laboratory Hub; UCL Great Ormond Street Institute of Child Health, Genetics and Genomics<br>johansson, mats; Lund University Faculty of Medicine, Department of Clinical Sciences<br>Lindemans, Caroline; University Medical Centre Utrecht, Department of Pediatrics,<br>Lindgren, Peter; Karolinska University Hospital, Center for Fetal Medicine; Karolinska Institutet, Department of Clinical Science, Intervention and Technology<br>Nijhuis, Wouter; University Medical Centre Utrecht, Department of Orthopedic Surgery<br>Oepkes, Dick; Leiden University Medical Center, Department of Obstetrics<br>Rehberg, Mirko; University Hospital Cologne, Department of Pediatrics<br>Sahlin, Nils-Eric; Lund University Faculty of Medicine, Department of Clinical Sciences

	Sakkers, Ralph; University Medical Centre Utrecht, Department of Orthopedic Surgery<br>Semler, O.; University Hospital Cologne, Department of Pediatrics<br>Sundin, Mikael; Karolinska Institutet, Department of Clinical Science, Intervention and Technology; Karolinska University Hospital, Section of Pediatic Hematology, Immunology and HCT<br>Walther-Jallow, Lilian; Karolinska Institutet, Department of Clinical Science, Intervention and Technology<br>Verweij, E.J.T; Leiden University Medical Center, Department of Obstetrics<br>Westgren, Magnus; Karolinska Institute, Department of Clinical Science, Intervention and Technology<br>Götherström, Cecilia; Karolinska Institute, Department of Clinical Science, Intervention and Technology
**Primary Subject Heading**:	Paediatrics
Secondary Subject Heading:	Obstetrics and gynaecology, Research methods
Keywords:	Mesenchymal Stem Cells, Cell biology < NATURAL SCIENCE DISCIPLINES, Fetal medicine < OBSTETRICS, Musculoskeletal disorders < ORTHOPAEDIC & TRAUMA SURGERY, Clinical trials < THERAPEUTICS, TRANSPLANT MEDICINE

For peer review only - http://bmjopen.bmj.com/site/about/guidelines.xhtml

An exploratory open label multicentre phase I/II trial evaluating the safety and efficacy of postnatal or pre- and postnatal administration of allogeneic expanded fetal mesenchymal stem cells for the treatment of severe Osteogenesis Imperfecta in infants and fetuses: The BOOSTB4 Trial Protocol

Rachel L Sagar (rachelsagar@nhs.net),[1,2]* Eva Åström (eva.astrom@regionstockholm.se),[3,4]* Lyn S Chitty (l.chitty@ucl.ac.uk),[5,6] Belinda Crowe (Belinda.Crowe@gosh.nhs.uk),[7] Anna L David (a.david@ucl.ac.uk),[1,2] Catherine DeVile (Catherine.DeVile@gosh.nhs.uk),[7] Annabelle Forsmark (annabelle.forsmark@pharmalex.com),[8] Vera Franzén (vera.frz@telia.com),[9] Göran Hermerén (goran.hermeren@med.lu.se),[10] Melissa Hill (melissa.hill@ucl.ac.uk),[5,6] Mats Johansson (mats.johansson@med.lu.se),[10] Caroline A Lindemans (C.A.Lindemans@prinsesmaximacentrum.nl),[11] Peter Lindgren (peter.lindgren.1@ki.se),[12,18] Wouter H Nijhuis (W.H.Nijhuis-2@umcutrecht.nl),[13] Dick Oepkes (D.Oepkes@lumc.nl),[14] Mirko Rehberg (mirko.rehberg@uk-koeln.de),[15] Nils-Eric Sahlin (nils-eric.sahlin@med.lu.se),[10] Ralph Sakkers (Sakkers@umcutrecht.nl),[13] Oliver Semler (joerg.semler@uk-koeln.de),[15] Mikael Sundin (mikael.sundin@ki.se),[16,17] Lilian Walther-Jallow (lilian.walther.jallow@ki.se),[18] E.J.T. (Joanne) Verweij (e.j.t.verweij@lumc.nl),[14] Magnus Westgren (magnus.westgren@ki.se)[18] and Cecilia Götherström (cecilia.gotherstrom@ki.se)[18]

*Shared first authorship.

[1]Elizabeth Garrett Anderson Institute for Women's Health, University College London, London, United Kingdom.

[2]NIHR University College London Hospitals Biomedical Research Centre, London, United Kingdom.

[3]Neuropediatric Unit, Astrid Lindgren Children's Hospital, Karolinska University Hospital, Stockholm, Sweden.

[4]Neuropediatric Unit, Department of Women's and Children Health, Karolinska Institutet, Stockholm, Sweden.

[5]North Thames Genomic Laboratory Hub, Great Ormond Street NHS Foundation Trust, London, United Kingdom.

[6]Genetics and Genomics, UCL Great Ormond Street Institute of Child Health, London, United Kingdom.

[7]Department of Neurosciences, Great Ormond Street Hospital for Children NHS Foundation Trust, London, United Kingdom.

[8]PharmaLex, Gothenburg, Sweden.

For peer review only - http://bmjopen.bmj.com/site/about/guidelines.xhtml

[9]XNK Therapeutics AB, Huddinge, Sweden.

[10]Medical Ethics, Department of Clinical Sciences, Lund, Faculty of Medicine, Lund University, Lund, Sweden.

[11]Department of Pediatrics, University Medical Center Utrecht, Utrecht, The Netherlands.

[12]Center for Fetal Medicine, Karolinska University Hospital, Stockholm, Sweden.

[13]Department of Orthopedic Surgery, University Medical Centre Utrecht, Wilhelmina Children's Hospital, Utrecht, The Netherlands.

[14]Department of Obstetrics, Division of Fetal Therapy, Leiden University Medical Center, Leiden, The Netherlands.

[15]Department of Pediatrics, Faculty of Medicine, University of Cologne and University Hospital Cologne, Cologne, Germany.

[16]Division of Pediatrics, Department of Clinical Science, Intervention and Technology, Karolinska Institutet, Stockholm, Sweden.

[17]Section of Pediatic Hematology, Immunology and HCT, Astrid Lindgren Children's Hospital, Karolinska University Hospital, Stockholm, Sweden.

[18]Division of Obstetrics and Gynecology, Department of Clinical Science, Intervention and Technology, Karolinska Institutet, Stockholm, Sweden.

Corresponding author and Sponsor's contact information: Cecilia Götherström, Division of Obstetrics and Gynecology, Department of Clinical Science, Intervention and Technology, Karolinska Institutet, ANA Futura, Alfred Nobels Allé 8, 141 52 Huddinge, Sweden. Phone +4670-4712300

Keywords: Osteogenesis Imperfecta; Brittle Bone Disease; Mesenchymal Stem Cells; Transplantation; Clinical Trial.

Word count: 4688 (not including the title page, abstract, references, figures, tables or acknowledgments.)

For peer review only

For peer review only - http://bmjopen.bmj.com/site/about/guidelines.xhtml

ABSTRACT

Introduction: Severe Osteogenesis Imperfecta (OI) is a debilitating disease with no cure or sufficiently effective treatment. Mesenchymal stem cells (MSCs) have good safety profile, show promising effects and can form bone. The Boost Brittle Bones Before Birth (BOOSTB4) trial evaluates administration of allogeneic expanded human first trimester fetal liver MSCs (BOOST cells) for OI type 3 or severe type 4.

Methods and analysis: BOOSTB4 is an exploratory, open label, multiple dose, phase I/II clinical trial evaluating safety and efficacy of postnatal (n=15) or prenatal and postnatal (n=3) administration of BOOST cells for the treatment of severe OI compared with a combination of historical (1–5 per subject) and untreated prospective controls (≤30). Infants <18 months of age and singleton pregnant women whose fetus has severe OI with confirmed glycine substitution in *COL1A1* or *COL1A2* can be included in the trial.

Each subject receives four intravenous doses of $3x10^6$/kg BOOST cells at 4-month intervals, with 48 (dose 1–2) or 24 (dose 3–4) hours in-patient follow-up, primary follow-up at 6 and 12 months after the last dose, and long-term follow-up yearly until 10 years after the first dose. Prenatal subjects receive the 1st dose via ultrasound-guided injection into the umbilical vein within the fetal liver (16+0 to 35+6 weeks), and three doses postnatally.

The primary outcome measures are safety and tolerability of repeated BOOST cell administration. The secondary outcome measures are number of fractures from baseline to primary and long-term follow-up, growth, change in bone mineral density, clinical OI status and biochemical bone turnover.

Ethics and dissemination: The trial is approved by competent authorities in Sweden, the United Kingdom and the Netherlands (postnatal only). Results from the trial will be disseminated on EudraCT and ClinicalTrials.gov and published in scientific open access scientific journals.

Trial registration numbers: EudraCT (2015-003699-60), ClinicalTrials.gov (NCT03706482); Pre-results.

For peer review only - http://bmjopen.bmj.com/site/about/guidelines.xhtml

Strengths and limitations of this study:

- This is the first clinical trial to assess whether postnatal or pre- and postnatal repeated intravenous administration of BOOST cells is a safe and potentially effective treatment option for severe OI.
- This is a protocol for an open label clinical trial; which means that there is no randomisation and the treatment is known both to the families and the trial personnel.
- OI is by nature a heterogenous and variable disease, which may impact the possibility to satisfactorily evaluate the effect of treatments. To address this and collect reliable data, the BOOSTB4 trial collects data longitudinally and includes multiple follow-up assessments of each subject.
- The trial may be underpowered to show improvements in efficacy. Therefore, if the trial yields a positive result, a larger subsequent multi-centre pivotal trial may be needed.
- MSCs are currently the only known therapy tested clinically for OI that addresses the underlying pathology with the aim of producing healthy bone cells and healthy collagen. However, the clinical effects of MSC administration may not be persistent and therefore re-dosing may be required. The interval for re-dosing is currently not known.

For peer review only

For peer review only - http://bmjopen.bmj.com/site/about/guidelines.xhtml

INTRODUCTION

Osteogenesis imperfecta (OI) is a rare clinically and genetically heterogenous group of heritable disorders of connective tissue characterised by osteopenia, growth restriction, bone deformities, fractures and chronic pain.(1) Originally, OI was classified into four types according to clinical, radiological and histological findings, where type 1 refers to the mildest form, type 2 to the perinatally lethal form, type 3 to the most severe form compatible with life, and type 4 to an intermediate group with moderate to severe types of OI.(2) This classification covers 85–90% of persons with OI and includes >1,500 different autosomal dominant sequence variations in the genes coding for type I collagen (*COL1A1* or *COL1A2*).(1-3) There is currently no curative treatment for OI, with the only available pharmaceutical treatments failing to address the underlying molecular pathology, the collagen defect.(4) Treatment is aimed at increasing overall bone strength to prevent fractures and maintain mobility. Bisphosphonates are used to reduce bone resorption and increase bone mass.(5) However, two meta-analyses of randomised trials on bisphosphonate treatment for OI did not demonstrate improvement in fracture rates, reduction of pain or improved functional mobility, although multiple studies report this independently.(6 7)

One child among 10–20,000 is born with OI. Diagnosis of severe types of OI is often made at the routine fetal anomaly ultrasound scan mid-pregnancy, or early if first trimester scanning is routine. Early treatment of OI is preferred to minimise irreversible pathology. Prenatal intervention offers multiple potential advantages over postnatal approaches; ameliorating and preventing damage that has begun already during fetal life, the relatively naive fetal immune system may permit the development of immune tolerance towards donor cells, infusion into the systemic fetal circulation may avoid sequestration of the cells in the lungs because circulatory shunts allow the pulmonary circulation to be bypassed, fetal life is a time of natural stem cell proliferation and migration to different anatomic compartments. Also, prenatal administration may enhance the effect of MSCs because there will be a lesser amount of poor quality OI type collagen that requires replacement, and

For peer review only - http://bmjopen.bmj.com/site/about/guidelines.xhtml

lastly, the possible psychological benefits for parents of commencing treatment as early in the child's life.(8)

The investigational medicinal product (IMP) investigated in the BOOSTB4 trial is an advanced therapy medicinal product (ATMP) consisting of allogeneic *ex vivo* expanded human fetal liver-derived mesenchymal stem cells (MSCs) from the first trimester of pregnancy. MSCs are multipotent stem cells and are promising candidates in regenerative medicine due to their low immunogenic profile that allows the possibility of performing cell transplantation across major histocompatibility barriers without immunosuppression.(9 10) In addition, they have the potential to differentiate along the osteogenic, chondrogenic and adipogenic lineages and have a good safety profile with minimal oncogenic risk.(11-16) Two meta-analyses of eight and 55 randomised controlled trials investigating intravascular administration of naïve MSCs to 321 and 2696 subjects, respectively did not detect an association between non-fever acute infusional toxicity, organ system complications, thrombotic/embolic events, infection, death or malignancy.(17 18) The only significant association with intravascular administration of MSCs was a transient fever.(17 18) The first clinical infusion of MSCs was performed over 20 years ago and adult MSCs have since been used in a diverse range of conditions in thousands of patients.(9 17) Studies show that MSCs derived from developmentally younger donor sources present characteristics suggesting a stronger cell therapeutic potential than adult MSCs, and this knowledge is being translated into clinical application. First trimester fetal MSCs are found at a higher frequency, are less mature on the transcriptomic level, have a greater colony-forming and proliferation capacity and are more immune modulatory compared to adult MSCs.(10 19-22) Interestingly, fetal MSCs differentiate into more lineages than MSCs derived from adult tissues, like skeletal muscle, oligodendrocytes and hepatic cells,(23 24) (25) and differentiate more readily into bone than adult MSCs. In a direct comparison of fetal MSCs and MSC derived from Wharton's Jelly, adult bone marrow and adipose tissue, fetal MSCs had higher levels of osteogenic genes under basal conditions. (26 27) Upon osteogenic differentiation, fetal MSCs produced more robust osteogenic gene expression, induced more calcium deposition *in vitro* and reached higher levels of osteogenic

For peer review only - http://bmjopen.bmj.com/site/about/guidelines.xhtml
For peer review only

gene upregulation *in vitro* and *in vivo*.(26-28) First trimester fetal liver MSC were selected for use in this trial given their superior osteogenic differentiation capacity, their safety profile and the feasibility of manufacturing a high quality GMP-compliant off-the-shelf product with retained efficacy.

The potential of MSC transplantation for the treatment of OI has been shown in different mouse models of OI, with demonstrated homing and engraftment in bone, increased collagen content and mineralisation of the bones, improved bone strength, thickness and length, and 67–84% reduction in long bone fractures.(29-31) Clinically, two studies on intravenous administration of histocompatible MSCs from sibling donors to children with severe OI have been performed (6 and 2 children treated respectively, where 6 children underwent myeloablative haematopoietic stem cell transplantation before they received MSCs(32 33)).(34 35) The MSC administration procedures were safe with no serious adverse events (SAEs) reported, and positive clinical effects were attained with donor cell engraftment, development of new compact bone, improved bone parameters, and six months following two MSC infusions there was an increase in growth velocity, an 80% reduction in fracture frequency, an increase in total bone mineral content in 3/8 of the children(34 35) and in Quality of Life.(35 36)

We have previously reported two case studies of prenatal and postnatal transplantation of fetal MSCs for severe OI (type 3 and type 4 OI).(37 38) The limited data show that the procedures were safe and clinically promising when comparing the clinical course to other individuals with identical OI sequence variants. Only three other individuals in the world are currently known to have a *COL1A2* variant identical to that of the patient with OI type 3; one of whom died from the severe type of OI despite bisphosphonate treatment, and two individuals are described as having type 2/3 OI.(39 40) After one dose of fetal MSCs prenatally, donor cells were found in the recipient bones at nine months of age, and the donor cells expressed bone markers.(37) Four booster doses with same donor MSCs between 8–13 years of age show a reduction in fracture rate (from 2/year to 0/year over two years), and an increase in height, albeit 5.0–6.4 standard deviations below the mean.(38) While it is difficult to

For peer review only - http://bmjopen.bmj.com/site/about/guidelines.xhtml

determine the definitive impact of the prenatal and postnatal MSC transplantation in these two heterogeneous cases, the findings suggest a potential clinical benefit. The children have now been followed up for over 21 and 14 years, respectively, and there has been no evidence of any early or late adverse reactions.

Donor cell engraftment in bones has been reported to be between 0 to 16.4% after stem cell transplantation in both human cases of OI and mouse models.(29-34 37 38) It is difficult to determine if such low levels of engraftment could result in adequate synthesis of healthy type I collagen and significant clinical improvement. However, Panaroni et al. reported that *in utero* transplantation of healthy bone marrow to the Brtl OI mouse resulted in donor cell engraftment of 1–2% in the bones, which in turn resulted in a 20% production of healthy collagen in the host bones.(30) The transplantation eliminated the perinatal lethality of Brtl mice, and furthermore the femora of treated Brtl mice had significant improvement in geometric parameters compared to Brtl mice and in mechanical properties compared to wild-type mice. Hence, low level engraftment of cells producing healthy collagen may lead to significant improvements in bone development.

Transplantation of MSCs is not a cure, but it is currently the only therapy available in clinical phase trials for OI that may result in healthy bone cells and production of healthy collagen. Other treatments developed for OI are based on modifying the action of the patient's own cells, and hence can only result in increased quantity of bone rather than improved quality.

There is increasing interest in treatment with MSCs and a growing number of requests from patients and their physicians to provide it. In the absence of a clinical trial, prenatal and postnatal MSC transplantation for OI is likely to be practised around the world on a case-by-case basis, making it impossible to evaluate any effects the intervention might have. Considering the complexity of this field and the distribution of cases, an international trial is the only realistic and ethically correct approach to enable accurate evaluation of the intervention. The BOOSTB4 trial will provide this well-defined framework for assessment of children and fetuses with OI treated with fetal MSCs.

For peer review only - http://bmjopen.bmj.com/site/about/guidelines.xhtml

METHODS AND ANALYSIS

Trial design

BOOST Brittle Bones Before Birth (BOOSTB4) is an exploratory, open label, multicentre, phase I/II trial evaluating safety and efficacy of four postnatal intravenous doses (n=15 infants) or one prenatal and three postnatal intravenous doses (n=3 fetuses, previously n=15) of allogeneic expanded fetal MSCs (BOOST cells) for the treatment of severe OI compared with a combination of historical (1–5/subject) and up to 30 untreated prospective controls. Please see the trial synopsis in Supplementary material.

Setting

The Sponsor for this academic trial is Karolinska Institutet (coordinator) in Sweden. The trial sites are five maternity/fetal medicine and paediatric institutions; Karolinska University Hospital in Sweden (coordinating trial centre, OI and maternal/fetal medicine, manufacturer of the IMP), University College London (trial centre, maternal/fetal medicine) and Great Ormond Street Hospital for Children (trial centre, OI) in the United Kingdom, and Leiden University Medical Center (trial centre, maternal/fetal medicine) and University Medical Center Utrecht (trial centre, OI) in the Netherlands. The trial has ethical and regulatory approval from the National Competent Authorities in Sweden (the Swedish Ethical Review Authority 2018/1732-31, the Swedish Medical Product Agency 5.1-2018-57411), United Kingdom (the National Ethics committee in York 19/NE/0030, the UK Medicines & Healthcare products Regulatory Agency 44119/0002/001-001), Netherlands (the Central Committee on Research Involving Human Subjects NL64105.000-17, postnatal part) and NHS Research Ethics Committee approval (16/NS/0084) for the interview study in the United Kingdom. Amendments may be implemented after approval from the National Competent Authorities.

The trial is performed according to Good Clinical Practice (GCP). The data is entered into electronic Case Report Forms, and the data and trial are monitored according to the trial's Monitoring Plan. The conduct of the trial is overseen by an independent Data and Safety Monitoring Committee (DSMB)

For peer review only - http://bmjopen.bmj.com/site/about/guidelines.xhtml

according to the DSMB Charter. The DSMB includes expertise on OI, haematology, transplantation and fetal and maternal medicine.

Investigational Medicinal Product

The IMP consists of cryopreserved expanded human first trimester liver-derived MSCs (BOOST cells), obtained from early elective surgical terminations of pregnancy. The Mechanism of Action of the IMP is engraftment and bone differentiation. The IMP is manufactured under Good Manufacturing Practice (GMP) and is tested with regards to safety and functionality according to the approved product specification. At least 80 clinical doses of MSCs, and MSCs for retention samples and all analyses will be manufactured from two different donors used throughout the BOOSTB4 trial.

Eligibility and recruitment

The inclusion and exclusion criteria for the three trial groups are described in Table 1. All participants (the postnatal, prenatal and control groups) will receive bisphosphonate treatment. At least one dose of bisphosphonate must be administered before the first dose of stem cells in the postnatal group. In the prenatal group the first dose of bisphosphonates will be administered after birth. Acceptable types of concomitant medication including bisphosphates during the trial and until the 12-month follow-up are Pamidronate (preferred) or Neridronate, and the annual dose of bisphosphate is 8-12 mg/kg body weight per year.

Information about the BOOSTB4 trial has been distributed to specialist OI services and fetal medicine units in Europe, and the BOOSTB4 trial team can also be contacted directly by families or physicians via email or the trial specific website boostb4.eu where the Patient Information Leaflet is available in several languages. The potential patients are invited to visit the trial site or have an organized video meeting and provide their oral and written consent to be screened for participation. At least three days will be required for the parents to consider participation in the trial. For all children enrolled in the trial, a signed informed-consent form for the main trial is required from the mother and the father, if she/he has parental responsibility (see Supplementary material for the screening and main trial

For peer review only - http://bmjopen.bmj.com/site/about/guidelines.xhtml

participation consent forms for the postnatal and prenatal groups, and www.boostb4.eu for the Participant information sheets). For prenatal inclusion, only the pregnant woman needs to sign the informed-consent form, but both parents are encouraged to sign. After the birth of a child in the prenatal group the inclusion and exclusion criteria for the postnatal group must be fulfilled (Table 1), and both parents must re-consent their participation in the trial before the first postnatal dose. Consent may be withdrawn at any time without a reason being stated. The Sponsor will hold clinical trial insurance that covers all enrolled subjects receiving active treatment.

Sample size calculation

No formal power calculations have been performed. OI is a rare disease, and a limited number of subjects can be included each year. Based on the incidence, it is reasonable to include 30 patients (15 postnatal subjects and 15 prenatal subjects) in this first trial on the grounds of feasibility, suitable time period and for the application of descriptive statistical analyses.

Blinding, intervention and follow-up

It would not be ethically appropriate to perform a randomised double-blind placebo-control trial in this population, particularly regarding the risks of the prenatal infusion procedure and the vulnerability of the subjects. No blinding or randomisation will therefore take place.

Four intravenous doses of BOOST cells are administered at four-month intervals (range 3–5 months), please see Figure 1 for an overview of the trial. For postnatal subjects the 1st dose must be administered before 18 months of age. For prenatal subjects, the 1st dose will be given *in utero* during gestational weeks+days 16+0–35+6 via ultrasound-guided injection into the umbilical vein within the fetal liver, and the final three doses are administered postnatally. Each dose contains $3x10^6$ allogeneic expanded human fetal liver MSCs per kg bodyweight. All doses to a subject will be derived from one donor. No pre-conditioning is administered.

For peer review only - http://bmjopen.bmj.com/site/about/guidelines.xhtml

In-patient follow-up is performed over 48 (dose 1–2) or 24 (dose 3–4) hours in the postnatal group and over 48 (dose 1–3) or 24 (dose 4) hours in the prenatal group after each dose. Primary follow-up is performed at 6 and 12 months after the last dose, and long-term follow up is performed yearly until 10 years after the first dose. At each visit and follow-up, AEs and parameters related to efficacy outcomes are recorded. Women in the prenatal group are followed under the same schedule.

Prospective controls will be subject to the same follow-up routine as the patients enrolled in the treatment group, as described above, with the option to opt in or out from blood collection. The clinical evaluation program for the historical control group will be based on data available from the routine follow-up of OI patients in the respective country. The controls will be individually matched to each subject (see Table 2). Between one and five historical controls will be included per subject and up to 30 prospective controls in total.

For peer review only

Outcome measures

The primary endpoint of the BOOSTB4 trial is safety and tolerability measured as seriousness, severity and frequency of administration-related adverse events (AEs) in the child, fetus and woman after four postnatal or one prenatal and three postnatal intravenous doses of BOOST cells every four months. Specific focus will be given to vital signs, transfusion and immune reactions and maternal and fetal AEs using the Maternal and Fetal Adverse Event Terminology,(41) for example, persistent fetal bradycardia following the infusion, tumorigenicity, morbidity and mortality. The trial has clearly defined Stopping criteria on trial, group and subject levels. In case of a Serious Adverse Reaction, the subject may not receive more doses of BOOST cells. The DSMB will review all Adverse Reactions.

Vital signs (oxygen saturation, heart rate, respiratory rate, body temperature, blood pressure [only in the pregnant woman], medical examination and signs of suspected pulmonary embolism) are monitored in the child and pregnant woman before, during and until 48 hours (dose 1–2) or 24 hours (dose 3–4) after each dose. In the fetus the following is monitored before, during and until 48 hours

For peer review only - http://bmjopen.bmj.com/site/about/guidelines.xhtml

after the prenatal dose using ultrasound: fetal heart rate, middle cerebral artery pulsatility index, peak systolic velocity, umbilical artery Doppler pulsatility index and cardiotocography.

The following parameters are analysed in peripheral blood before, and at 1, 24 (dose 1–4) and 48 hours (dose 1–2) after each dose (does not apply to the fetus): Blood status (Haemoglobin, Haematocrit, Leukocytes, Thrombocytes, Erythrocytes, Mean corpuscular volume), liver (ALAT and ASAT), kidney (Creatinine), Electrolytes (Chloride, Magnesium, Sodium, Potassium). Donor specific antibodies are analysed before the first dose and >3 weeks after each dose.

The secondary endpoints relate to efficacy of four doses of BOOST cells in children with OI. The hypothesis under investigation is that early administration of BOOST cells will ameliorate severe types of OI. The key secondary endpoint is number of fractures from baseline to primary (12 months after the last dose) and long-term (10 years after the first dose) follow-up. Fracture frequency was chosen as the key secondary endpoint because it is the most clinically relevant efficacy variable. Additional secondary endpoints are time to first fracture after each BOOST cell administration, number of fractures at birth in the prenatal group, growth (length/height and weight), change in bone mineral density measured with DXA/DEXA (optional and according to the national follow-up program), clinical OI status and biochemical bone turnover in peripheral blood. The analysis of biochemical bone turnover includes Calcium, Phosphate, Albumin, Parathyroid hormone, 25-OH Vitamin D, Alkaline phosphatase, Bone specific Alkaline phosphatase, Osteocalcin and cross-linked C-telopeptide of type I collagen, and will be carried out before each dose and then according to the clinical routine every 6–12 months. No extra radiological measurements will be performed specifically for the trial.

The exploratory endpoints investigated by the trial include assessing the impact of the therapy on Quality of Life using the Infant Toddler Quality of Life Questionnaire™ (ITQOL), collection of peripheral blood before and at 1 and 24h (dose 1–4) and 48h (dose 1–2) after each dose for studying the paracrine effect (via the Olink technology or ELISA) and the effect on endogenous immune cells (via flow cytometry) of BOOST cells in subjects and prospective controls (blood sampling optional). The

For peer review only - http://bmjopen.bmj.com/site/about/guidelines.xhtml

extent of donor cell engraftment in tissue samples collected from routine surgery will be studied, and a method for non-invasive method for prenatal diagnosis of OI developed (not used as the diagnostic method in the trial). In addition, there will be a sub-study to BOOSTB4, where parents will have the option to take part in two semi-structured interviews, in which their experiences of their participation in the trial are explored. This will allow us to understand their reasoning for their child to participate in the trial, and what has been positive and what can be improved in future similar studies.

Statistical analysis

The primary endpoint of the BOOSTB4 trial is the safety of postnatal or prenatal and postnatal administration of BOOST cells. It involves investigation of the seriousness, severity and frequency of treatment-related AEs in the child, woman and fetus. All reported AEs (regardless of the cause) will be included in the statistical analysis, and all analyses will be performed separately for the infant/child, fetus and pregnant woman. No imputation of missing data will take place.

The BOOSTB4 trial is a non-randomised trial and specific caution will be taken to control for problems with bias. The statistical analysis will include descriptive statistics of the data that will be discussed in relation to the general knowledge of the condition, to generate an overall picture of the safety and efficacy profile of the treatment. All trial subjects will be compared to himself or herself as his or her own control over time (longitudinally from baseline to the primary follow-up at 12 months after the last dose and to the long-term follow-up until 10 years after the first dose). In the analysis of parameters such as blood status and biochemical bone turnover, non-parametric Wilcoxon matched pairs signed-rank test (before versus after doses and over time) of will be used. One to five matched historical controls (30–150 in total) will, as far as possible, be included per trial subject to increase the statistical precision, and up to 30 prospective untreated controls will also be included.

For peer review only - http://bmjopen.bmj.com/site/about/guidelines.xhtml

For peer review only

Patient and public involvement

The trial has been discussed with patient support groups including the OI Federation Europe (OIFE) and Care 4 Brittle Bones (C4BB) from early in the planning stage. We are grateful for their input, and for the joint dissemination of the trial in 2019.

In a separate study we explored stakeholder views to understand perceived benefits or concerns, identify ethical issues and establish protocols for support and counselling. Semi-structured qualitative interviews were conducted with 56 participants from three groups: adults affected with OI, with and without children, and parents of children affected with OI, health professionals who work with patients with OI, and patient advocates from relevant patient support groups.(8) There were generally positive views towards using fetal MSC transplantation to treat OI, with early treatment considered to be advantageous for preventing fractures and reducing severity, as well as potentially bringing psychological benefits for parents. Participants were somewhat concerned about procedure safety, short and long-term side effects and the effectiveness of transplantation. The difficulties inherent in decision-making were frequently raised, as well as the importance of explaining fetal MSC transplantation in a way that all parents can understand, setting clear expectations, providing psychological support and allowing time to reflect during the decision-making process. The feedback received has been incorporated into the design and planning of the BOOSTB4 trial.

Health economic assessment

A health economic assessment is being undertaken with the objective to describe the costs and effects of BOOST cell administration as a potential standard treatment for severe forms of OI. Published data on the impact of OI on healthcare burden are scarce,(42) with available studies reporting a high cost for e.g. frequent hospital admissions.(43 44) Also, two sets of questionnaires have been developed to capture dependency on healthcare and/or community services, with one focusing on patient/family (postnatal and prenatal groups) and the other on maternal health (prenatal group). These

For peer review only - http://bmjopen.bmj.com/site/about/guidelines.xhtml

questionnaires will be used to further assess the costs and effects of BOOST cell transplantation for severe forms of OI, in addition to further exploring the economic burden of OI overall.

Trial status

Recruitment commenced in Sweden after the site opened on 12th of August 2019. The first subject in the postnatal group was included and received the 1st dose on 17th of March 2020. Due to the SARS-CoV-2 pandemic only two subjects were included in the trial during 2020, and due to time restrictions for the project, the dose administrations are only performed at Karolinska University Hospital in Sweden. After a safety assessment by the Data and Safety Monitoring Board of at least one dose administered to the 1st five postnatal subjects in the trial, the prenatal group of the trial was opened 17th of June 2021, and the 1st prenatal dose was administered on 18th of November 2021. By 2nd of March 2022, all 15 of 15 subjects in the postnatal group had been included and received their 1st dose, and by 14th of December 2021, three of 15 prenatal subjects had been included and administered with one prenatal dose each. No more subjects will be included in the prenatal group due to time restrictions in funding of the trial. The primary follow-up is currently being performed and is expected to be completed in January 2024, and the long-term follow-up will be completed in 2032.

ETHICS

The BOOSTB4 clinical trial aims to investigate fetal-derived stem cell treatment in young children and unborn children (which includes the pregnant woman as a research subject) suffering from a chronic disease. Several ethical issues arise when considering stem cell transplantation for OI in these study populations. Therefore, a detailed and systematic risk – benefit assessment was performed before initiation of the trial, which is updated when needed, for example during the COVID pandemic. The assessment considers many areas; risks related to handling, administration and exposure to the IMP, and risks for the child, fetus and pregnant woman. The assessment also includes potential benefits for the child, fetus and the parents. All risks and benefits are discussed in relation to the Sponsor's

For peer review only - http://bmjopen.bmj.com/site/about/guidelines.xhtml

experience with administration of fetal MSCs for treatment of OI. In other words, this therapy is believed to be directly beneficial to the fetus and child, but there are also significant unknowns.

In the BOOSTB4 study there have been several separate considerations of diverse ethical issues. They include reviewing the ethical and legal standards relevant to prenatal and postnatal stem cell transplantation, mapping the rights and interests of the research subjects, investigating stakeholder's views,(8) as well as testing the Patient Information Leaflet and the communication of risks.

Identifying the main stakeholders is fundamental since the safety and rights of the research subjects should be the main concern. At the prenatal stage the main stakeholders are the pregnant woman and the fetus. At the postnatal stage, only the minor is the research subject, but the parents are also stakeholders, since the infant cannot consent to the participation. We have also discussed the risk of overprotection of pregnant women since they are considered to be a vulnerable population. This may violate their autonomy, harm their interests, and prevent research that could help children and possibly improve their own Quality of Life. Overprotection may signal good intentions and an ambition to minimise risks, but it could also result from a tendency to underestimate the benefits the therapy may bring to the women from whom consent is being sought. Partner consent is not legally required prenatally, although there are exceptions (discussed in (45)). Nonetheless, it is recommended if the partner is expected to become the child's legal representative after birth. It will also be impossible to complete the trial if the partner dissents after birth, and the trial concerns the partner in many ways.

As a sub-study to BOOSTB4, the parents can consent to take part in two qualitative interviews. This will allow us to understand their reasoning for their child to participate in the trial, and what has been positive and what can be improved in future similar studies.

DISSEMINATION

Trial results will be uploaded on EudraCT and ClinicalTrials.gov and published in scientific open access journals when the primary follow-up has been completed, and also when and the long-term follow-

For peer review only - http://bmjopen.bmj.com/site/about/guidelines.xhtml

up has been completed. Lay summaries of the findings will be shared via the networks of the OI patient organisations OIFE and C4BB, and distributed to the families in the BOOSTB4 trial that have opted to receive such summaries. Details of the trial and progress will be shared by presentation at relevant national and international meetings.

Authors' contributions

CG, MW, EÅ, OS and ALD conceived the idea for the trial. All authors (RSL, EÅ, LSC, BL, ALD, CDV, AF, VF, GH, ML, MJ, CAL, PL, WHN, DO, MR, NES, RS, OS, MS, LWJ, EJTW, MW, CG) developed the trial protocol. CG, MW, EÅ, and LWJ wrote the initial trial protocol, which was subsequently revised by all authors. GH, MJ and NES wrote the initial texts on ethics and regulatory issues. EÅ developed the clinical practice of OI follow-up. MS developed the clinical practice and clinical safety parameters, and developed the study database. MH and LSC designed the research with stakeholders, MH conducted the research with stakeholders and contributed to the development of parent facing study materials. RS, EÅ and CG wrote the draft of the manuscript and all authors (RSL, EÅ, LSC, BL, ALD, CDV, AF, VF, GH, ML, MJ, CAL, PL, WHN, DO, MR, NES, RS, OS, MS, LWJ, EJTW, MW, CG) revised, reviewed and approved the final manuscript.

Funding statement

This project has received funding from the European Union's Horizon 2020 research and innovation programme under grant agreement No 681045, Vetenskapsrådet (The Swedish Research Council,921-2014-7209 and 2020-01666), Region Stockholm in Sweden (20200365, FoUI-975495), the Center for Innovative Medicine (CIMED) in Sweden (FoUI-962010), and the Foundation for Children of the Freemasons in Stockholm. Parts of this work were supported by grants from the German Science Foundation (Deutsche Forschungsgemeinschaft, DFG) to the Research Unit FOR 2722; Refnr SE2373/1-2, Project 384170921. LSC is partially funded by the NIHR Great Ormond Street Hospital (GOSH) Biomedical Research Centre (BRC). All research at Great Ormond Street Hospital NHS Foundation Trust

For peer review only - http://bmjopen.bmj.com/site/about/guidelines.xhtml
For peer review only

and UCL Great Ormond Street Institute of Child Health is made possible by the NIHR Great Ormond Street Hospital. ALD is partially funded by the NIHR University College London Hospitals BRC. The views expressed are those of the authors and not necessarily those of the NHS, the NIHR, or the UK Department of Health.

Competing interest statement

ALD is a consultant for Esperare Foundation for a clinical trial unrelated to this work. CG, LWJ and MW are co-founders and co-owners of BOOST Pharma ApS founded in 2020. OS is a scientific advisor for BOOST Pharma ApS. AF, BC, CAL, CDV, DO, EJTV, EÅ, GH, LSC, MH, MJ, MR, MS, NES, PL, RLS, RS, VF and WN declare no competing interests.

Data statement

Trial data is found at ClinicalTrials.gov ID NCT03706482. The Sponsor Karolinska Institutet and the Investigators will have access to the final trial dataset.

Acknowledgements

We thank all families taking part in the BOOSTB4 trial, and physicians collaborating in accomplishing the trial. We also thank OIFE and C4BB for their constructive and valuable support.

For peer review only - http://bmjopen.bmj.com/site/about/guidelines.xhtml

REFERENCES

1. Van Dijk FS, Sillence DO. Osteogenesis imperfecta: clinical diagnosis, nomenclature and severity assessment. *Am J Med Genet A* 2014;164A:1470-81.

2. Sillence DO, Senn A, Danks DM. Genetic heterogeneity in osteogenesis imperfecta. *J Med Genet* 1979;16:101-16.

3. Van Dijk FS, Pals G, Van Rijn RR, et al. Classification of Osteogenesis Imperfecta revisited. *Eur J Med Genet* 2010;53:1-5.

4. Thomas IH, DiMeglio LA. Advances in the Classification and Treatment of Osteogenesis Imperfecta. *Curr Osteoporos Rep* 2016;14:1-9.

5. Glorieux FH, Bishop NJ, Plotkin H, et al. Cyclic administration of pamidronate in children with severe osteogenesis imperfecta. *N Engl J Med* 1998;339:947-52.

6. Dwan K, Phillipi CA, Steiner RD, et al. Bisphosphonate therapy for osteogenesis imperfecta. *Cochrane Database Syst Rev* 2014;7:CD005088.

7. Hald JD, Evangelou E, Langdahl BL, et al. Bisphosphonates for the prevention of fractures in osteogenesis imperfecta: meta-analysis of placebo-controlled trials. *J Bone Miner Res* 2015;30:929-33.

8. Hill M, Lewis C, Riddington M, et al. Stakeholder views and attitudes towards prenatal and postnatal transplantation of fetal mesenchymal stem cells to treat Osteogenesis Imperfecta. *Eur J Hum Genet* 2019;27:1244-53.

9. Squillaro T, Peluso G, Galderisi U. Clinical Trials With Mesenchymal Stem Cells: An Update. *Cell Transplant* 2016;25:829-48.

10. Götherström C, Ringden O, Westgren M, et al. Immunomodulatory effects of human foetal liver-derived mesenchymal stem cells. *Bone Marrow Transplant* 2003;32:265-72.

11. Prockop DJ, Brenner M, Fibbe WE, et al. Defining the risks of mesenchymal stromal cell therapy. *Cytotherapy* 2010;12:576-8.

12. Pittenger MF, Mackay AM, Beck SC, et al. Multilineage potential of adult human mesenchymal stem cells. *Science* 1999;284:143-7.

13. Bernardo ME, Zaffaroni N, Novara F, et al. Human bone marrow derived mesenchymal stem cells do not undergo transformation after long-term in vitro culture and do not exhibit telomere maintenance mechanisms. *Cancer Res* 2007;67:9142-9.

14. Barkholt L, Flory E, Jekerle V, et al. Risk of tumorigenicity in mesenchymal stromal cell-based therapies –bridging scientific observations and regulatory viewpoints. *Cytotherapy* 2013;15:753-9.

15. von Bahr L, Batsis I, Moll G, et al. Analysis of tissues following mesenchymal stromal cell therapy in humans indicates limited long-term engraftment and no ectopic tissue formation. *Stem Cells* 2012;30:1575-8.

For peer review only - http://bmjopen.bmj.com/site/about/guidelines.xhtml

16. Rodriguez-Fuentes DE, Fernandez-Garza LE, Samia-Meza JA, et al. Mesenchymal Stem Cells Current Clinical Applications: A Systematic Review. *Arch Med Res* 2021;52:93-101.

17. Lalu MM, McIntyre L, Pugliese C, et al. Safety of cell therapy with mesenchymal stromal cells (SafeCell): a systematic review and meta-analysis of clinical trials. *PLoS One* 2012;7:e47559.

18. Thompson M, Mei SHJ, Wolfe D, et al. Cell therapy with intravascular administration of mesenchymal stromal cells continues to appear safe: An updated systematic review and meta-analysis. *EClinicalMedicine* 2020;19:100249.

19. Guillot PV, Gotherstrom C, Chan J, et al. Human first-trimester fetal MSC express pluripotency markers and grow faster and have longer telomeres than adult MSC. *Stem Cells* 2007;25:646-54.

20. Götherström C, West A, Liden J, et al. Difference in gene expression between human fetal liver and adult bone marrow mesenchymal stem cells. *Haematologica* 2005;90:1017-26.

21. Yu Y, Valderrama AV, Han Z, et al. Human fetal liver MSCs are more effective than adult bone marrow MSCs for their immunosuppressive, immunomodulatory, and Foxp3(+) T reg induction capacity. *Stem Cell Res Ther* 2021;12:138.

22. Götherström C, Ringden O, Tammik C, et al. Immunologic properties of human fetal mesenchymal stem cells. *Am J Obstet Gynecol* 2004;190:239-45.

23. Chan J, O'Donoghue K, Kennea N, et al. Myogenic potential of fetal mesenchymal stem cells. *Ann Acad Med Singapore* 2003;32:S11-3.

24. Kennea NL, Waddington SN, Chan J, et al. Differentiation of human fetal mesenchymal stem cells into cells with an oligodendrocyte phenotype. *Cell Cycle* 2009;8:1069-79.

25. Wei X, Wang CY, Liu QP, et al. In vitro hepatic differentiation of mesenchymal stem cells from human fetal bone marrow. *J Int Med Res* 2008;36:721-7.

26. Zhang ZY, Teoh SH, Chong MS, et al. Superior osteogenic capacity for bone tissue engineering of fetal compared with perinatal and adult mesenchymal stem cells. *Stem Cells* 2009;27:126-37.

27. Guillot PV, De Bari C, Dell'Accio F, et al. Comparative osteogenic transcription profiling of various fetal and adult mesenchymal stem cell sources. *Differentiation* 2008;76:946-57.

28. Zhang ZY, Teoh SH, Chong MS, et al. Neo-vascularization and bone formation mediated by fetal mesenchymal stem cell tissue-engineered bone grafts in critical-size femoral defects. *Biomaterials* 2010

29. Guillot PV, Abass O, Bassett JH, et al. Intrauterine transplantation of human fetal mesenchymal stem cells from first-trimester blood repairs bone and reduces fractures in osteogenesis imperfecta mice. *Blood* 2008;111:1717-25.

30. Panaroni C, Gioia R, Lupi A, et al. In utero transplantation of adult bone marrow decreases perinatal lethality and rescues the bone phenotype in the knockin murine model for classical, dominant osteogenesis imperfecta. *Blood* 2009;114:459-68.

For peer review only - http://bmjopen.bmj.com/site/about/guidelines.xhtml

31. Vanleene M, Saldanha Z, Cloyd KL, et al. Transplantation of human fetal blood stem cells in the osteogenesis imperfecta mouse leads to improvement in multiscale tissue properties. *Blood* 2011;117:1053-60.

32. Horwitz EM, Prockop DJ, Fitzpatrick LA, et al. Transplantability and therapeutic effects of bone marrow-derived mesenchymal cells in children with osteogenesis imperfecta. *Nat Med* 1999;5:309-13.

33. Horwitz EM, Prockop DJ, Gordon PL, et al. Clinical responses to bone marrow transplantation in children with severe osteogenesis imperfecta. *Blood* 2001;97:1227-31.

34. Horwitz EM, Gordon PL, Koo WK, et al. Isolated allogeneic bone marrow-derived mesenchymal cells engraft and stimulate growth in children with osteogenesis imperfecta: Implications for cell therapy of bone. *Proc Natl Acad Sci U S A* 2002;99:8932-7.

35. Infante A, Gener B, Vazquez M, et al. Reiterative infusions of MSCs improve pediatric osteogenesis imperfecta eliciting a pro-osteogenic paracrine response: TERCELOI clinical trial. *Clin Transl Med* 2021;11:e265.

36. Infante A, Cabodevilla L, Gener B, et al. Circulating TGF-beta Pathway in Osteogenesis Imperfecta Pediatric Patients Subjected to MSCs-Based Cell Therapy. *Front Cell Dev Biol* 2022;10:830928.

37. Le Blanc K, Gotherstrom C, Ringden O, et al. Fetal mesenchymal stem-cell engraftment in bone after in utero transplantation in a patient with severe osteogenesis imperfecta. *Transplantation* 2005;79:1607-14.

38. Götherström C, Westgren M, Shaw SW, et al. Pre- and postnatal transplantation of fetal mesenchymal stem cells in osteogenesis imperfecta: a two-center experience. *Stem Cells Transl Med* 2014;3:255-64.

39. Pollitt R, McMahon R, Nunn J, et al. Mutation analysis of COL1A1 and COL1A2 in patients diagnosed with osteogenesis imperfecta type I-IV. *Hum Mutat* 2006;27:716.

40. Kidszun A, Linebarger J, Walter JK, et al. What If the Prenatal Diagnosis of a Lethal Anomaly Turns Out to Be Wrong? *Pediatrics* 2016;137

41. Spencer RN, Hecher K, Norman G, et al. Development of standard definitions and grading for Maternal and Fetal Adverse Event Terminology. *Prenat Diagn* 2022;42:15-26.

42. Rapoport M, Bober MB, Raggio C, et al. The patient clinical journey and socioeconomic impact of osteogenesis imperfecta: a systematic scoping review. *Orphanet J Rare Dis* 2023;18:34.

43. Kolovos S, Javaid MK, Pinedo-Villanueva R. Hospital admissions of patients with osteogenesis imperfecta in the English NHS. *Osteoporos Int* 2021;32:1207-16.

44. Darba J, Marsa A. Hospital incidence, management and direct cost of osteogenesis imperfecta in Spain: a retrospective database analysis. *J Med Econ* 2020;23:1435-40.

45. Johansson M, Hermeren G, Sahlin NE. Paternal consent in prenatal research: ethical aspects. *Med Health Care Philos* 2020;23:325-31.

For peer review only - http://bmjopen.bmj.com/site/about/guidelines.xhtml

Table 1. Inclusion and Exclusion criteria for subjects in the BOOSTB4 trial.

For inclusion, all criteria should apply. For exclusion, one is enough to exclude.

Prenatal group: After birth and before the 2nd dose, subjects in the prenatal group will be assessed for the inclusion and exclusion criteria for the postnatal group.

**INCLUSION CRITERIA**		
**Postnatal group (n=15)**[1, 2]	**Prenatal group (n=3)**[1, 3]	**Control group (n=30–150 per subject)**[1]
1. Parent's/legal guardian's signed informed-consent form,<br>2. Clinical diagnosis of OI type 3 or severe 4 <u>AND</u><br>3. Molecular diagnosis of OI (Glycine substitution in the collagen triple-helix encoding region of either the *COL1A1* or *COL1A2* gene),<br>4. Age less than 18 months (calculated from gestational week 40+0, i.e. the corrected age),<br>5. Parent/legal guardian over 18 years of age.	1. Woman has signed the patient consent form,<br>2. Only women where termination of the pregnancy is no longer possible or where the women are committed to continue the pregnancy may be included in the trial,<br>3. Suspicion of OI type 3 or severe 4 in the fetus on ultrasound findings <u>AND</u><br>4. Molecular diagnosis of OI in the fetus (Glycine substitution in the collagen triple-helix encoding region of either the *COL1A1* or *COL1A2* gene),<br>5. Gestation age between 16+0 and 35+6 weeks+days,<br>6. Pregnant women over 18 years of age.	*Matched historical controls:*<br>1. Parent's/legal guardian's signed informed-consent form,<br>2. Clinical and molecular diagnosis of OI (Glycine substitution in the collagen triple-helix encoding region of either the *COL1A1* or *COL1A2* gene),<br>3. Data on fractures and growth is available,<br>4. Parent/legal guardian over 18 years of age.<br>*Prospective untreated controls:*<br>- Postnatal participation: The inclusion criteria for the postnatal group apply.<br>- Prenatal participation: The inclusion criteria for the prenatal group apply, except inclusion criteria 2.

[1]All participants (the postnatal, prenatal and control groups) will receive bisphosphonate treatment.

[2]At least one dose of bisphosphonate must be administered before the first dose of stem cells.

[3]The first dose of bisphosphonates will be administered after birth.

For peer review only

**EXCLUSION CRITERIA**		
**Postnatal group (n=15)**	**Prenatal group (n=3)**	**Control group (n=30–150)**
1. Existence of other known disorder that might interfere with the treatment, such as, but not limited to organ disfunction (for example liver or renal failure or bronchopulmonary dysplasia), congenital heart defect, hypoxic encephalopathy I-III, severe neurological problems, immune deficiencies, muscle diseases, severe malformations or syndromes diagnosed by clinical examination,<br>2. Any contraindication for invasive procedures such as a moderate/severe bleeding tendency,<br>3. Known risk factors for clotting, such as, but not limited to previous blood clot, family history of clots, clotting disorder (inherited or acquired), heart failure, inflammatory disorders (for example lupus, rheumatoid arthritis, inflammatory bowel disease),<br>4. Positive Donor Specific Antibody-test,<br>5. Known allergy/hypersensitivity to Fungizone and/or Gensumycin,<br>6. Abnormal karyotype or other confirmed genetic syndromes,<br>7. Oncologic disease (previous or current malignancy),<br>8. Inability to comply with the trial protocol and follow-up schedule,<br>9. Inability to understand the information and to provide informed consent.	1. Multiple pregnancy,<br>2. Co-existence of other disorder that might interfere with the treatment, as judged by the Investigator or the patient's obstetrician,<br>3. Abnormal fetal karyotype or other confirmed genetic syndrome,<br>4. Any contraindication for invasive procedures such as a bleeding tendency or contagious infections, such as, but not limited to HIV, Syphilis, Hepatitis B, Hepatitis C or other known infectious diseases that can harm the fetus,<br>5. Known risk factors for clotting, such as, but not limited to previous blood clot, family history of clots, clotting disorder (inherited or acquired), heart failure, inflammatory disorders (for example lupus, rheumatoid arthritis, inflammatory bowel disease),<br>6. Positive Donor Specific Antibody-test,<br>7. Known allergy/hypersensitivity to Fungizone and/or Gensumycin,<br>8. Oncologic disease in woman or fetus (previous or current malignancy),<br>9. Unwilling to or cannot undergo delivery by Caesarean section,<br>10. Inability to comply with the trial protocol and follow-up schedule,<br>11. Inability to understand the information and to provide informed consent.	*Matched historical controls (15/subject):*<br>1. Existence of other disorder that might interfere with the trial. No lung hypoplasia (type 2 OI),<br>2. Abnormal karyotype.<br>*Prospective untreated controls (n=up to 30):*<br>- Postnatal participation: The exclusion criteria, except exclusion criterium 2, 3, 4 and 5 (Contraindication for invasive procedure, Known risk factor for clotting, Positive Donor Specific Antibody-test and Known allergy/hypersensitivity to Fungizone and/or Gensumycin) for the postnatal group apply.<br>- Prenatal participation: The exclusion criteria, except exclusion criterium 1, 4, 5, 6 and 7 (Multiple pregnancy, Contraindication for invasive procedure, Known risk factor for clotting, Positive Donor Specific Antibody-test and Known allergy/hypersensitivity to Fungizone and/or Gensumycin) for the prenatal group apply.

For peer review only

For peer review only - http://bmjopen.bmj.com/site/about/guidelines.xhtml

Table 2. Matching of the controls.

One to five historical controls per subject and up to 30 prospective controls in total will be included and matched to each subject.

MATCHING OF THE CONTROLS	
The controls historical and untreated prospective) will be individually matched to each trial subject:	
1. Type of OI[1]:	- Type 3 with type 3.<br>- Severe type 4 with Severe type 4.
2. Age at onset of bisphosphonate therapy:	- Start of bisphosphonate treatment in the first 2 months of life.<br>- Start of bisphosphonate treatment from 2–12 months of life.
If possible (if more than 5 controls have been identified), matching will also be made on:	
3. Sex	
4. Identical OI pathogenic sequence variant	
5. Time for OI diagnosis	- A control matched with a subject in the postnatal group was diagnosed[2] postnatally.<br>- A control matched with a subject in the prenatal group was diagnosed[2] prenatally.

[1]No mixed OI types (for e.g. OI type 3/4, is allowed).

[2]Clinical diagnosis, molecular diagnosis not required at this time point.

For peer review only

For peer review only - http://bmjopen.bmj.com/site/about/guidelines.xhtml

FIGURE LEGENDS

Figure 1. Overview of the trial schedule.

The BOOSTB4 trial consists of 2 parts; Part 1 that includes information, consent, screening and inclusion. This is followed by 4 doses of BOOST cells at 4±1 month intervals with immediate in-patient follow-up over 48 hours after dose 1–2, and over 24 hours after dose 3–4 (48 hours for dose 1–3 in the prenatal group). Part 1 of the trial also includes the primary follow-up at 6 and 12 months after the 4th and last dose. Part 2 of the trial consists of yearly long-term follow-up over 8 years until 10 years after the first dose.

For peer review only

For peer review only - http://bmjopen.bmj.com/site/about/guidelines.xhtml

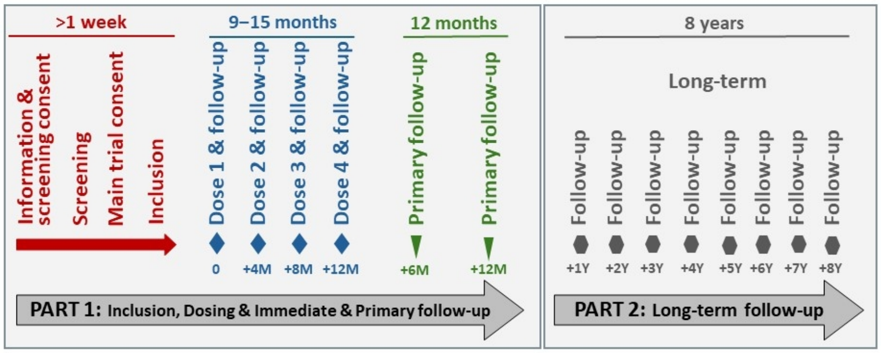

Figure 1. Overview of the trial schedule.

The BOOSTB4 trial consist of 2 parts; Part 1 that includes information, consent, screening and inclusion. This is followed by 4 doses of BOOST cells at 4±1 month intervals with immediate in-patient follow-up over 48 hours after dose 1–2, and over 24 hours after dose 3–4 (48 hours for dose 1–3 in the prenatal group). Part 1 of the trial also includes the primary follow-up at 6 and 12 months after the 4th and last dose. Part 2 of the trial consist of yearly long-term follow-up over 8 years until 10 years after the first dose.

Figure 1

275x190mm (96 x 96 DPI)

CLINICAL TRIAL SYNOPSIS

BOOSTB4

Boost Brittle Bones Before Birth

TRIAL REGISTRATION DATA SET	
**Data category**	**Information**
Registry trial identifying number	EudraCT no: 2015-003699-60, ClinicalTrials.gov ID: NCT03706482
Secondary identifying numbers	KIBB01
Sources of monetary support	Academic: European Union's Horizon 2020 research and innovation programme under grant agreement No 681045, the Swedish Research Council, Region Stockholm in Sweden
Sponsor	Karolinska Institutet
Contact for queries	boostb4@clintec.ki.se
Title	An exploratory, open label, multiple dose, multicentre phase I/II trial evaluating safety and efficacy of postnatal or prenatal and postnatal intravenous administration of allogeneic expanded fetal mesenchymal stem cells for the treatment of severe Osteogenesis Imperfecta compared with a combination of historical and untreated prospective controls.
Trial code	BOOSTB4
Countries of recruitment	Sweden, Netherlands, United Kingdom
Health condition studied	Osteogenesis Imperfecta type 3 and severe type 4
Intervention	Intravenous administration of BOOST cells
Investigational Medicinal Product	BOOST cells (expanded human 1st trimester fetal liver-derived mesenchymal stem cells)
Study type	Interventional
	Open label
	Primary purpose: Treatment of chronic condition
	Phase I/II
Trial groups	Postnatal group (4 postnatal doses), n=15
	Prenatal group (1 prenatal dose & 3 postnatal doses), n=3
	Prospective control group, n=up to 30
	Historical control group, n=1–5 per subject
Date of first enrolment	March 2020
Target sample size	Initially n=15 in the postnatal group and n=15 in the prenatal group, later amended to n=15 in the postnatal group and n=3 in the prenatal group
Recruitment status	Completed
Primary outcomes	Safety and tolerability
Key secondary outcomes	Efficacy: fracture incidence, growth, bone mineral density

For peer review only - http://bmjopen.bmj.com/site/about/guidelines.xhtml

Protocol Summary

PROTOCOL IDENTITY

Trial Title: Boost Brittle Bones Before Birth (BOOSTB4)

An exploratory, open label, multiple dose, multicentre phase I/II trial evaluating safety and efficacy of postnatal or prenatal and postnatal intravenous administration of allogeneic expanded fetal mesenchymal stem cells for the treatment of severe Osteogenesis Imperfecta compared with a combination of historical and untreated prospective controls.

EudraCT number	Protocol Number	Trial Code	Phase of Development
2015-003699-60	KIBB01	BOOSTB4	I/II

Sponsor: Karolinska Institutet, Stockholm, Sweden

Sponsor's representative: Associate Professor Cecilia Götherström, Karolinska Institutet, Department of Clinical Science, Technology and Intervention, Division of Obstetrics and Gynaecology, Stockholm, Sweden

Principal Coordinating Investigator: Dr Eva Åström, Pediatric Neurology and Musculoskeletal disorders and Home care, Astrid Lindgren Children's Hospital at Karolinska University Hospital, Stockholm, Sweden

Principal Investigator: Dr Eva Åström, Pediatric Neurology and Musculoskeletal disorders and Home care, Astrid Lindgren Children's Hospital at Karolinska University Hospital, Stockholm, Sweden

Trial centres: Karolinska University Hospital, Sweden[1]; University College London Hospitals NHS Foundation Trust[2] and Great Ormond Street Hospital for Children NHS Foundation Trust[2], the United Kingdom; University Hospital Cologne[2], Germany and Leiden University Medical Centre[3] and University Medical Centre Utrecht[3], the Netherlands.

[1]Performing the active part of the trial, i.e. dose administration and immediate, primary and long-time follow-up, and inclusion of historical and prospective controls.

[2]Performing inclusion of historical controls.

[3]Performing inclusion of historical and prospective controls and primary and long-time follow-up of two Dutch subjects.

INVESTIGATIONAL MEDICINAL PRODUCT (IMP)

Test Product: BOOST cells (allogeneic expanded human 1st trimester liver-derived mesenchymal stem cells [MSC])

Pharmaceutical Form: Cell suspension

Dose: $3x10^6$ viable BOOST cells/kg body weight

Route of Administration: Intravenous administration postnatally or prenatally

Manufacturer: Karolinska Cell Therapy Centre, Vecura at Karolinska University Hospital in Sweden

For peer review only - http://bmjopen.bmj.com/site/about/guidelines.xhtml

TRIAL DESIGN

Hypothesis: Early administration of MSC will ameliorate severe types of OI.

Trial design:
This will be a multi-centre, open label, multiple-dose, phase I/II trial of allogeneic expanded human first trimester fetal MSC administration for the treatment of Osteogenesis Imperfecta (OI) type 3 or severe type 4 compared with historical and untreated prospective controls.

The trial is divided into 2 parts where Part 1 includes administration of 4 postnatal doses per subject and Part 2 includes administration of one prenatal and 3 postnatal doses per subject:

Part 1: Postnatal administration (n=15 in total)

- Administration of four postnatal doses of MSC to 15 subjects
- A safety assessment is performed by the Data Safety Monitoring Board (DSMB) of the first MSC administration to the first 5 subjects[1] in any participating country treated postnatally.

[1]The 5 subjects included and administered with 1 MSC dose postnatally in Part 1 of the trial will receive the additional 3 MSC doses as planned, i.e. at +4, +8 and +12 months after the first MSC dose. Available data from such administration will be included in the data set sent to the DSMB for the safety assessment. There will be no planned pause in the postnatal administration during the safety assessment by the DSMB for opening of Part 2 of the trial. The Stopping rules on subject level apply to all steps (see Section 10.9).

Part 2: Prenatal administration (n=3 in total)

- One prenatal MSC dose to 3 subjects[2].

[2]The 3 subjects included and administered with 1 MSC dose prenatally in Part 2 of the trial will receive the additional 3 postnatal MSC doses as planned, i.e. at +4, +8 and +12 months after the prenatal dose. The Stopping rules on subject level apply to all steps (see Section 10.9).

Duration:
The trial is divided into two periods where Period 1 includes the administration of MSC and immediate and primary follow-up, and Period 2 that includes the long-time follow-up (see the Trial schedule below):

Period 1: Four doses of MSC every 4 months with immediate follow-up after each MSC dose, and primary follow-up at 6 and 12 months after the fourth and last MSC dose

Period 2: Yearly long-time follow-up until 10 years after the first MSC dose

Subjects in the postnatal group will be included, receive the first dose and evaluated as soon as possible after birth up to 18 months of corrected age. They will receive 3 additional doses at +4, +8 and +12 months (±1 month) after the 1st dose.

Subjects in the prenatal group will be included in the trial and receive the 1st dose prenatally (between 16+0 – 35+6 weeks+days of gestation) and receive 3 additional postnatal doses at +4 (or as soon as possible after birth), +8 and +12 months (±1 month) after the 1st dose.

Subjects can be enrolled during the initial 3 years of the trial. The expected duration of treatment is 12 months, the minimum treatment time is 9 months and the maximum treatment time is 15 months.

For peer review only

For peer review only - http://bmjopen.bmj.com/site/about/guidelines.xhtml

Trial Schedules in Period 1 (GW=gestation weeks in weeks+days. All postnatal time points are ± 1 month)**:**

Postnatal group

Diagnosis	Dose 1 & immediate follow-up	Dose 2 & immediate follow-up	Dose 3 & immediate follow-up	Dose 4 & immediate follow-up	Primary follow-up	Primary follow-up
Clinical & molecular diagnosis of type 3 / severe type 4 OI	As soon as possible after birth up to 18 months of age	+4 months after dose 1	+4 months after dose 2	+4 months after dose 3	+6 months after dose 4	+12 month after dose 4

Prenatal group

Diagnosis	Dose 1 & immediate follow-up	Post prenatal dose follow-up	Birth	Dose 2 & immediate follow-up	Dose 3 & immediate follow-up	Dose 4 & immediate follow-up	Primary follow-up	Primary follow-up
Clinical & molecular diagnosis of type 3 / severe type 4 OI	Gestation weeks 16+0 – 35+6	Every 2 weeks up to birth		+4 months after dose 1	+4 months after dose 2	+4 months after dose 3	+6 months after dose 4	+12 months after dose 4

TRIAL OBJECTIVES

Primary objectives:

To assess safety and tolerability in the child, fetus and woman after postnatal or prenatal and postnatal intravenous administration of 4 doses of BOOST cells every 4 months in subjects with OI type 3 or severe type 4.

Secondary objectives:

To assess the effect of intravenous administration of 4 doses of BOOST cells every 4 months in subjects with OI type 3 or severe type 4 on:

1. Number of fractures from baseline to primary and long-time follow-up
2. Time (days) to first fracture after last dose
3. Number of fractures at birth (prenatal treatment group only, and postnatal treatment group when available)
4. Bone mineral density (BMD)
5. Growth
6. Clinical status of OI
7. Biochemical bone turnover

Exploratory objectives

1. To study the impact of 4 doses of fetal MSC on Quality of Life (QoL)
2. To study the extent of donor cell engraftment in tissue samples
3. To study paracrine effects of 4 doses of fetal MSC
4. To study the effect of 4 doses of fetal MSC on endogenous immune cells
5. To study non-invasive prenatal diagnosis of OI (will not be used in the diagnostic procedure in the trial)

TRIAL ENDPOINTS

Primary Endpoints:

The primary endpoint is safety and tolerability measured as seriousness, severity and frequency of MSC administration related adverse events (AEs), with specific focus on the following:

For peer review only

For peer review only - http://bmjopen.bmj.com/site/about/guidelines.xhtml

1. Vital signs in conjunction with the MSC administration
2. Transfusion reactions (administration toxicity, allergy, embolism)
3. Immune reaction with or without symptoms of inflammation, potentially resulting in rejection of the cells or development of donor-specific antibodies:
   - Allergy or Hypersensitivity responses to antibiotics or antimycotics
   - Development of Fetal Bovine Serum-specific antibodies
   - Hypersensitivity responses to Human Serum Albumin
   - Hypersensitivity to impurities in the IMP
4. Prenatal complications (miscarriage/intrauterine fetal death, premature birth, infection *in utero* or persistent [>1 min] fetal bradycardia) in the prenatal group
5. Adverse effects of feto-maternal transmission of donor cells in the prenatal group
6. Tumourigenicity
7. Mortality/morbidity

Key Secondary Endpoint:

1. Number of fractures from baseline to primary and long-time follow-up

Secondary Endpoints:

1. Time (days) to first fracture after last dose
2. Number of fractures at birth (prenatal group, and postnatal group when available)
3. Change in BMD (g/cm^2)
4. Growth (cm and kg)
5. Change in clinical status of OI based on parameters defined under efficacy assessments described below
6. Change in biochemical bone turnover

Exploratory endpoints:

1. The QoL will be assessed through the Infant Toddler Quality of Life Questionnaire™ (ITQOL)
2. Tissue samples (bone, bone marrow, muscle and skin, placenta, amniotic fluid, umbilical cord, umbilical cord blood, peripheral blood) will be collected in conjunction with clinically indicated surgery and at birth (prenatal group only) and analysed for donor cell engraftment
3. Paracrine effects will be analysed from plasma isolated from peripheral blood
4. Effect on immune cells will be analysed from peripheral blood
5. Non-invasive prenatal diagnosis will be studied during the trial

POPULATION OF TRIAL SUBJECTS

Description of Trial Subjects:

1. Infants diagnosed with OI type 3 or severe type 4 on clinical grounds with a confirmatory sequence variant (mutation) in one or other of the type I collagen genes (*COL1A1* and *COL1A2*) (n=15)
2. Pregnant women whose fetus has been diagnosed with OI type 3 or severe type 4 prenatally on ultrasound parameters with a confirmatory sequence variant (mutation) in one or other of the type I collagen genes (*COL1A1* and *COL1A2*) (n=3)
3. Controls diagnosed with OI type 3 or severe type 4 on clinical grounds with a confirmatory sequence variant (mutation) in one or other of the type I collagen genes (*COL1A1* and *COL1A2*):
   a) Matched historical controls (n=30-150) will be identified in national registries
   b) Prospective untreated controls (n=up to 30). Subjects eligible for the trial but not willing/able to participate.

For peer review only

For peer review only - http://bmjopen.bmj.com/site/about/guidelines.xhtml

Only patients diagnosed with qualitative (Glycine) substitution in the collagen triple-helix encoding region of either the *COL1A1* or *COL1A2* gene will be included in the trial. The diagnosis will be made both clinically and with molecular analysis by a multidisciplinary team.

No formal power calculations have been performed. OI is a rare disease and a limited number of subjects can be included each year. Based on the incidence, it is reasonable to include 30 patients in this first trial on the grounds of feasibility, suitable time period and for the application of descriptive statistical analyses.

INCLUSION CRITERIA

For inclusion, all criteria should apply.

Postnatal group

1. Parent's/legal guardian's signed informed-consent form
2. Clinical diagnosis of OI type 3 or 4 AND
3. Molecular diagnosis of OI (Glycine substitution in the collagen triple-helix encoding region of either the *COL1A1* or *COL1A2* gene)
4. Age less than 18 months (calculated from gestational week 40+0, i.e. the corrected age)
5. Parent/legal guardian over 18 years of age

Prenatal group:

1. Woman has signed the patient consent form
2. Only women where termination of the pregnancy is no longer possible or where the women are committed to continue the pregnancy may be included in the trial
3. Suspicion of OI type 3 or 4 in the fetus on ultrasound findings AND
4. Molecular diagnosis of OI in the fetus (Glycine substitution in the collagen triple-helix encoding region of either the *COL1A1* or *COL1A2* gene)
5. Gestation age between 16+0 and 35+6 weeks+days
6. Pregnant women over 18 years of age

After birth and before the 2nd dose, subjects in the prenatal group will be assessed for the inclusion criteria for the postnatal group.

Control group:

a) Matched historical controls

1. Parent's/legal guardian's signed informed-consent form
2. Clinical and molecular diagnosis of OI (Glycine substitution in the collagen triple-helix encoding region of either the *COL1A1* or *COL1A2* gene)
3. Data on fractures and growth is available
4. Parent/legal guardian over 18 years of age

b) Prospective untreated controls

Postnatal participation: The inclusion criteria for the postnatal group apply

Prenatal participation: The inclusion criteria for the prenatal group apply, except inclusion criteria 2.

For peer review only - http://bmjopen.bmj.com/site/about/guidelines.xhtml

EXCLUSION CRITERIA

For exclusion, one is enough to exclude.

Postnatal group:

1. Existence of other known disorder that might interfere with the treatment, such as, but not limited to organ disfunction (for example liver or renal failure or bronchopulmonary dysplasia), congenital heart defect, hypoxic encephalopathy I-III, severe neurological problems, immune deficiencies, muscle diseases, severe malformations or syndromes diagnosed by clinical examination
2. Any contraindication for invasive procedures such as a moderate/severe bleeding tendency
3. Known risk factors for clotting, such as, but not limited to previous blood clot, family history of clots, clotting disorder (inherited or acquired), heart failure, inflammatory disorders (for example lupus, rheumatoid arthritis, inflammatory bowel disease)
4. Positive Donor Specific Antibody-test
5. Known allergy/hypersensitivity to Fungizone and/or Gensumycin
6. Abnormal karyotype or other confirmed genetic syndromes
7. Oncologic disease (previous or current malignancy)
8. Inability to comply with the trial protocol and follow-up schedule
9. Inability to understand the information and to provide informed consent

Prenatal group:

1. Multiple pregnancy
2. Co-existence of other disorder that might interfere with the treatment, as judged by the Investigator or the patient's obstetrician
3. Abnormal fetal karyotype or other confirmed genetic syndrome
4. Any contraindication for invasive procedures such as a bleeding tendency or contagious infections, such as, but not limited to HIV, Syphilis, Hepatitis B, Hepatitis C or other known infectious diseases that can harm the fetus
5. Known risk factors for clotting, such as, but not limited to previous blood clot, family history of clots, clotting disorder (inherited or acquired), heart failure, inflammatory disorders (for example lupus, rheumatoid arthritis, inflammatory bowel disease)
6. Positive Donor Specific Antibody-test
7. Known allergy/hypersensitivity to Fungizone and/or Gensumycin
8. Oncologic disease in woman or fetus (previous or current malignancy)
9. Unwilling to or cannot undergo delivery by Caesarean section
10. Inability to comply with the trial protocol and follow-up schedule
11. Inability to understand the information and to provide informed consent

After birth and before the 2nd dose, subjects in the prenatal group will be assessed for the exclusion criteria for the postnatal group.

Control group:

a) Matched historical controls

1. Existence of other disorder that might interfere with the trial. No lung hypoplasia (type 2 OI).
2. Abnormal karyotype

b) Prospective untreated controls

Postnatal participation: The exclusion criteria, except exclusion criterium 2, 3, 4, and 5 (Contraindication for invasive procedure, Known risk factor for clotting, Positive Donor Specific

For peer review only

For peer review only - http://bmjopen.bmj.com/site/about/guidelines.xhtml

Antibody-test and Known allergy/hypersensitivity to Fungizone and/or Gensumycin), for the postnatal group apply.

Prenatal participation: The exclusion criteria, except exclusion criterium 1, 4, 5, 6 and 7 (Multiple pregnancy, Contraindication for invasive procedure, Known risk factor for clotting, Positive Donor Specific Antibody-test and Known allergy/hypersensitivity to Fungizone and/or Gensumycin), for the prenatal group apply.

MATCHING OF THE CONTROLS

Parents/authorized representatives of children diagnosed with OI type 3 or severe OI type 4 will be informed about the trial and asked to give consent to the child's participation in the trial as a matched historical control. Subjects eligible for the trial but not willing/able to participate in the trial will be asked for consent to be included as prospective controls.

The controls (historical and untreated prospective) will be individually matched to each trial subject:

1. Type of OI*:
   Type 3 with Type 3
   Severe Type 4 with Severe Type 4
   *No mixed OI types (for e.g. OI type 3/4, is allowed)
2. Age at onset of BP therapy:
   Start of BP treatment in the first 2 months of life.
   Start of BP treatment from 2–12 months of life.

If possible (if more than 5 controls have been identified), matching will also be made on:

3. Sex
4. Identical OI mutation
5. Time for diagnosis:
   A control matched with a subject in the postnatal group was diagnosed** postnatally
   A control matched with a subject in the prenatal group was diagnosed** prenatally
   **Clinical diagnosis, molecular diagnosis not required at this time point.

For peer review only

For peer review only - http://bmjopen.bmj.com/site/about/guidelines.xhtml

BOOSTB4: A clinical research trial of a new intervention for brittle bone disease

SCREENING CONSENT FORM FOR THE POSTNATAL TRIAL GROUP

TO BE COMPLETED BEFORE SCREENING INVESTIGATIONS TAKE PLACE

Consent part 1

I confirm I have read and understood the Participant Information for the above trial. I confirm that I have received sufficient explanation of the BOOSTB4 Clinical Trial and have had the opportunity to consider the information and ask questions which have been answered satisfactorily. I have been given the Participant Information to keep.

I understand that my child's participation is voluntary and that I am free to withdraw them at any time without giving any reason, without their medical care or legal rights being affected.

I give consent for members of the BOOSTB4 Clinical Trial Team to review my child's medical records and use the data contained within, including access to all investigation results relevant for the purposes of this trial.

I give consent for members of the BOOSTB4 Clinical Trial Team to perform a screening investigation and a screening examination of my child as detailed in the Participant Information.

I agree that biological samples collected from my child are a gift, and can be used in the research described in the Participant Information.

I agree that all samples can be stored coded in the Stockholm Medical Biobank (reg. no. 914).

I agree that all coded samples can be transferred to other hospitals or laboratories in other countries for specialist tests.

For peer review only - http://bmjopen.bmj.com/site/about/guidelines.xhtml

Consent part 1

Patient Trial Identification Number: ___________________________ (filled in by the trial)

Date of Birth (dd/mm/yyyy) / social security no (personnummer): ____________________

Name of parent	Date (dd/mm/yyyy)	Signature
Name of parent (optional)	Date (dd/mm/yyyy)	Signature
Name of person taking consent	Date (dd/mm/yyyy)	Signature
Name of interpreter (if applicable)	Date (dd/mm/yyyy)	Signature

For peer review only

2 copies required: Original for Investigator Site File and copy for participant.

For peer review only - http://bmjopen.bmj.com/site/about/guidelines.xhtml

Karolinska Institutet

BOOSTB4
Boost Brittle Bones Before Birth

KAROLINSKA
UNIVERSITETSSJUKHUSET

Consent part 2

Voluntary, mark the Yes/No boxes according to your consent.

	YES	NO
I agree that all biological samples can be stored coded at the Stockholm Medical Biobank (reg. no. 914) for future ethically approved research.	☐	☐

Name of parent	Date (dd/mm/yyyy)	Signature
Name of parent (optional)	Date (dd/mm/yyyy)	Signature
Name of person taking consent	Date (dd/mm/yyyy)	Signature
Name of interpreter (if applicable)	Date (dd/mm/yyyy)	Signature

For peer review only - http://bmjopen.bmj.com/site/about/guidelines.xhtml

Karolinska Institutet
BOOSTB4
Boost Brittle Bones Before Birth
KAROLINSKA UNIVERSITETSSJUKHUSET

BOOSTB4: A clinical research trial of a new intervention for brittle bone disease

MAIN TRIAL CONSENT FORM FOR THE POSTNATAL TRIAL GROUP

TO BE COMPLETED AFTER SCREENING INVESTIGATIONS HAVE BEEN PERFORMED AND ELIGIBILITY CONFIRMED

Consent part 1

I confirm I have read and understood the Participant Information for the above trial. I confirm that I have received sufficient explanation of the BOOSTB4 Clinical Trial and have had the opportunity to consider the information and ask questions which have been answered satisfactorily. I have been given the Participant Information to keep.

I understand that my child's participation is voluntary and that I am free to withdraw them at any time without giving any reason, without their medical care or legal rights being affected.

I understand that relevant sections of my child's medical notes and data collected during the trial may be looked at by responsible individuals from national and international competent authorities, the BOOSTB4 team medical doctors and clinical trial monitors that inspect trial safety, where it is relevant to my child's taking part in this research. I give permission for these individuals to have access to my child's records. This includes medical notes and data collected at the clinical research site, medical notes and data collected within the health care (i.e. the general/family doctor and other community services). I consent to that data from my child are treated as described in the Participant Information.

I agree to my child's doctor and local hospital being informed of my child's participation in the trial. I agree that they can be contacted for medical information or obtaining up-to-date contact details.

I agree to my child taking part in the BOOSTB4 Clinical Trial.

I agree that my child can not participate in another clinical trial before the 12-month follow-up after the last stem cell dose, and that any participation in other trials must be discussed with the trial doctor.

I agree that biological samples collected from my child are a gift as described in the Participant Information.

I agree that all samples can be stored coded in the Stockholm Medical Biobank (reg. no. 914).

For peer review only - http://bmjopen.bmj.com/site/about/guidelines.xhtml

I agree that all coded samples can be transferred to other hospitals or laboratories in other countries for specialist tests.

I understand that this study using the samples may include genetic research aimed at understanding the genes related to OI and how the environment affects them (epigenetics) but that the results of these investigations are unlikely to have any implications for my child personally.

I agree that all discoveries (intellectual property) are a gift to the researchers and understand that my child will not benefit financially if the research leads to a new treatment or medical test.

I agree that I can be contacted by telephone to collect follow-up information about my child.

Consent part 1

Patient Trial Identification Number: ____________________________ (filled in by the trial)

Date of Birth (dd/mm/yyyy) / social security no: ____________________________________

Name of parent	Date (dd/mm/yyyy)	Signature
Name of parent (optional)	Date (dd/mm/yyyy)	Signature
Name of person taking consent	Date (dd/mm/yyyy)	Signature
Name of interpreter (if applicable)	Date (dd/mm/yyyy)	Signature

For peer review only - http://bmjopen.bmj.com/site/about/guidelines.xhtml

For peer review only

Consent part 2

Voluntary, mark the Yes/No boxes according to your consent.

	YES	NO
I agree that all biological samples can be stored coded in the Stockholm Medical Biobank (reg. no. 914) for future ethically approved research.	☐	☐
I agree to give consent for photographs and video footage to be taken of trial procedures; the anonymised images may be used for educational purposes or be published.	☐	☐
I agree to give contact details for a relative / friend that the BOOSTB4 trial team can contact if they are unable to get in touch with me in the future.	☐	☐
I agree to the BOOSTB4 trial team contacting me in the future about trial related activities and events.	☐	☐
I would like to receive a summary of the trial results (it will take up to five years).	☐	☐

Name of parent	Date (dd/mm/yyyy)	Signature
Name of parent (optional)	Date (dd/mm/yyyy)	Signature
Name of person taking consent	Date (dd/mm/yyyy)	Signature
Name of interpreter (if applicable)	Date (dd/mm/yyyy)	Signature

2 copies required: Original for Investigator Site File and copy for participant.

For peer review only

For peer review only - http://bmjopen.bmj.com/site/about/guidelines.xhtml

BOOSTB4: A clinical research trial of a new intervention for brittle bone disease

SCREENING CONSENT FORM FOR THE PRENATAL TRIAL GROUP

TO BE COMPLETED BEFORE SCREENING INVESTIGATIONS TAKE PLACE

Consent part 1

I confirm I have read and understood the Participant Information for the above trial. I confirm that I have received sufficient explanation of the BOOSTB4 Clinical Trial and have had the opportunity to consider the information and ask questions which have been answered satisfactorily. I have been given the Participant Information to keep.

I understand that my participation is voluntary and that I am free to withdraw my consent at any time without giving any reason, without my medical care or legal rights being affected.

I give consent for members of the BOOSTB4 Clinical Trial Team to review my medical records and use the data contained within, including access to all prenatal diagnostic tests. I consent to that information about me is processed as described in the participant information.

I give consent for members of the BOOSTB4 Clinical Trial Team to perform a pre investigation (screening) for the above mentioned trial, including investigations such as ultrasound, as it is described in the participant information.

I agree that biological samples collected from me and my child are a gift, and can be used in the research on stem cell infusion and OI as described in the Participant Information.

I agree that all samples can be stored coded in the Stockholm Medical Biobank (reg. no. 914).

I agree that all coded samples can be transferred to other hospitals or laboratories in other countries for specialist tests.

For peer review only - http://bmjopen.bmj.com/site/about/guidelines.xhtml

Consent part 1

Patient Trial Identification Number: ____________________________ (filled in by the trial)

Date of Birth (dd/mm/yyyy) / social security number pregnant woman:

____

Name of patient (pregnant woman)	Date (dd/mm/yyyy)	Signature
Name of parent (optional)	Date (dd/mm/yyyy)	Signature
Name of person taking consent	Date (dd/mm/yyyy)	Signature
Name of interpreter (if applicable)	Date (dd/mm/yyyy)	Signature

For peer review only
For peer review only - http://bmjopen.bmj.com/site/about/guidelines.xhtml

Karolinska Institutet | BOOSTB4 Boost Brittle Bones Before Birth | KAROLINSKA UNIVERSITETSSJUKHUSET

Consent part 2

Voluntary, mark the Yes/No boxes according to your consent.	YES	NO
I agree that the following samples are used for the development of a non-invasive prenatal test of OI:<br>- A blood sample<br>- Leftover amniotic fluid / placenta / DNA (no new samples are taken)<br>I also agree that the biological samples can be stored coded in Stockholm's medical biobank (reg. No. 914) to be used for research related to the development of a non-invasive prenatal test, as described in the participant information, and that coded samples can sent to hospitals or laboratories in other countries for specific tests.	☐	☐
I agree that all biological samples can be stored coded at the Stockholm Medical Biobank (reg. no. 914) for future ethically approved research.	☐	☐

Name of patient (pregnant woman)	Date (dd/mm/yyyy)	Signature
Name of parent (optional)	Date (dd/mm/yyyy)	Signature
Name of person taking consent	Date (dd/mm/yyyy)	Signature
Name of interpreter (if applicable)	Date (dd/mm/yyyy)	Signature

2 copies required: Original for Investigator Site File and copy for participant.

For peer review only

For peer review only - http://bmjopen.bmj.com/site/about/guidelines.xhtml

Karolinska Institutet
BOOSTB4 Boost Brittle Bones Before Birth
KAROLINSKA UNIVERSITETSSJUKHUSET

BOOSTB4: A clinical research trial of a new intervention for brittle bone disease

<u>SCREENING</u> CONSENT FORM FOR THE PRENATAL TRIAL GROUP (FATHER)

TO BE COMPLETED BEFORE SCREENING INVESTIGATIONS TAKE PLACE

Consent part 1

I confirm I have read and understood the Participant Information for the above trial. I confirm that I have received sufficient explanation of the BOOSTB4 Clinical Trial and have had the opportunity to consider the information and ask questions which have been answered satisfactorily. I have been given the Participant Information to keep.

I understand that my participation is voluntary and that I am free to withdraw my consent at any time without giving any reason, without my medical care or legal rights being affected.

I give consent for members of the BOOSTB4 Clinical Trial Team to review my medical records and use the data contained within. I consent to that information about me is processed as described in the participant information.

I give consent to donate the following for the development of a non-invasive prenatal test: A blood sample.

I give consent to that the blood sample collected from me may be used for the described research related to the development of a non-invasive prenatal test, as described in the participant information.

I agree that all samples can be stored coded in the Stockholm Medical Biobank (reg. no. 914).

I agree that all coded samples can be transferred to other hospitals or laboratories in other countries for specialist tests.

For peer review only - http://bmjopen.bmj.com/site/about/guidelines.xhtml

Karolinska Institutet

BOOSTB4 Boost Brittle Bones Before Birth

KAROLINSKA UNIVERSITETSSJUKHUSET

Consent part 1

Patient Trial Identification Number: ____________________________ (filled in by the trial)

Date of Birth (dd/mm/yyyy) / social security number: ______________________________

Name of patient (father)	Date (dd/mm/yyyy)	Signature
Name of person taking consent	Date (dd/mm/yyyy)	Signature
Name of interpreter (if applicable)	Date (dd/mm/yyyy)	Signature

Consent part 2

Voluntary, mark the Yes/No boxes according to your consent.	YES	NO
I agree that all biological samples can be stored coded at the Stockholm Medical Biobank (reg. no. 914) for future ethically approved research.	☐	☐

Name of patient (father)	Date (dd/mm/yyyy)	Signature
Name of person taking consent	Date (dd/mm/yyyy)	Signature
Name of interpreter (if applicable)	Date (dd/mm/yyyy)	Signature

2 copies required: Original for Investigator Site File and copy for participant.

For peer review only - http://bmjopen.bmj.com/site/about/guidelines.xhtml

For peer review only

Karolinska Institutet

BOOSTB4 Boost Brittle Bones Before Birth

KAROLINSKA UNIVERSITETSSJUKHUSET

BOOSTB4: A clinical research trial of a new intervention for brittle bone disease

MAIN TRIAL CONSENT FORM FOR THE PRENATAL TRIAL GROUP

TO BE COMPLETED AFTER SCREENING INVESTIGATIONS HAVE BEEN PERFORMED AND ELIGIBILITY CONFIRMED

Consent part 1

I confirm I have read and understood the Participant Information for the above trial. I confirm that I have received sufficient explanation of the BOOSTB4 clinical trial and have had the opportunity to consider the information and ask questions which have been answered satisfactorily. I have been given the Participant Information to keep.

I understand that mine and my child's participation is voluntary and that I am free to withdraw them at any time without giving any reason, without our medical care or legal rights being affected.

I understand that relevant sections of mine and my child's medical notes and data collected during the trial may be looked at by responsible individuals from national and international competent authorities, the BOOSTB4 team medical doctors and clinical trial monitors that inspect trial safety, where it is relevant to mine and my child's participation in this research. I give permission for these individuals to have access to mine and my child's records. This includes medical notes and data collected at the clinical research site, medical notes and data collected within the health care (i.e. the general/family doctor and other community services). I consent to that data from me and my child are treated as described in the Participant Information.

I agree to mine and my child's doctor and local hospital being informed of our participation in the trial. I agree that they can be contacted for medical information or obtaining up-to-date contact details.

I agree to me and my child taking part in the BOOSTB4 clinical trial including the follow-up.

I agree that my child maybe can not participate in another clinical trial before the 12-month follow-up after the last stem cell dose, and that any participation in other trials must be discussed with the trial doctor.

I agree that biological samples collected from me and my child, including the placenta, amniotic fluid and umbilical cord, can be used in the described research related to stem cell infusion or OI, as described in the Participant Information.

For peer review only - http://bmjopen.bmj.com/site/about/guidelines.xhtml

I agree that all samples can be stored coded in the Stockholm Medical Biobank (reg. no. 914).

I agree that all coded samples can be transferred to other hospitals or laboratories in other countries for specialist tests.

I understand that this study using the samples may include genetic research aimed at understanding the genes related to OI and how the environment affects them (epigenetics) but that the results of these investigations are unlikely to have any implications for me or my child personally.

I agree that all discoveries (intellectual property) are a gift to the researchers and understand that I and my child will not benefit financially if the research leads to a new treatment or medical test.

I agree that I can be contacted by telephone to collect follow-up information about myself and my child.

Consent part 1

Patient Trial Identification Number: ________________________ (filled in by the trial)

Date of Birth (dd/mm/yyyy) / social security number: ___________________________

Name of patient (pregnant woman)	Date (dd/mm/yyyy)	Signature
Name of parent (optional)	Date (dd/mm/yyyy)	Signature
Name of person taking consent	Date (dd/mm/yyyy)	Signature
Name of interpreter (if applicable)	Date (dd/mm/yyyy)	Signature

2 copies required: Original for Investigator Site File and copy for participant.

For peer review only - http://bmjopen.bmj.com/site/about/guidelines.xhtml

Karolinska Institutet · BOOSTB4 Boost Brittle Bones Before Birth · KAROLINSKA UNIVERSITETSSJUKHUSET

Consent part 2

Voluntary, mark the Yes/No boxes according to your consent.	YES	NO
I agree that all biological samples can be stored coded in the Stockholm Medical Biobank (reg. no. 914) for future ethically approved research.	☐	☐
I agree to give consent for photographs and video footage to be taken of trial procedures; the anonymised images may be used for educational purposes or be published.	☐	☐
I agree to give contact details for a relative / friend that the BOOSTB4 trial team can contact if they are unable to get in touch with me in the future.	☐	☐
I agree to the BOOSTB4 trial team contacting me in the future about trial related activities and events.	☐	☐
I would like to receive a summary of the trial results (it will take up to five years).	☐	☐

Name of patient (pregnant woman)	Date (dd/mm/yyyy)	Signature
Name of parent (optional)	Date (dd/mm/yyyy)	Signature
Name of person taking consent	Date (dd/mm/yyyy)	Signature
Name of interpreter (if applicable)	Date (dd/mm/yyyy)	Signature

2 copies required: Original for Investigator Site File and copy for participant.

#4 BOOSTB4 Consent Form Prenatal Trial Group [29-10-2020] v1.1 SE to the
#2 BOOSTB4 Participant Information Prenatal Trial Group [29-10-2020] v1.1 SE

For peer review only
For peer review only - http://bmjopen.bmj.com/site/about/guidelines.xhtml

BOOSTB4: A clinical research trial of a new intervention for brittle bone disease

POST BIRTH CONSENT CONFIRMATION FOR THE PRENATAL TRIAL GROUP

TO BE COMPLETED AFTER A BABY WHICH RECEIVED BOOSTB4 PRENATALLY HAS BEEN BORN AND ELIGIBILITY RE-CONFIRMED

Consent part 1

I confirm I have read and understood the Participant Information for the above trial. I confirm that I have received sufficient explanation of the BOOSTB4 clinical trial and have had the opportunity to consider the information and ask questions which have been answered satisfactorily. I have been given the Participant Information to keep.

I understand that my child's participation is voluntary and that I am free to withdraw them at any time without giving any reason, without their medical care or legal rights being affected.

I understand that relevant sections of my child's medical notes and data collected during the trial may be looked at by responsible individuals from national and international competent authorities, the BOOSTB4 team medical doctors and clinical trial monitors that inspect trial safety, where it is relevant to my child's taking part in this research. I give permission for these individuals to have access to my child's records. This includes medical notes and data collected at the clinical research site, medical notes and data collected within the health care (i.e. the general/family doctor and other community services). I consent to that data from my child are treated as described in the Participant Information.

I agree to my child's doctor and local hospital being informed of my child's participation in the trial. I agree that they can be contacted for medical information or obtaining up-to-date contact details.

I agree to my child taking part in the BOOSTB4 clinical trial.

I agree that my child can not participate in another clinical trial before the 12-month follow-up after the last stem cell dose, and that any participation in other trials must be discussed with the trial doctor.

I agree that biological samples collected from my child are a gift as described in the Participant Information.

I agree that all samples can be stored coded in the Stockholm Medical Biobank (reg. no. 914).

For peer review only - http://bmjopen.bmj.com/site/about/guidelines.xhtml

I agree that all coded samples can be transferred to other hospitals or laboratories in other countries for specialist tests.

I understand that this study using the samples may include genetic research aimed at understanding the genes related to OI and how the environment affects them (epigenetics) but that the results of these investigations are unlikely to have any implications for my child personally.

I agree that all discoveries (intellectual property) are a gift to the researchers and understand that my child will not benefit financially if the research leads to a new treatment or medical test.

I agree that I can be contacted by telephone to collect follow-up information about my child.

Consent part 1

Patient Trial Identification Number: ____________________________ (filled in by the trial)

Date of Birth (dd/mm/yyyy) / social security number: ______________________________

Name of parent	Date (dd/mm/yyyy)	Signature
Name of parent	Date (dd/mm/yyyy)	Signature
Name of person taking consent	Date (dd/mm/yyyy)	Signature
Name of interpreter (if applicable)	Date (dd/mm/yyyy)	Signature

For peer review only - http://bmjopen.bmj.com/site/about/guidelines.xhtml

Consent part 2

Voluntary, mark the Yes/No boxes according to your consent.

	YES	NO
I agree that all biological samples can be stored coded in the Stockholm Medical Biobank (reg. no. 914) for future ethically approved research.	☐	☐
I agree to give consent for photographs and video footage to be taken of trial procedures; the anonymised images may be used for educational purposes or be published.	☐	☐
I agree to give contact details for a relative / friend that the BOOSTB4 trial team can contact if they are unable to get in touch with me in the future.	☐	☐
I agree to the BOOSTB4 trial team contacting me in the future about trial related activities and events.	☐	☐
I would like to receive a summary of the trial results (it will take up to five years).	☐	☐

For peer review only

Name of parent	Date (dd/mm/yyyy)	Signature
Name of parent	Date (dd/mm/yyyy)	Signature
Name of person taking consent	Date (dd/mm/yyyy)	Signature
Name of interpreter (if applicable)	Date (dd/mm/yyyy)	Signature

2 copies required: Original for Investigator Site File and copy for participant.

#5 BOOSTB4 Consent Confirmation Form Prenatal Trial Group [29-10-2020] v1.1 SE to the
#2 BOOSTB4 Participant Information Prenatal Trial Group [29-10-2020] v1.1 SE

For peer review only - http://bmjopen.bmj.com/site/about/guidelines.xhtml

SPIRIT 2013 Checklist: Recommended items to address in a clinical trial protocol and related documents*

Section/item	Item No	Description	Addressed on page number
**Administrative information**			
Title	1	Descriptive title identifying the study design, population, interventions, and, if applicable, trial acronym	1
Trial registration	2a	Trial identifier and registry name. If not yet registered, name of intended registry	3
	2b	All items from the World Health Organization Trial Registration Data Set	Supplementary material
Protocol version	3	Date and version identifier	Supplementary material
Funding	4	Sources and types of financial, material, and other support	18_
Roles and responsibilities	5a	Names, affiliations, and roles of protocol contributors	1
	5b	Name and contact information for the trial sponsor	1, 9
	5c	Role of study sponsor and funders, if any, in study design; collection, management, analysis, and interpretation of data; writing of the report; and the decision to submit the report for publication, including whether they will have ultimate authority over any of these activities	9, 18-19

For peer review only

	5d	Composition, roles, and responsibilities of the coordinating centre, steering committee, endpoint adjudication committee, data management team, and other individuals or groups overseeing the trial, if applicable (see Item 21a for data monitoring committee)	9
**Introduction**			
Background and rationale	6a	Description of research question and justification for undertaking the trial, including summary of relevant studies (published and unpublished) examining benefits and harms for each intervention	5-8
	6b	Explanation for choice of comparators	Not applicable
Objectives	7	Specific objectives or hypotheses	13
Trial design	8	Description of trial design including type of trial (eg, parallel group, crossover, factorial, single group), allocation ratio, and framework (eg, superiority, equivalence, noninferiority, exploratory)	9
**Methods: Participants, interventions, and outcomes**			
Study setting	9	Description of study settings (eg, community clinic, academic hospital) and list of countries where data will be collected. Reference to where list of study sites can be obtained	9
Eligibility criteria	10	Inclusion and exclusion criteria for participants. If applicable, eligibility criteria for study centres and individuals who will perform the interventions (eg, surgeons, psychotherapists)	Table 1, 23-24
Interventions	11a	Interventions for each group with sufficient detail to allow replication, including how and when they will be administered	11, Figure 1
	11b	Criteria for discontinuing or modifying allocated interventions for a given trial participant (eg, drug dose change in response to harms, participant request, or improving/worsening disease)	11, 12
	11c	Strategies to improve adherence to intervention protocols, and any procedures for monitoring adherence (eg, drug tablet return, laboratory tests)	Not applicable
	11d	Relevant concomitant care and interventions that are permitted or prohibited during the trial	10

For peer review only - http://bmjopen.bmj.com/site/about/guidelines.xhtml

Outcomes	12	Primary, secondary, and other outcomes, including the specific measurement variable (eg, systolic blood pressure), analysis metric (eg, change from baseline, final value, time to event), method of aggregation (eg, median, proportion), and time point for each outcome. Explanation of the clinical relevance of chosen efficacy and harm outcomes is strongly recommended	12
Participant timeline	13	Time schedule of enrolment, interventions (including any run-ins and washouts), assessments, and visits for participants. A schematic diagram is highly recommended (see Figure)	Figure 1, 11-12
Sample size	14	Estimated number of participants needed to achieve study objectives and how it was determined, including clinical and statistical assumptions supporting any sample size calculations	11
Recruitment	15	Strategies for achieving adequate participant enrolment to reach target sample size	11,16

Methods: Assignment of interventions (for controlled trials)

Allocation:

Sequence generation	16a	Method of generating the allocation sequence (eg, computer-generated random numbers), and list of any factors for stratification. To reduce predictability of a random sequence, details of any planned restriction (eg, blocking) should be provided in a separate document that is unavailable to those who enrol participants or assign interventions	11
Allocation concealment mechanism	16b	Mechanism of implementing the allocation sequence (eg, central telephone; sequentially numbered, opaque, sealed envelopes), describing any steps to conceal the sequence until interventions are assigned	Not applicable
Implementation	16c	Who will generate the allocation sequence, who will enrol participants, and who will assign participants to interventions	11
Blinding (masking)	17a	Who will be blinded after assignment to interventions (eg, trial participants, care providers, outcome assessors, data analysts), and how	Not applicable
	17b	If blinded, circumstances under which unblinding is permissible, and procedure for revealing a participant's allocated intervention during the trial	Not applicable

Methods: Data collection, management, and analysis

Data collection methods	18a	Plans for assessment and collection of outcome, baseline, and other trial data, including any related processes to promote data quality (eg, duplicate measurements, training of assessors) and a description of study instruments (eg, questionnaires, laboratory tests) along with their reliability and validity, if known. Reference to where data collection forms can be found, if not in the protocol	12
	18b	Plans to promote participant retention and complete follow-up, including list of any outcome data to be collected for participants who discontinue or deviate from intervention protocols	16
Data management	19	Plans for data entry, coding, security, and storage, including any related processes to promote data quality (eg, double data entry; range checks for data values). Reference to where details of data management procedures can be found, if not in the protocol	9
Statistical methods	20a	Statistical methods for analysing primary and secondary outcomes. Reference to where other details of the statistical analysis plan can be found, if not in the protocol	14
	20b	Methods for any additional analyses (eg, subgroup and adjusted analyses)	Not applicable
	20c	Definition of analysis population relating to protocol non-adherence (eg, as randomised analysis), and any statistical methods to handle missing data (eg, multiple imputation)	14
**Methods: Monitoring**			
Data monitoring	21a	Composition of data monitoring committee (DMC); summary of its role and reporting structure; statement of whether it is independent from the sponsor and competing interests; and reference to where further details about its charter can be found, if not in the protocol. Alternatively, an explanation of why a DMC is not needed	9
	21b	Description of any interim analyses and stopping guidelines, including who will have access to these interim results and make the final decision to terminate the trial	14
Harms	22	Plans for collecting, assessing, reporting, and managing solicited and spontaneously reported adverse events and other unintended effects of trial interventions or trial conduct	12
Auditing	23	Frequency and procedures for auditing trial conduct, if any, and whether the process will be independent from investigators and the sponsor	9

For peer review only - http://bmjopen.bmj.com/site/about/guidelines.xhtml

Ethics and dissemination

Research ethics approval	24	Plans for seeking research ethics committee/institutional review board (REC/IRB) approval	9
Protocol amendments	25	Plans for communicating important protocol modifications (eg, changes to eligibility criteria, outcomes, analyses) to relevant parties (eg, investigators, REC/IRBs, trial participants, trial registries, journals, regulators)	9, 17
Consent or assent	26a	Who will obtain informed consent or assent from potential trial participants or authorised surrogates, and how (see Item 32)	10
	26b	Additional consent provisions for collection and use of participant data and biological specimens in ancillary studies, if applicable	Supplementary material
Confidentiality	27	How personal information about potential and enrolled participants will be collected, shared, and maintained in order to protect confidentiality before, during, and after the trial	Supplementary material
Declaration of interests	28	Financial and other competing interests for principal investigators for the overall trial and each study site	19
Access to data	29	Statement of who will have access to the final trial dataset, and disclosure of contractual agreements that limit such access for investigators	19
Ancillary and post-trial care	30	Provisions, if any, for ancillary and post-trial care, and for compensation to those who suffer harm from trial participation	11
Dissemination policy	31a	Plans for investigators and sponsor to communicate trial results to participants, healthcare professionals, the public, and other relevant groups (eg, via publication, reporting in results databases, or other data sharing arrangements), including any publication restrictions	17
	31b	Authorship eligibility guidelines and any intended use of professional writers	18, Not applicable
	31c	Plans, if any, for granting public access to the full protocol, participant-level dataset, and statistical code	Not applicable

Appendices

For peer review only - http://bmjopen.bmj.com/site/about/guidelines.xhtml

Informed consent materials	32	Model consent form and other related documentation given to participants and authorised surrogates	Supplementary material, 10, 17
Biological specimens	33	Plans for collection, laboratory evaluation, and storage of biological specimens for genetic or molecular analysis in the current trial and for future use in ancillary studies, if applicable	Supplementary material

*It is strongly recommended that this checklist be read in conjunction with the SPIRIT 2013 Explanation & Elaboration for important clarification on the items. Amendments to the protocol should be tracked and dated. The SPIRIT checklist is copyrighted by the SPIRIT Group under the Creative Commons "Attribution-NonCommercial-NoDerivs 3.0 Unported" license.

For peer review only

For peer review only - http://bmjopen.bmj.com/site/about/guidelines.xhtml

BMJ Open

An exploratory open label multicentre phase I/II trial evaluating the safety and efficacy of postnatal or pre- and postnatal administration of allogeneic expanded fetal mesenchymal stem cells for the treatment of severe Osteogenesis Imperfecta in infants and fetuses: The BOOSTB4 Trial Protocol

Journal:	*BMJ Open*
Manuscript ID	bmjopen-2023-079767.R2
Article Type:	Protocol
Date Submitted by the Author:	08-Mar-2024

Complete List of Authors:	Sagar, Rachel; University College London, Elizabeth Garrett Anderson Institute for Women's Health ; NIHR University College London Hospitals Biomedical Research Centre<br>Åström, Eva; Karolinska Institutet, Department of Women's and Children Health; Karolinska University Hospital, Astrid Lindgren Children's Hospital<br>Chitty, Lyn; Great Ormond Street Hospital for Children NHS Foundation Trust, North Thames Genomic Laboratory Hub; UCL Great Ormond Street Institute of Child Health, Genetics and Genomics<br>Crowe, Belinda; Great Ormond Street Hospital for Children NHS Foundation Trust, Department of Neurosciences<br>David, Anna; University College London, Elizabeth Garrett Anderson Institute for Women's Health; NIHR University College London Hospitals Biomedical Research Centre<br>DeVile, Catherine; Great Ormond Street Hospital for Children NHS Foundation Trust, Department of Neurosciences<br>Forsmark, Annabelle ; PharmaLex<br>Franzen, Vera; XNK Therapeutics AB<br>Hermeren, Göran; Lund University Faculty of Medicine, Department of Clinical Sciences<br>Hill, Melissa; Great Ormond Street Hospital For Children NHS Foundation Trust, North Thames Genomic Laboratory Hub; UCL Great Ormond Street Institute of Child Health, Genetics and Genomics<br>johansson, mats; Lund University Faculty of Medicine, Department of Clinical Sciences<br>Lindemans, Caroline; University Medical Centre Utrecht, Department of Pediatrics,<br>Lindgren, Peter; Karolinska University Hospital, Center for Fetal Medicine; Karolinska Institutet, Department of Clinical Science, Intervention and Technology<br>Nijhuis, Wouter; University Medical Centre Utrecht, Department of Orthopedic Surgery<br>Oepkes, Dick; Leiden University Medical Center, Department of Obstetrics<br>Rehberg, Mirko; University Hospital Cologne, Department of Pediatrics<br>Sahlin, Nils-Eric; Lund University Faculty of Medicine, Department of Clinical Sciences

	Sakkers, Ralph; University Medical Centre Utrecht, Department of Orthopedic Surgery<br>Semler, O.; University Hospital Cologne, Department of Pediatrics<br>Sundin, Mikael; Karolinska Institutet, Department of Clinical Science, Intervention and Technology; Karolinska University Hospital, Section of Pediatic Hematology, Immunology and HCT<br>Walther-Jallow, Lilian; Karolinska Institutet, Department of Clinical Science, Intervention and Technology<br>Verweij, E.J.T; Leiden University Medical Center, Department of Obstetrics<br>Westgren, Magnus; Karolinska Institute, Department of Clinical Science, Intervention and Technology<br>Götherström, Cecilia; Karolinska Institute, Department of Clinical Science, Intervention and Technology
**Primary Subject Heading**:	Paediatrics
Secondary Subject Heading:	Obstetrics and gynaecology, Research methods
Keywords:	Mesenchymal Stem Cells, Cell biology < NATURAL SCIENCE DISCIPLINES, Fetal medicine < OBSTETRICS, Musculoskeletal disorders < ORTHOPAEDIC & TRAUMA SURGERY, Clinical trials < THERAPEUTICS, TRANSPLANT MEDICINE

SCHOLARONE™
Manuscripts

An exploratory open label multicentre phase I/II trial evaluating the safety and efficacy of postnatal or pre- and postnatal administration of allogeneic expanded fetal mesenchymal stem cells for the treatment of severe Osteogenesis Imperfecta in infants and fetuses: The BOOSTB4 Trial Protocol

Rachel L Sagar (rachelsagar@nhs.net),[1,2]* Eva Åström (eva.astrom@regionstockholm.se),[3,4]* Lyn S Chitty (l.chitty@ucl.ac.uk),[5,6] Belinda Crowe (Belinda.Crowe@gosh.nhs.uk),[7] Anna L David (a.david@ucl.ac.uk),[1,2] Catherine DeVile (Catherine.DeVile@gosh.nhs.uk),[7] Annabelle Forsmark (annabelle.forsmark@pharmalex.com),[8] Vera Franzén (vera.frz@telia.com),[9] Göran Hermerén (goran.hermeren@med.lu.se),[10] Melissa Hill (melissa.hill@ucl.ac.uk),[5,6] Mats Johansson (mats.johansson@med.lu.se),[10] Caroline A Lindemans (C.A.Lindemans@prinsesmaximacentrum.nl),[11] Peter Lindgren (peter.lindgren.1@ki.se),[12,18] Wouter H Nijhuis (W.H.Nijhuis-2@umcutrecht.nl),[13] Dick Oepkes (D.Oepkes@lumc.nl),[14] Mirko Rehberg (mirko.rehberg@uk-koeln.de),[15] Nils-Eric Sahlin (nils-eric.sahlin@med.lu.se),[10] Ralph Sakkers (Sakkers@umcutrecht.nl),[13] Oliver Semler (joerg.semler@uk-koeln.de),[15] Mikael Sundin (mikael.sundin@ki.se),[16,17] Lilian Walther-Jallow (lilian.walther.jallow@ki.se),[18] E.J.T. (Joanne) Verweij (e.j.t.verweij@lumc.nl),[14] Magnus Westgren (magnus.westgren@ki.se)[18] and Cecilia Götherström (cecilia.gotherstrom@ki.se)[18]

*Shared first authorship.

[1]Elizabeth Garrett Anderson Institute for Women's Health, University College London, London, United Kingdom.

[2]NIHR University College London Hospitals Biomedical Research Centre, London, United Kingdom.

[3]Neuropediatric Unit, Astrid Lindgren Children's Hospital, Karolinska University Hospital, Stockholm, Sweden.

[4]Neuropediatric Unit, Department of Women's and Children Health, Karolinska Institutet, Stockholm, Sweden.

[5]North Thames Genomic Laboratory Hub, Great Ormond Street NHS Foundation Trust, London, United Kingdom.

[6]Genetics and Genomics, UCL Great Ormond Street Institute of Child Health, London, United Kingdom.

[7]Department of Neurosciences, Great Ormond Street Hospital for Children NHS Foundation Trust, London, United Kingdom.

[8]PharmaLex, Gothenburg, Sweden.

For peer review only

For peer review only - http://bmjopen.bmj.com/site/about/guidelines.xhtml

[9]XNK Therapeutics AB, Huddinge, Sweden.

[10]Medical Ethics, Department of Clinical Sciences, Lund, Faculty of Medicine, Lund University, Lund, Sweden.

[11]Department of Pediatrics, University Medical Center Utrecht, Utrecht, The Netherlands.

[12]Center for Fetal Medicine, Karolinska University Hospital, Stockholm, Sweden.

[13]Department of Orthopedic Surgery, University Medical Centre Utrecht, Wilhelmina Children's Hospital, Utrecht, The Netherlands.

[14]Department of Obstetrics, Division of Fetal Therapy, Leiden University Medical Center, Leiden, The Netherlands.

[15]Department of Pediatrics, Faculty of Medicine, University of Cologne and University Hospital Cologne, Cologne, Germany.

[16]Division of Pediatrics, Department of Clinical Science, Intervention and Technology, Karolinska Institutet, Stockholm, Sweden.

[17]Section of Pediatic Hematology, Immunology and HCT, Astrid Lindgren Children's Hospital, Karolinska University Hospital, Stockholm, Sweden.

[18]Division of Obstetrics and Gynecology, Department of Clinical Science, Intervention and Technology, Karolinska Institutet, Stockholm, Sweden.

Corresponding author and Sponsor's contact information: Cecilia Götherström, Division of Obstetrics and Gynecology, Department of Clinical Science, Intervention and Technology, Karolinska Institutet, ANA Futura, Alfred Nobels Allé 8, 141 52 Huddinge, Sweden. Phone +4670-4712300

Keywords: Osteogenesis Imperfecta; Brittle Bone Disease; Mesenchymal Stem Cells; Transplantation; Clinical Trial.

Word count: 5082 (not including the title page, abstract, references, statements, figures, tables or acknowledgments.)

For peer review only

ABSTRACT

Introduction: Severe Osteogenesis Imperfecta (OI) is a debilitating disease with no cure or sufficiently effective treatment. Mesenchymal stem cells (MSCs) have good safety profile, show promising effects and can form bone. The Boost Brittle Bones Before Birth (BOOSTB4) trial evaluates administration of allogeneic expanded human first trimester fetal liver MSCs (BOOST cells) for OI type 3 or severe type 4.

Methods and analysis: BOOSTB4 is an exploratory, open label, multiple dose, phase I/II clinical trial evaluating safety and efficacy of postnatal (n=15) or prenatal and postnatal (n=3, originally n=15) administration of BOOST cells for the treatment of severe OI compared with a combination of historical (1–5/subject) and untreated prospective controls (≤30). Infants <18 months of age (originally <12 months) and singleton pregnant women whose fetus has severe OI with confirmed glycine substitution in *COL1A1* or *COL1A2* can be included in the trial.

Each subject receives four intravenous doses of $3x10^6$/kg BOOST cells at 4-month intervals, with 48 (dose 1–2) or 24 (dose 3–4) hours in-patient follow-up, primary follow-up at 6 and 12 months after the last dose, and long-term follow-up yearly until 10 years after the first dose. Prenatal subjects receive the 1st dose via ultrasound-guided injection into the umbilical vein within the fetal liver (16+0 to 35+6 weeks), and three doses postnatally.

The primary outcome measures are safety and tolerability of repeated BOOST cell administration. The secondary outcome measures are number of fractures from baseline to primary and long-term follow-up, growth, change in bone mineral density, clinical OI status and biochemical bone turnover.

Ethics and dissemination: The trial is approved by Competent Authorities in Sweden, the United Kingdom and the Netherlands (postnatal only). Results from the trial will be disseminated via CTIS, ClinicalTrials.gov and in scientific open access scientific journals.

For peer review only

For peer review only - http://bmjopen.bmj.com/site/about/guidelines.xhtml

Trial registration numbers: EudraCT 2015-003699-60, EUCT: 2023-504593-38-00, ClinicalTrials.gov NCT03706482; Pre-results.

Strengths and limitations of this study:

- This is a protocol for an open label clinical trial; which means that there is no randomisation and the treatment is known both to the families and the trial personnel.
- OI is by nature a heterogenous and variable disease, which may impact the possibility to satisfactorily evaluate the effect of treatments. To address this and collect reliable data, the BOOSTB4 trial collects data longitudinally and includes multiple follow-up assessments of each subject.
- The trial may be underpowered to show improvements in efficacy. Therefore, if the trial yields a positive result, a larger subsequent multi-centre pivotal trial may be needed.

For peer review only

INTRODUCTION

Osteogenesis imperfecta (OI) is a rare clinically and genetically heterogenous group of heritable disorders of connective tissue characterised by osteopenia, growth restriction, bone deformities, fractures and chronic pain.(1) Originally, OI was classified into four types according to clinical, radiological and histological findings, where type 1 refers to the mildest form, type 2 to the perinatally lethal form, type 3 to the most severe form compatible with life, and type 4 to an intermediate group with moderate to severe types of OI.(2) This classification covers 85–90% of persons with OI and includes >1,500 different autosomal dominant sequence variations in the genes coding for type I collagen (*COL1A1* or *COL1A2*).(1-3) There is currently no curative treatment for OI, with the only available pharmaceutical treatments failing to address the underlying molecular pathology, the collagen defect.(4) Treatment is aimed at increasing overall bone strength to prevent fractures and maintain mobility. Bisphosphonates are used to reduce bone resorption and increase bone mass.(5) However, two meta-analyses of randomised trials on bisphosphonate treatment for OI did not demonstrate improvement in fracture rates, reduction of pain or improved functional mobility, although multiple studies report this independently.(6 7)

One child among 10–20,000 is born with OI. Diagnosis of severe types of OI is often made at the routine fetal anomaly ultrasound scan mid-pregnancy, or earlier if first trimester scanning is routine. Early treatment of OI is preferred to minimise irreversible pathology. Prenatal intervention offers multiple potential advantages over postnatal approaches; ameliorating and preventing damage that has begun already during fetal life, the relatively naive fetal immune system may permit the development of immune tolerance towards donor cells, infusion into the systemic fetal circulation may avoid sequestration of the cells in the lungs because circulatory shunts allow the pulmonary circulation to be bypassed, fetal life is a time of natural stem cell proliferation and migration to different anatomic compartments. Also, prenatal administration may enhance the effect of MSCs because there will be a lesser amount of poor quality OI type collagen that requires replacement, and

For peer review only - http://bmjopen.bmj.com/site/about/guidelines.xhtml

lastly, the possible psychological benefits for parents of commencing treatment as early in the child's life.(8)

The investigational medicinal product (IMP) investigated in the BOOSTB4 trial is an advanced therapy medicinal product (ATMP) consisting of allogeneic *ex vivo* expanded human fetal liver-derived mesenchymal stem cells (MSCs) from the first trimester of pregnancy. MSCs are multipotent stem cells and are promising candidates in regenerative medicine due to their low immunogenic profile that allows the possibility of performing cell transplantation across major histocompatibility barriers without immunosuppression.(9 10) In addition, they have the potential to differentiate along the osteogenic, chondrogenic and adipogenic lineages and have a good safety profile with minimal oncogenic risk.(11-16) Two meta-analyses of eight and 55 randomised controlled trials investigating intravascular administration of naïve MSCs to 321 and 2696 subjects, respectively did not detect an association between non-fever acute infusional toxicity, organ system complications, thrombotic/embolic events, infection, death or malignancy.(17 18) The only significant association with intravascular administration of MSCs was a transient fever.(17 18) The first clinical infusion of MSCs was performed over 20 years ago and adult MSCs have since been used in a diverse range of conditions in thousands of patients.(9 17) Studies show that MSCs derived from developmentally younger donor sources present characteristics suggesting a stronger cell therapeutic potential than adult MSCs, and this knowledge is being translated into clinical application. First trimester fetal MSCs are found at a higher frequency, are less mature on the transcriptomic level, have a greater colony-forming and proliferation capacity and are more immune modulatory compared to adult MSCs.(10 19-22) Interestingly, fetal MSCs differentiate into more lineages than MSCs derived from adult tissues, like skeletal muscle, oligodendrocytes and hepatic cells,(23-25) and differentiate more readily into bone than adult MSCs. In a direct comparison of fetal MSCs and MSC derived from Wharton's Jelly, adult bone marrow and adipose tissue, fetal MSCs had higher levels of osteogenic genes under basal conditions.(26 27) Upon osteogenic differentiation, fetal MSCs produced more robust osteogenic gene expression, induced more calcium deposition *in vitro* and reached higher levels of osteogenic

For peer review only - http://bmjopen.bmj.com/site/about/guidelines.xhtml

gene upregulation *in vitro* and *in vivo*.(26-28) First trimester fetal liver MSC were selected for use in this trial given their superior osteogenic differentiation capacity, their safety profile and the feasibility of manufacturing a high quality GMP-compliant off-the-shelf product with retained efficacy.

The potential of MSC transplantation for the treatment of OI has been shown in different mouse models of OI, with demonstrated homing and engraftment in bone, increased collagen content and mineralisation of the bones, improved bone strength, thickness and length, and 67–84% reduction in long bone fractures.(29-31) Clinically, two studies on intravenous administration of histocompatible MSCs from sibling donors to children with severe OI have been performed (6 and 2 children treated respectively, where 6 children underwent myeloablative haematopoietic stem cell transplantation before they received MSCs).(32-35) The MSC administration procedures were safe with no serious adverse events (SAEs) reported, and positive clinical effects were attained with donor cell engraftment, development of new compact bone, improved bone parameters, and six months following two MSC infusions there was an increase in growth velocity, an 80% reduction in fracture frequency, an increase in total bone mineral content in 3/8 of the children (34 35) and in Quality of Life.(35 36)

We have previously reported two case studies of prenatal and postnatal transplantation of fetal MSCs for severe OI (type 3 and type 4 OI).(37 38) The limited data show that the procedures were safe and clinically promising when comparing the clinical course to other individuals with identical OI sequence variants. Only three other individuals in the world are currently known to have a *COL1A2* variant identical to that of the patient with OI type 3; one of whom died from the severe type of OI despite bisphosphonate treatment, and two individuals are described as having type 2/3 OI.(39 40) After one dose of fetal MSCs prenatally, donor cells were found in the recipient bones at nine months of age, and the donor cells expressed bone markers.(37) Four booster doses with same donor MSCs between 8–13 years of age show a reduction in fracture rate (from 2/year to 0/year over two years), and an increase in height, albeit 5.0–6.4 standard deviations below the mean.(38) While it is difficult to

For peer review only

For peer review only - http://bmjopen.bmj.com/site/about/guidelines.xhtml

determine the definitive impact of the prenatal and postnatal MSC transplantation in these two heterogeneous cases, the findings suggest a potential clinical benefit. The children have now been followed up for over 21 and 15 years, respectively, and there has been no evidence of any early or late adverse reactions.

Donor cell engraftment in bones has been reported to be between 0 to 16.4% after stem cell transplantation in both human cases of OI and mouse models.(29-34 37 38) It is difficult to determine if such low levels of engraftment could result in adequate synthesis of healthy type I collagen and significant clinical improvement. However, Panaroni et al. reported that *in utero* transplantation of healthy bone marrow to the Brtl OI mouse resulted in donor cell engraftment of 1–2% in the bones, which in turn resulted in a 20% production of healthy collagen in the host bones.(30) The transplantation eliminated the perinatal lethality of Brtl mice, and furthermore the femora of treated Brtl mice had significant improvement in geometric parameters compared to Brtl mice and in mechanical properties compared to wild-type mice. Hence, low level engraftment of cells producing healthy collagen may lead to significant improvements in bone development.

Transplantation of MSCs is not a cure, but it is currently the only therapy available in clinical phase trials for OI that may result in healthy bone cells and production of healthy collagen. Other treatments developed for OI are based on modifying the action of the patient's own cells, and hence can only result in increased quantity of bone rather than improved quality of bone. However, the clinical effects of MSC administration may not be persistent and therefore re-dosing may be required. The interval for re-dosing is currently not known.

There is increasing interest in treatment with MSCs and a growing number of requests from patients and their physicians to provide it. In the absence of a clinical trial, prenatal and postnatal MSC transplantation for OI is likely to be practised around the world on a case-by-case basis, making it impossible to evaluate any effects the intervention might have. Considering the complexity of this field and the distribution of cases, an international trial is the only realistic and ethically correct

For peer review only - http://bmjopen.bmj.com/site/about/guidelines.xhtml

approach to enable accurate evaluation of the intervention. BOOSTB4 is the first clinical trial that will assess whether postnatal or pre- and postnatal repeated intravenous administration of MSCs is a safe and potentially effective treatment option for severe OI. The BOOSTB4 trial will also provide a well-defined framework for assessment of children and fetuses with OI treated with fetal MSCs.

For peer review only

For peer review only - http://bmjopen.bmj.com/site/about/guidelines.xhtml

METHODS AND ANALYSIS

Trial design

Boost Brittle Bones Before Birth (BOOSTB4) is an exploratory, open label, multicentre, phase I/II trial evaluating safety and efficacy of four postnatal intravenous doses (n=15 infants) or one prenatal and three postnatal intravenous doses (n=3 fetuses in the amended protocol, in the original protocol it was n=15 fetuses) of allogeneic expanded fetal MSCs (BOOST cells) for the treatment of severe OI compared with a combination of historical (1–5/subject) and up to 30 untreated prospective controls. Please see the trial synopsis in Supplementary material.

Setting

The Sponsor for this academic trial is Karolinska Institutet (coordinator) in Sweden. The trial sites are five maternity/fetal medicine and paediatric institutions; Karolinska University Hospital in Sweden (coordinating trial centre, OI and maternal/fetal medicine, manufacturer of the IMP), University College London (trial centre, maternal/fetal medicine) and Great Ormond Street Hospital for Children (trial centre, OI) in the United Kingdom, and Leiden University Medical Center (trial centre, maternal/fetal medicine) and University Medical Center Utrecht (trial centre, OI) in the Netherlands.

Investigational Medicinal Product

The IMP BOOST cells consists of cryopreserved expanded allogenic human first trimester liver-derived MSCs (the liver is the blood-forming organ at this time of development) obtained from early elective surgical terminations of pregnancy (see the Ethics and Dissemination section for information on donor information and consent). The IMP is manufactured under Good Manufacturing Practice (GMP), and the cells are isolated, expanded up to passage 4 and then cryopreserved as an off-the-shelf product ready to be thawed and reconstituted prior to administration. The cells are tested according to the Product Specification for sterility, endotoxin and mycoplasma with methods conforming to Pharmacopeia, and they are further tested for expression of MSC markers by flow cytometry and for efficacy by a bone differentiation assay. The Mechanism of Action of the IMP is engraftment and bone

For peer review only

For peer review only - http://bmjopen.bmj.com/site/about/guidelines.xhtml

differentiation. Two donors are used in the BOOSTB4 trial. At least 80 clinical doses of MSCs, and MSCs for retention samples and all safety and functionality analyses, will be manufactured from two donors.

Eligibility and recruitment

The inclusion and exclusion criteria for the three trial groups are described in Table 1. The upper age limit was amended from maximum 12 months of age to 18 months of age in the postnatal group. This change to the original protocol has been approved by the Competent Authorities, and was implemented to allow for inclusion of more subjects to the postnatal group. All subjects (the postnatal, prenatal and control groups) will receive bisphosphonate treatment. At least one dose of bisphosphonate must be administered before the first dose of stem cells in the postnatal group. In the prenatal group the first dose of bisphosphonates will be administered after birth. Acceptable types of concomitant medication including bisphosphates during the trial and until the 12-month follow-up are Pamidronate (preferred) or Neridronate, and the annual dose of bisphosphate is 8–12 mg/kg body weight per year.

Information about the BOOSTB4 trial has been distributed to specialist OI services and fetal medicine units in Europe, and the BOOSTB4 trial team can also be contacted directly by families or physicians via email or the trial specific website www.boostb4.eu where the Patient Information Leaflet is available in several languages. The potential subjects are invited to visit the trial site or have an organized video meeting and provide their oral and written consent to be screened for participation. At least three days will be required for the parents/legal guardians to consider participation in the trial. For all children enrolled in the trial, a signed informed-consent form for the main trial is required from the mother and the father, if she/he has parental responsibility (see Supplementary material for the screening and main trial participation consent forms for the postnatal and prenatal groups, and www.boostb4.eu for the Participant information sheets). For prenatal inclusion, only the pregnant woman needs to sign the informed-consent form, but both parents are encouraged to sign. After the birth of a child in the prenatal group the inclusion and exclusion criteria for the postnatal group must

For peer review only - http://bmjopen.bmj.com/site/about/guidelines.xhtml

be fulfilled (Table 1), and both parents must re-consent their participation in the trial before the first postnatal dose. Consent may be withdrawn at any time without a reason being stated. The Sponsor will hold clinical trial insurance that covers all enrolled subjects receiving active treatment.

Sample size calculation

No formal power calculations has been performed. OI is a rare disease, and a limited number of subjects can be included each year. Based on the incidence, it was originally reasonable to include 30 patients (15 postnatal subjects and 15 prenatal subjects) in this first trial on the grounds of feasibility, suitable time period and for the application of descriptive statistical analyses. However, in an amendment to the original protocol in 2023, which is approved by the Competent Authorities, the number of subjects in the prenatal group was reduced from 15 to three. This change was requested by the Sponsor due to time restrictions in the academic funding of the trial. The risk – benefit balance for participation in any of the trial groups was not changed. Since the purpose of the prenatal trial group was to demonstrate feasibility of administration of one dose of BOOST cells before birth and three doses after birth, this change to the original protocol is not considered to alter the outcome of the trial's objectives.

Blinding, intervention and follow-up

It would not be ethically appropriate to perform a randomised double-blind placebo-control trial in this population, particularly regarding the risks of the prenatal infusion procedure and the vulnerability of the subjects. No blinding or randomisation will therefore take place.

Four intravenous doses of BOOST cells are administered at four-month intervals (range 3–5 months), please see Figure 1 for an overview of the trial. For postnatal subjects the 1st dose must be administered before 18 months of age (in the original protocol it was before 12 months of age; an amendment that has been approved by the Competent Authorities). For prenatal subjects, the 1st dose will be given *in utero* during gestational weeks+days 16+0–35+6 via ultrasound-guided injection into the umbilical vein within the fetal liver, and the final three doses are administered postnatally. Each

For peer review only - http://bmjopen.bmj.com/site/about/guidelines.xhtml

dose contains $3x10^6$ allogeneic expanded human fetal liver MSCs per kg bodyweight. All doses to a subject will be derived from one donor. No pre-conditioning or medicines after the administration will be administered.

In-patient follow-up is performed over 48 (dose 1–2) or 24 (dose 3–4) hours in the postnatal group and over 48 (dose 1–3) or 24 (dose 4) hours in the prenatal group after each dose. Primary follow-up is performed at 6 and 12 months after the last dose, and long-term follow up is performed yearly until 10 years after the first dose. At each visit and follow-up, AEs and parameters related to efficacy outcomes are recorded. Women in the prenatal group are followed under the same schedule.

Prospective controls will be subject to the same follow-up routine as the patients enrolled in the treatment group, as described above, with the option to opt in or out from blood collection. The clinical evaluation program for the historical control group will be based on data available from the routine follow-up of OI patients in the respective country. The controls will be individually matched to each subject (see Table 2). Between one and five historical controls will be included per subject and up to 30 prospective controls in total.

Outcome measures

The primary endpoint of the BOOSTB4 trial is safety and tolerability measured as seriousness, severity and frequency of administration-related adverse events (AEs) in the child, fetus and woman after four postnatal or one prenatal and three postnatal intravenous doses of BOOST cells every four months. Specific focus will be given to vital signs, transfusion and immune reactions and maternal and fetal AEs using the Maternal and Fetal AE Terminology,(41) for example, persistent fetal bradycardia following the infusion, tumorigenicity, morbidity and mortality. The trial has clearly defined Stopping criteria on trial, group and subject levels. In case of a serious adverse reaction, the subject may not receive more doses of BOOST cells. The Data and Safety Monitoring Board will review all AEs and adverse reactions.

For peer review only - http://bmjopen.bmj.com/site/about/guidelines.xhtml

Vital signs (oxygen saturation, heart rate, respiratory rate, body temperature, blood pressure [only in the pregnant woman], medical examination and signs of suspected pulmonary embolism) are monitored in the child and pregnant woman before, during and until 48 hours (dose 1–2) or 24 hours (dose 3–4) after each dose. In the fetus the following is monitored before, during and until 48 hours after the prenatal dose using ultrasound: fetal heart rate, middle cerebral artery pulsatility index, peak systolic velocity, umbilical artery Doppler pulsatility index and cardiotocography.

The following parameters are analysed in peripheral blood before, and at 1, 24 (dose 1–4) and 48 hours (dose 1–2) after each dose (does not apply to the fetus): blood status (Haemoglobin, Haematocrit, Leukocytes, Thrombocytes, Erythrocytes, Mean corpuscular volume), liver (ALAT and ASAT), kidney (Creatinine), Electrolytes (Chloride, Magnesium, Sodium, Potassium). Donor specific antibodies are analysed before the first dose and >3 weeks after each dose.

The secondary endpoints relate to efficacy of four doses of BOOST cells in children with OI. The hypothesis under investigation is that early administration of BOOST cells will ameliorate severe types of OI. The key secondary endpoint is number of fractures from baseline to primary (12 months after the last dose) and long-term (10 years after the first dose) follow-up. Fracture frequency was chosen as the key secondary endpoint because it is the most clinically relevant efficacy variable. Additional secondary endpoints are time to first fracture after each BOOST cell administration, number of fractures at birth in the prenatal group, growth (length/height and weight), change in bone mineral density measured with DXA/DEXA (not a trial specific investigation, optional and according to the national follow-up program), clinical OI status and biochemical bone turnover in peripheral blood. The analysis of biochemical bone turnover includes Calcium, Phosphate, Albumin, Parathyroid hormone, 25-OH Vitamin D, Alkaline phosphatase, Bone specific Alkaline phosphatase, Osteocalcin and cross-linked C-telopeptide of type I collagen, and will be carried out before each dose and then according to the clinical routine every 6–12 months. No radiological measurements will be performed specifically for the trial.

For peer review only - http://bmjopen.bmj.com/site/about/guidelines.xhtml

The exploratory endpoints investigated by the trial include assessing the impact of the therapy on Quality of Life using the Infant Toddler Quality of Life Questionnaire™ (ITQOL), collection of peripheral blood before and at 1 and 24h (dose 1–4) and 48h (dose 1–2) after each dose for studying the paracrine effect (via the Olink technology or ELISA) and the effect on endogenous immune cells (via flow cytometry) of BOOST cells in subjects and prospective controls (blood sampling optional). The extent of donor cell engraftment in tissue samples collected from routine surgery will be studied, and a method for non-invasive method for prenatal diagnosis of OI developed (not used as the diagnostic method in the trial). In addition, there will be a sub-study to BOOSTB4, where parents will have the option to take part in two semi-structured interviews, in which their experiences of their participation in the trial are explored. This will allow us to understand their reasoning for their child to participate in the trial, and what has been positive and what can be improved in future similar studies.

Statistical analysis

The primary endpoint of the BOOSTB4 trial is the safety of postnatal or prenatal and postnatal administration of BOOST cells. It involves investigation of the seriousness, severity and frequency of treatment-related AEs in the child, woman and fetus. All reported AEs (regardless of the cause) will be included in the statistical analysis, and all analyses will be performed separately for the infant/child, fetus and pregnant woman. No imputation of missing data will take place.

The BOOSTB4 trial is a non-randomised trial and specific caution will be taken to control for problems with bias. The statistical analysis will include descriptive statistics of the data that will be discussed in relation to the general knowledge of the condition, to generate an overall picture of the safety and efficacy profile of the treatment. All trial subjects will be compared to himself or herself as his or her own control over time (longitudinally from baseline to the primary follow-up at 12 months after the last dose and to the long-term follow-up until 10 years after the first dose). In the analysis of parameters such as blood status and biochemical bone turnover, non-parametric Wilcoxon matched pairs signed-rank test (before versus after doses and over time) of will be used. One to five matched

For peer review only - http://bmjopen.bmj.com/site/about/guidelines.xhtml
For peer review only

historical controls (18–90 in total) will, as far as possible, be included per trial subject to increase the statistical precision, and up to 30 prospective untreated controls will also be included.

Patient and public involvement

The trial has been discussed with patient support groups including the OI Federation Europe (OIFE) and Care 4 Brittle Bones (C4BB) from early in the planning stage. We are grateful for their input, and for the joint dissemination of the trial in 2019.

In a separate study we explored stakeholder views to understand perceived benefits or concerns, identify ethical issues and establish protocols for support and counselling. Semi-structured qualitative interviews were conducted with 56 participants from three groups: adults affected with OI, with and without children, and parents of children affected with OI, health professionals who work with patients with OI, and patient advocates from relevant patient support groups.(8) There were generally positive views towards using fetal MSC transplantation to treat OI, with early treatment considered to be advantageous for preventing fractures and reducing severity, as well as potentially bringing psychological benefits for parents. Participants were somewhat concerned about procedure safety, short and long-term side effects and the effectiveness of transplantation. The difficulties inherent in decision-making were frequently raised, as well as the importance of explaining fetal MSC transplantation in a way that all parents can understand, setting clear expectations, providing psychological support and allowing time to reflect during the decision-making process. The feedback received has been incorporated into the design and planning of the BOOSTB4 trial.

Health economic assessment

A health economic assessment is being undertaken with the objective to describe the costs and effects of BOOST cell administration as a potential standard treatment for severe forms of OI. Published data on the impact of OI on healthcare burden are scarce,(42) with available studies reporting a high cost for e.g. frequent hospital admissions.(43 44) Also, two sets of questionnaires have been developed to capture dependency on healthcare and/or community services, with one focusing on patient/family

For peer review only - http://bmjopen.bmj.com/site/about/guidelines.xhtml

(postnatal and prenatal groups) and the other on maternal health (prenatal group). These questionnaires will be used to further assess the costs and effects of BOOST cell transplantation for severe forms of OI, in addition to further exploring the economic burden of OI overall.

Trial status

Recruitment commenced in Sweden after the site opened on 12th of August 2019. The first subject in the postnatal group was included and received the 1st dose on 17th of March 2020. Due to the SARS-CoV-2 pandemic only two subjects were included in the trial during 2020, and due to time restrictions for the project, the dose administrations are only performed at Karolinska University Hospital in Sweden. After a safety assessment by the Data and Safety Monitoring Board of at least one dose administered to the 1st five postnatal subjects in the trial, the prenatal group of the trial was opened 17th of June 2021, and the 1st prenatal dose was administered on 18th of November 2021. By 2nd of March 2022, all 15 of 15 subjects in the postnatal group had been included and received their 1st dose, and by 14th of December 2021, three of 15 prenatal subjects had been included and administered with one prenatal dose each. No more subjects will be included in the prenatal group due to time restrictions in funding of the trial (an amendment approved by the Competent Authorities). The primary follow-up is currently being performed and is expected to be completed in January 2024, and the long-term follow-up will be completed in 2032.

ETHICS AND DISSEMINATION

The BOOSTB4 clinical trial aims to investigate BOOST cell treatment in young children and unborn children (which includes the pregnant woman as a research subject) suffering from a chronic disease. The trial has obtained ethical and regulatory approval from the national Competent Authorities in Sweden (the Swedish Ethical Review Authority (Etikprövningmyndigheten), approval no 2018/1732-31 and the Swedish Medical Product Agency, approval no 5.1-2018-57411), United Kingdom (the North East – York Research Ethics committee, approval no 19/NE/0030 and the UK Medicines & Healthcare products Regulatory Agency, approval no 44119/0002/001-001), Netherlands (the Central

For peer review only - http://bmjopen.bmj.com/site/about/guidelines.xhtml

Committee on Research Involving Human Subjects [Centrala Commissie Mensgebonden Onderzoek], approval no NL64105.000-17, postnatal part). The NHS Research Ethics Committee the North of Scotland Research Ethics Committee separately approved the interview study in the United Kingdom (approval no 16/NS/0084), which were part of the approved trial protocol in Sweden and the Netherlands. Amendments have been implemented after approval from the National Competent Authorities. An approved amendment included reducing the prenatal trial group from 15 subjects to three subjects (the Swedish Ethical Review Authority, approval no 2023-03725-02 and the Swedish Medical Product Agency, approval no 5.1-2023-38290). Oral and written informed consent will be obtained from the legal guardians to all subjects/the pregnant women (for prenatal inclusion), please refer to the section Eligibility and recruitment for details. The trial is performed according to Good Clinical Practice (GCP). The data is entered into electronic Case Report Forms, and the data and trial are monitored according to the trial's Monitoring Plan. The conduct of the trial is overseen by an independent Data and Safety Monitoring Board according to their Charter. The Data and Safety Monitoring Board includes expertise on OI, haematology, transplantation and fetal and maternal medicine.

Several ethical issues arise when considering stem cell transplantation for OI in these study populations. Therefore, a detailed and systematic risk – benefit assessment was performed before initiation of the trial, which is updated when needed, for example during the COVID pandemic. The assessment considers many areas; risks related to handling, administration and exposure to the IMP, and risks for the child, fetus and pregnant woman. The assessment also includes potential benefits for the child, fetus and the parents. All risks and benefits are discussed in relation to the Sponsor's experience with administration of fetal MSCs for treatment of OI. In other words, this therapy is believed to be directly beneficial to the fetus and child, but there are also significant unknowns.

In the BOOSTB4 trial there have been several separate considerations of diverse ethical issues. They include reviewing the ethical and legal standards relevant to prenatal and postnatal stem cell

For peer review only

For peer review only - http://bmjopen.bmj.com/site/about/guidelines.xhtml

transplantation, mapping the rights and interests of the research subjects, investigating stakeholder's views,(8) as well as testing the Patient Information Leaflet and the communication of risks.

The fetal MSCs used for manufacturing the IMP BOOST cells are obtained from first trimester fetal tissues after elective termination of pregnancy. Ethical approval has been obtained in Sweden for the use of fetal tissues for manufacture of the IMP (the Swedish Ethical Review Authority, approval no 2018/1732-31 and the National Board of Health and Welfare, approval no 8.1-28985/2018). The donating woman independently seeks medical care. Oral and written information about the study is given by a medical practitioner not related to the study, who also obtains informed oral and written consent. A health declaration and infectious screening is performed. The woman can withdraw from the study until the IMP has been manufactured.

Fetal tissues might pose an issue with regards to ethical as well as religious beliefs for some individuals, and transparency in this is vital. To address this, we performed interviews with 56 participants as explained above under Patient and public involvement. The interviews revealed generally positive views towards using fetal MSCs for treating severe OI.(8)

Identifying the main stakeholders is fundamental since the safety and rights of the research subjects should be the main concern. At the prenatal stage the main stakeholders are the pregnant woman and the fetus. At the postnatal stage, only the minor is the research subject, but the parents are also stakeholders, since the infant cannot consent to the participation. We have also discussed the risk of overprotection of pregnant women since they are considered to be a vulnerable population. This may violate their autonomy, harm their interests, and prevent research that could help children and possibly improve their own Quality of Life. Overprotection may signal good intentions and an ambition to minimise risks, but it could also result from a tendency to underestimate the benefits the therapy may bring to the women from whom consent is being sought. Partner consent is not legally required prenatally, although there are exceptions (discussed in (45)). Nonetheless, it is recommended if the

For peer review only - http://bmjopen.bmj.com/site/about/guidelines.xhtml

partner is expected to become the child's legal representative after birth. It will also be impossible to complete the trial if the partner dissents after birth, and the trial concerns the partner in many ways.

As a sub-study to BOOSTB4, the parents can consent to take part in two qualitative interviews. This will allow us to understand their reasoning for their child to participate in the trial, and what has been positive and what can be improved in future similar studies.

During 2024 the trial protocol will be transferred to the European Union's Clinical Trials Information System (CTIS) (previously EudraCT). Until then the information on EudraCT is not updated. The EUCT number for the trial is 2023-504593-38-00. Please refer to ClinicalTrials.gov for the most recent information. The trial results will be uploaded on CTIS and ClinicalTrials.gov and published in scientific open access journals when the primary follow-up has been completed, and also when and the long-term follow-up has been completed. Lay summaries of the findings will be shared via the networks of the OI patient organisations OIFE and C4BB, and distributed to the families in the BOOSTB4 trial that have opted to receive such summaries. Details of the trial and progress will be shared by presentation at relevant national and international meetings.

For peer review only

Authors' contributions

CG, MW, EÅ, OS and ALD conceived the idea for the trial. All authors (RSL, EÅ, LSC, BC, ALD, CDV, AF, VF, GH, MH, MJ, CAL, PL, WHN, DO, MR, NES, RS, OS, MS, LWJ, EJTW, MW, CG) developed the trial protocol. CG, MW, EÅ, and LWJ wrote the initial trial protocol, which was subsequently revised by all authors. GH, MJ and NES wrote the initial texts on ethics and regulatory issues. EÅ developed the clinical practice of OI follow-up. MS developed the clinical practice and clinical safety parameters, and developed the study database. MH and LSC designed the research with stakeholders, MH conducted the research with stakeholders and contributed to the development of parent facing study materials. RS, EÅ and CG wrote the draft of the manuscript and all authors (RSL, EÅ, LSC, BC, ALD, CDV, AF, VF,

For peer review only - http://bmjopen.bmj.com/site/about/guidelines.xhtml

GH, MH, MJ, CAL, PL, WHN, DO, MR, NES, RS, OS, MS, LWJ, EJTW, MW, CG) revised, reviewed and approved the final manuscript.

Funding statement

This project has received funding from the European Union's Horizon 2020 research and innovation programme under grant agreement No 681045, Vetenskapsrådet (The Swedish Research Council,921-2014-7209 and 2020-01666), Region Stockholm in Sweden (20200365, FoUI-975495), the Center for Innovative Medicine (CIMED) in Sweden (FoUI-962010), and the Foundation for Children of the Freemasons in Stockholm. Parts of this work were supported by grants from the German Science Foundation (Deutsche Forschungsgemeinschaft, DFG) to the Research Unit FOR 2722; Refnr SE2373/1-2, Project 384170921. LSC is partially funded by the NIHR Great Ormond Street Hospital (GOSH) Biomedical Research Centre (BRC). All research at Great Ormond Street Hospital NHS Foundation Trust and UCL Great Ormond Street Institute of Child Health is made possible by the NIHR Great Ormond Street Hospital. ALD is partially funded by the NIHR University College London Hospitals BRC. The views expressed are those of the authors and not necessarily those of the NHS, the NIHR, or the UK Department of Health.

Competing interest statement

ALD is a consultant for Esperare Foundation for a clinical trial unrelated to this work. CG, LWJ and MW are co-founders and co-owners of BOOST Pharma ApS founded in 2020. OS is a scientific advisor for BOOST Pharma ApS. AF, BC, CAL, CDV, DO, EJTV, EÅ, GH, LSC, MH, MJ, MR, MS, NES, PL, RLS, RS, VF and WN declare no competing interests.

Data statement

Trial data is found at ClinicalTrials.gov ID NCT03706482. The Sponsor Karolinska Institutet and the Investigators will have access to the final trial dataset.

For peer review only - http://bmjopen.bmj.com/site/about/guidelines.xhtml

Acknowledgements

We thank all families taking part in the BOOSTB4 trial, and physicians collaborating in accomplishing the trial. We also thank OIFE and C4BB for their constructive and valuable support.

For peer review only

For peer review only - http://bmjopen.bmj.com/site/about/guidelines.xhtml

REFERENCES

1. Van Dijk FS, Sillence DO. Osteogenesis imperfecta: clinical diagnosis, nomenclature and severity assessment. *Am J Med Genet A* 2014;164A:1470-81.

2. Sillence DO, Senn A, Danks DM. Genetic heterogeneity in osteogenesis imperfecta. *J Med Genet* 1979;16:101-16.

3. Van Dijk FS, Pals G, Van Rijn RR, et al. Classification of Osteogenesis Imperfecta revisited. *Eur J Med Genet* 2010;53:1-5.

4. Thomas IH, DiMeglio LA. Advances in the Classification and Treatment of Osteogenesis Imperfecta. *Curr Osteoporos Rep* 2016;14:1-9.

5. Glorieux FH, Bishop NJ, Plotkin H, et al. Cyclic administration of pamidronate in children with severe osteogenesis imperfecta. *N Engl J Med* 1998;339:947-52.

6. Dwan K, Phillipi CA, Steiner RD, et al. Bisphosphonate therapy for osteogenesis imperfecta. *Cochrane Database Syst Rev* 2014;7:CD005088.

7. Hald JD, Evangelou E, Langdahl BL, et al. Bisphosphonates for the prevention of fractures in osteogenesis imperfecta: meta-analysis of placebo-controlled trials. *J Bone Miner Res* 2015;30:929-33.

8. Hill M, Lewis C, Riddington M, et al. Stakeholder views and attitudes towards prenatal and postnatal transplantation of fetal mesenchymal stem cells to treat Osteogenesis Imperfecta. *Eur J Hum Genet* 2019;27:1244-53.

9. Squillaro T, Peluso G, Galderisi U. Clinical Trials With Mesenchymal Stem Cells: An Update. *Cell Transplant* 2016;25:829-48.

10. Götherström C, Ringden O, Westgren M, et al. Immunomodulatory effects of human foetal liver-derived mesenchymal stem cells. *Bone Marrow Transplant* 2003;32:265-72.

11. Prockop DJ, Brenner M, Fibbe WE, et al. Defining the risks of mesenchymal stromal cell therapy. *Cytotherapy* 2010;12:576-8.

12. Pittenger MF, Mackay AM, Beck SC, et al. Multilineage potential of adult human mesenchymal stem cells. *Science* 1999;284:143-7.

13. Bernardo ME, Zaffaroni N, Novara F, et al. Human bone marrow derived mesenchymal stem cells do not undergo transformation after long-term in vitro culture and do not exhibit telomere maintenance mechanisms. *Cancer Res* 2007;67:9142-9.

14. Barkholt L, Flory E, Jekerle V, et al. Risk of tumorigenicity in mesenchymal stromal cell-based therapies –bridging scientific observations and regulatory viewpoints. *Cytotherapy* 2013;15:753-9.

15. von Bahr L, Batsis I, Moll G, et al. Analysis of tissues following mesenchymal stromal cell therapy in humans indicates limited long-term engraftment and no ectopic tissue formation. *Stem Cells* 2012;30:1575-8.

For peer review only

For peer review only - http://bmjopen.bmj.com/site/about/guidelines.xhtml

16. Rodriguez-Fuentes DE, Fernandez-Garza LE, Samia-Meza JA, et al. Mesenchymal Stem Cells Current Clinical Applications: A Systematic Review. *Arch Med Res* 2021;52:93-101.

17. Lalu MM, McIntyre L, Pugliese C, et al. Safety of cell therapy with mesenchymal stromal cells (SafeCell): a systematic review and meta-analysis of clinical trials. *PLoS One* 2012;7:e47559.

18. Thompson M, Mei SHJ, Wolfe D, et al. Cell therapy with intravascular administration of mesenchymal stromal cells continues to appear safe: An updated systematic review and meta-analysis. *EClinicalMedicine* 2020;19:100249.

19. Guillot PV, Gotherstrom C, Chan J, et al. Human first-trimester fetal MSC express pluripotency markers and grow faster and have longer telomeres than adult MSC. *Stem Cells* 2007;25:646-54.

20. Götherström C, West A, Liden J, et al. Difference in gene expression between human fetal liver and adult bone marrow mesenchymal stem cells. *Haematologica* 2005;90:1017-26.

21. Yu Y, Valderrama AV, Han Z, et al. Human fetal liver MSCs are more effective than adult bone marrow MSCs for their immunosuppressive, immunomodulatory, and Foxp3(+) T reg induction capacity. *Stem Cell Res Ther* 2021;12:138.

22. Götherström C, Ringden O, Tammik C, et al. Immunologic properties of human fetal mesenchymal stem cells. *Am J Obstet Gynecol* 2004;190:239-45.

23. Chan J, O'Donoghue K, Kennea N, et al. Myogenic potential of fetal mesenchymal stem cells. *Ann Acad Med Singapore* 2003;32:S11-3.

24. Kennea NL, Waddington SN, Chan J, et al. Differentiation of human fetal mesenchymal stem cells into cells with an oligodendrocyte phenotype. *Cell Cycle* 2009;8:1069-79.

25. Wei X, Wang CY, Liu QP, et al. In vitro hepatic differentiation of mesenchymal stem cells from human fetal bone marrow. *J Int Med Res* 2008;36:721-7.

26. Zhang ZY, Teoh SH, Chong MS, et al. Superior osteogenic capacity for bone tissue engineering of fetal compared with perinatal and adult mesenchymal stem cells. *Stem Cells* 2009;27:126-37.

27. Guillot PV, De Bari C, Dell'Accio F, et al. Comparative osteogenic transcription profiling of various fetal and adult mesenchymal stem cell sources. *Differentiation* 2008;76:946-57.

28. Zhang ZY, Teoh SH, Chong MS, et al. Neo-vascularization and bone formation mediated by fetal mesenchymal stem cell tissue-engineered bone grafts in critical-size femoral defects. *Biomaterials* 2010

29. Guillot PV, Abass O, Bassett JH, et al. Intrauterine transplantation of human fetal mesenchymal stem cells from first-trimester blood repairs bone and reduces fractures in osteogenesis imperfecta mice. *Blood* 2008;111:1717-25.

30. Panaroni C, Gioia R, Lupi A, et al. In utero transplantation of adult bone marrow decreases perinatal lethality and rescues the bone phenotype in the knockin murine model for classical, dominant osteogenesis imperfecta. *Blood* 2009;114:459-68.

For peer review only - http://bmjopen.bmj.com/site/about/guidelines.xhtml

31. Vanleene M, Saldanha Z, Cloyd KL, et al. Transplantation of human fetal blood stem cells in the osteogenesis imperfecta mouse leads to improvement in multiscale tissue properties. *Blood* 2011;117:1053-60.

32. Horwitz EM, Prockop DJ, Fitzpatrick LA, et al. Transplantability and therapeutic effects of bone marrow-derived mesenchymal cells in children with osteogenesis imperfecta. *Nat Med* 1999;5:309-13.

33. Horwitz EM, Prockop DJ, Gordon PL, et al. Clinical responses to bone marrow transplantation in children with severe osteogenesis imperfecta. *Blood* 2001;97:1227-31.

34. Horwitz EM, Gordon PL, Koo WK, et al. Isolated allogeneic bone marrow-derived mesenchymal cells engraft and stimulate growth in children with osteogenesis imperfecta: Implications for cell therapy of bone. *Proc Natl Acad Sci U S A* 2002;99:8932-7.

35. Infante A, Gener B, Vazquez M, et al. Reiterative infusions of MSCs improve pediatric osteogenesis imperfecta eliciting a pro-osteogenic paracrine response: TERCELOI clinical trial. *Clin Transl Med* 2021;11:e265.

36. Infante A, Cabodevilla L, Gener B, et al. Circulating TGF-beta Pathway in Osteogenesis Imperfecta Pediatric Patients Subjected to MSCs-Based Cell Therapy. *Front Cell Dev Biol* 2022;10:830928.

37. Le Blanc K, Gotherstrom C, Ringden O, et al. Fetal mesenchymal stem-cell engraftment in bone after in utero transplantation in a patient with severe osteogenesis imperfecta. *Transplantation* 2005;79:1607-14.

38. Götherström C, Westgren M, Shaw SW, et al. Pre- and postnatal transplantation of fetal mesenchymal stem cells in osteogenesis imperfecta: a two-center experience. *Stem Cells Transl Med* 2014;3:255-64.

39. Pollitt R, McMahon R, Nunn J, et al. Mutation analysis of COL1A1 and COL1A2 in patients diagnosed with osteogenesis imperfecta type I-IV. *Hum Mutat* 2006;27:716.

40. Kidszun A, Linebarger J, Walter JK, et al. What If the Prenatal Diagnosis of a Lethal Anomaly Turns Out to Be Wrong? *Pediatrics* 2016;137

41. Spencer RN, Hecher K, Norman G, et al. Development of standard definitions and grading for Maternal and Fetal Adverse Event Terminology. *Prenat Diagn* 2022;42:15-26.

42. Rapoport M, Bober MB, Raggio C, et al. The patient clinical journey and socioeconomic impact of osteogenesis imperfecta: a systematic scoping review. *Orphanet J Rare Dis* 2023;18:34.

43. Kolovos S, Javaid MK, Pinedo-Villanueva R. Hospital admissions of patients with osteogenesis imperfecta in the English NHS. *Osteoporos Int* 2021;32:1207-16.

44. Darba J, Marsa A. Hospital incidence, management and direct cost of osteogenesis imperfecta in Spain: a retrospective database analysis. *J Med Econ* 2020;23:1435-40.

45. Johansson M, Hermeren G, Sahlin NE. Paternal consent in prenatal research: ethical aspects. *Med Health Care Philos* 2020;23:325-31.

For peer review only - http://bmjopen.bmj.com/site/about/guidelines.xhtml

Table 1. Inclusion and Exclusion criteria for subjects in the BOOSTB4 trial.

For inclusion, all criteria should apply. For exclusion, one is enough to exclude.

Prenatal group: After birth and before the 2nd dose, subjects in the prenatal group will be assessed for the inclusion and exclusion criteria for the postnatal group.

INCLUSION CRITERIA		
**Postnatal group (n=15)[1, 2]**	**Prenatal group (n=3)[1, 3, 4]**	**Control group (n=18–90)[1]**
1. Parent's/legal guardian's signed informed-consent form,<br>2. Clinical diagnosis of OI type 3 or severe 4 AND<br>3. Molecular diagnosis of OI (Glycine substitution in the collagen triple-helix encoding region of either the *COL1A1* or *COL1A2* gene),<br>4. Age less than 18 months[5] (calculated from gestational week 40+0, i.e. the corrected age),<br>5. Parent/legal guardian over 18 years of age.	1. Woman has signed the patient consent form,<br>2. Only women where termination of the pregnancy is no longer possible or where the women are committed to continue the pregnancy may be included in the trial,<br>3. Suspicion of OI type 3 or severe 4 in the fetus on ultrasound findings AND<br>4. Molecular diagnosis of OI in the fetus (Glycine substitution in the collagen triple-helix encoding region of either the *COL1A1* or *COL1A2* gene),<br>5. Gestation age between 16+0 and 35+6 weeks+days,<br>6. Pregnant women over 18 years of age.	*Matched historical controls:*<br>1. Parent's/legal guardian's signed informed-consent form,<br>2. Clinical and molecular diagnosis of OI (Glycine substitution in the collagen triple-helix encoding region of either the *COL1A1* or *COL1A2* gene),<br>3. Data on fractures and growth is available,<br>4. Parent/legal guardian over 18 years of age.<br>*Prospective untreated controls:*<br>- Postnatal participation: The inclusion criteria for the postnatal group apply.<br>- Prenatal participation: The inclusion criteria for the prenatal group apply, except inclusion criteria 2.

[1]All subjects (the postnatal, prenatal and control groups) will receive bisphosphonate treatment.

[2]At least one dose of bisphosphonate must be administered before the first dose of stem cells.

[3]The first dose of bisphosphonates will be administered after birth.

[4]In the original trial protocol the prenatal group was n=15. This amendment has been approved by the Competent Authorities.

[5]In the original trial protocol it was "Age less than 12 months". This amendment has been approved by the Competent Authorities.

For peer review only - http://bmjopen.bmj.com/site/about/guidelines.xhtml

EXCLUSION CRITERIA		
**Postnatal group (n=15)**	**Prenatal group (n=3)[4]**	**Control group (n=18–90)**
1. Existence of other known disorder that might interfere with the treatment, such as, but not limited to organ disfunction (for example liver or renal failure or bronchopulmonary dysplasia), congenital heart defect, hypoxic encephalopathy I-III, severe neurological problems, immune deficiencies, muscle diseases, severe malformations or syndromes diagnosed by clinical examination,<br>2. Any contraindication for invasive procedures such as a moderate/severe bleeding tendency,<br>3. Known risk factors for clotting, such as, but not limited to previous blood clot, family history of clots, clotting disorder (inherited or acquired), heart failure, inflammatory disorders (for example lupus, rheumatoid arthritis, inflammatory bowel disease),<br>4. Positive Donor Specific Antibody-test,<br>5. Known allergy/hypersensitivity to Fungizone and/or Gensumycin,<br>6. Abnormal karyotype or other confirmed genetic syndromes,<br>7. Oncologic disease (previous or current malignancy),<br>8. Inability to comply with the trial protocol and follow-up schedule,<br>9. Inability to understand the information and to provide informed consent.	1. Multiple pregnancy,<br>2. Co-existence of other disorder that might interfere with the treatment, as judged by the Investigator or the patient's obstetrician,<br>3. Abnormal fetal karyotype or other confirmed genetic syndrome,<br>4. Any contraindication for invasive procedures such as a bleeding tendency or contagious infections, such as, but not limited to HIV, Syphilis, Hepatitis B, Hepatitis C or other known infectious diseases that can harm the fetus,<br>5. Known risk factors for clotting, such as, but not limited to previous blood clot, family history of clots, clotting disorder (inherited or acquired), heart failure, inflammatory disorders (for example lupus, rheumatoid arthritis, inflammatory bowel disease),<br>6. Positive Donor Specific Antibody-test,<br>7. Known allergy/hypersensitivity to Fungizone and/or Gensumycin,<br>8. Oncologic disease in woman or fetus (previous or current malignancy),<br>9. Unwilling to or cannot undergo delivery by Caesarean section,<br>10. Inability to comply with the trial protocol and follow-up schedule,<br>11. Inability to understand the information and to provide informed consent.	*Matched historical controls (15/subject):*<br>1. Existence of other disorder that might interfere with the trial. No lung hypoplasia (type 2 OI),<br>2. Abnormal karyotype.<br>*Prospective untreated controls (n=up to 30):*<br>- Postnatal participation: The exclusion criteria, except exclusion criterium 2, 3, 4 and 5 (Contraindication for invasive procedure, Known risk factor for clotting, Positive Donor Specific Antibody-test and Known allergy/hypersensitivity to Fungizone and/or Gensumycin) for the postnatal group apply.<br>- Prenatal participation: The exclusion criteria, except exclusion criterium 1, 4, 5, 6 and 7 (Multiple pregnancy, Contraindication for invasive procedure, Known risk factor for clotting, Positive Donor Specific Antibody-test and Known allergy/hypersensitivity to Fungizone and/or Gensumycin) for the prenatal group apply.

For peer review only

Table 2. Matching of the controls.

One to five historical controls per subject and up to 30 prospective controls in total will be included and matched to each subject.

**MATCHING OF THE CONTROLS**	
The controls historical and untreated prospective) will be individually matched to each trial subject:	
1. Type of OI[1]:	- Type 3 with type 3.<br>- Severe type 4 with Severe type 4.
2. Age at onset of bisphosphonate therapy:	- Start of bisphosphonate treatment in the first 2 months of life.<br>- Start of bisphosphonate treatment from 2–12 months of life.
If possible (if more than 5 controls have been identified), matching will also be made on:	
3. Sex	
4. Identical OI pathogenic sequence variant	
5. Time for OI diagnosis	- A control matched with a subject in the postnatal group was diagnosed[2] postnatally.<br>- A control matched with a subject in the prenatal group was diagnosed[2] prenatally.

[1]No mixed OI types (for e.g. OI type 3/4, is allowed).

[2]Clinical diagnosis, molecular diagnosis not required at this time point.

For peer review only - http://bmjopen.bmj.com/site/about/guidelines.xhtml

FIGURE LEGENDS

Figure 1. Overview of the trial schedule.

The BOOSTB4 trial consists of 2 parts; Part 1 that includes information, consent, screening and inclusion. This is followed by 4 doses of BOOST cells at 4±1 month intervals with immediate in-patient follow-up over 48 hours after dose 1–2, and over 24 hours after dose 3–4 (48 hours for dose 1–3 in the prenatal group). Part 1 of the trial also includes the primary follow-up at 6 and 12 months after the 4th and last dose. Part 2 of the trial consists of yearly long-term follow-up over 8 years until 10 years after the first dose.

For peer review only

For peer review only - http://bmjopen.bmj.com/site/about/guidelines.xhtml

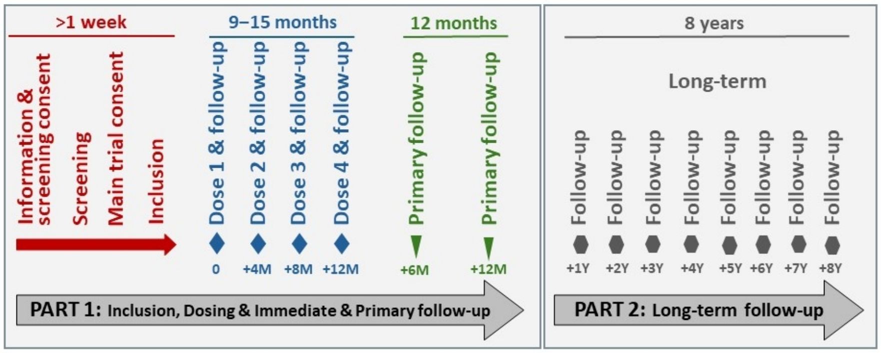

Figure 1. Overview of the trial schedule.

The BOOSTB4 trial consist of 2 parts; Part 1 that includes information, consent, screening and inclusion. This is followed by 4 doses of BOOST cells at 4±1 month intervals with immediate in-patient follow-up over 48 hours after dose 1–2, and over 24 hours after dose 3–4 (48 hours for dose 1–3 in the prenatal group). Part 1 of the trial also includes the primary follow-up at 6 and 12 months after the 4th and last dose. Part 2 of the trial consist of yearly long-term follow-up over 8 years until 10 years after the first dose.

275x190mm (96 x 96 DPI)

Clinical trial Synopsis

TRIAL REGISTRATION DATA SET	
**Data category**	**Information**
Registry trial identifying number	EudraCT no: 2015-003699-60, ClinicalTrials.gov ID: NCT03706482
Secondary identifying numbers	KIBB01
Sources of monetary support	Academic: European Union's Horizon 2020 research and innovation programme under grant agreement No 681045, the Swedish Research Council, Region Stockholm in Sweden
Sponsor	Karolinska Institutet
Contact for queries	boostb4@clintec.ki.se
Title	An exploratory, open label, multiple dose, multicentre phase I/II trial evaluating safety and efficacy of postnatal or prenatal and postnatal intravenous administration of allogeneic expanded fetal mesenchymal stem cells for the treatment of severe Osteogenesis Imperfecta compared with a combination of historical and untreated prospective controls.
Trial code	BOOSTB4
Countries of recruitment	Sweden, Netherlands, United Kingdom
Health condition studied	Osteogenesis Imperfecta type 3 and severe type 4
Intervention	Intravenous administration of BOOST cells
Investigational Medicinal Product	BOOST cells (expanded human 1st trimester fetal liver-derived mesenchymal stem cells)
Study type	Interventional
	Open label
	Primary purpose: Treatment of chronic condition
	Phase I/II
Trial groups	Postnatal group (4 postnatal doses), n=15
	Prenatal group (1 prenatal dose & 3 postnatal doses), n=3
	Prospective control group, n=up to 30
	Historical control group, n=1–5 per subject
Date of first enrolment	March 2020
Target sample size	Initially n=15 in the postnatal group and n=15 in the prenatal group, later amended to n=15 in the postnatal group and n=3 in the prenatal group
Recruitment status	Completed
Primary outcomes	Safety and tolerability
Key secondary outcomes	Efficacy: fracture incidence, growth, bone mineral density

For peer review only - http://bmjopen.bmj.com/site/about/guidelines.xhtml

Protocol Summary

PROTOCOL IDENTITY

Trial Title: Boost Brittle Bones Before Birth (BOOSTB4)

An exploratory, open label, multiple dose, multicentre phase I/II trial evaluating safety and efficacy of postnatal or prenatal and postnatal intravenous administration of allogeneic expanded fetal mesenchymal stem cells for the treatment of severe Osteogenesis Imperfecta compared with a combination of historical and untreated prospective controls.

EudraCT number	Protocol Number	Trial Code	Phase of Development
2015-003699-60	KIBB01	BOOSTB4	I/II

Sponsor: Karolinska Institutet, Stockholm, Sweden

Sponsor's representative: Associate Professor Cecilia Götherström, Karolinska Institutet, Department of Clinical Science, Technology and Intervention, Division of Obstetrics and Gynaecology, Stockholm, Sweden

Principal Coordinating Investigator: Dr Eva Åström, Pediatric Neurology and Musculoskeletal disorders and Home care, Astrid Lindgren Children's Hospital at Karolinska University Hospital, Stockholm, Sweden

Principal Investigator: Dr Eva Åström, Pediatric Neurology and Musculoskeletal disorders and Home care, Astrid Lindgren Children's Hospital at Karolinska University Hospital, Stockholm, Sweden

Trial centres: Karolinska University Hospital, Sweden[1]; University College London Hospitals NHS Foundation Trust[2] and Great Ormond Street Hospital for Children NHS Foundation Trust[2], the United Kingdom; University Hospital Cologne[2], Germany and Leiden University Medical Centre[3] and University Medical Centre Utrecht[3], the Netherlands.

[1]Performing the active part of the trial, i.e. dose administration and immediate, primary and long-time follow-up, and inclusion of historical and prospective controls.

[2]Performing inclusion of historical controls.

[3]Performing inclusion of historical and prospective controls and primary and long-time follow-up of two Dutch subjects.

INVESTIGATIONAL MEDICINAL PRODUCT (IMP)

Test Product: BOOST cells (allogeneic expanded human 1st trimester liver-derived mesenchymal stem cells [MSC])

Pharmaceutical Form: Cell suspension

Dose: $3x10^6$ viable BOOST cells/kg body weight

Route of Administration: Intravenous administration postnatally or prenatally

Manufacturer: Karolinska Cell Therapy Centre, Vecura at Karolinska University Hospital in Sweden

For peer review only - http://bmjopen.bmj.com/site/about/guidelines.xhtml

TRIAL DESIGN

Hypothesis: Early administration of MSC will ameliorate severe types of OI.

Trial design:
This will be a multi-centre, open label, multiple-dose, phase I/II trial of allogeneic expanded human first trimester fetal MSC administration for the treatment of Osteogenesis Imperfecta (OI) type 3 or severe type 4 compared with historical and untreated prospective controls.

The trial is divided into 2 parts where Part 1 includes administration of 4 postnatal doses per subject and Part 2 includes administration of one prenatal and 3 postnatal doses per subject:

Part 1: Postnatal administration (n=15 in total)

- Administration of four postnatal doses of MSC to 15 subjects
- A safety assessment is performed by the Data Safety Monitoring Board (DSMB) of the first MSC administration to the first 5 subjects[1] in any participating country treated postnatally.

[1]The 5 subjects included and administered with 1 MSC dose postnatally in Part 1 of the trial will receive the additional 3 MSC doses as planned, i.e. at +4, +8 and +12 months after the first MSC dose. Available data from such administration will be included in the data set sent to the DSMB for the safety assessment. There will be no planned pause in the postnatal administration during the safety assessment by the DSMB for opening of Part 2 of the trial. The Stopping rules on subject level apply to all steps (see Section 10.9).

Part 2: Prenatal administration (n=3 in total)

- One prenatal MSC dose to 3 subjects[2].

[2]The 3 subjects included and administered with 1 MSC dose prenatally in Part 2 of the trial will receive the additional 3 postnatal MSC doses as planned, i.e. at +4, +8 and +12 months after the prenatal dose. The Stopping rules on subject level apply to all steps (see Section 10.9).

Duration:
The trial is divided into two periods where Period 1 includes the administration of MSC and immediate and primary follow-up, and Period 2 that includes the long-time follow-up (see the Trial schedule below):

Period 1: Four doses of MSC every 4 months with immediate follow-up after each MSC dose, and primary follow-up at 6 and 12 months after the fourth and last MSC dose

Period 2: Yearly long-time follow-up until 10 years after the first MSC dose

Subjects in the postnatal group will be included, receive the first dose and evaluated as soon as possible after birth up to 18 months of corrected age. They will receive 3 additional doses at +4, +8 and +12 months (±1 month) after the 1st dose.

Subjects in the prenatal group will be included in the trial and receive the 1st dose prenatally (between 16+0 – 35+6 weeks+days of gestation) and receive 3 additional postnatal doses at +4 (or as soon as possible after birth), +8 and +12 months (±1 month) after the 1st dose.

Subjects can be enrolled during the initial 3 years of the trial. The expected duration of treatment is 12 months, the minimum treatment time is 9 months and the maximum treatment time is 15 months.

For peer review only - http://bmjopen.bmj.com/site/about/guidelines.xhtml

Trial Schedules in Period 1 (GW=gestation weeks in weeks+days. All postnatal time points are ± 1 month)**:**

Postnatal group

Diagnosis	Dose 1 & immediate follow-up	Dose 2 & immediate follow-up	Dose 3 & immediate follow-up	Dose 4 & immediate follow-up	Primary follow-up	Primary follow-up
Clinical & molecular diagnosis of type 3 / severe type 4 OI	As soon as possible after birth up to 18 months of age	+4 months after dose 1	+4 months after dose 2	+4 months after dose 3	+6 months after dose 4	+12 month after dose 4

Prenatal group

Diagnosis	Dose 1 & immediate follow-up	Post prenatal dose follow-up	Birth	Dose 2 & immediate follow-up	Dose 3 & immediate follow-up	Dose 4 & immediate follow-up	Primary follow-up	Primary follow-up
Clinical & molecular diagnosis of type 3 / severe type 4 OI	Gestation weeks 16+0 – 35+6	Every 2 weeks up to birth		+4 months after dose 1	+4 months after dose 2	+4 months after dose 3	+6 months after dose 4	+12 months after dose 4

For peer review only

TRIAL OBJECTIVES

Primary objectives:

To assess safety and tolerability in the child, fetus and woman after postnatal or prenatal and postnatal intravenous administration of 4 doses of BOOST cells every 4 months in subjects with OI type 3 or severe type 4.

Secondary objectives:

To assess the effect of intravenous administration of 4 doses of BOOST cells every 4 months in subjects with OI type 3 or severe type 4 on:

1. Number of fractures from baseline to primary and long-time follow-up
2. Time (days) to first fracture after last dose
3. Number of fractures at birth (prenatal treatment group only, and postnatal treatment group when available)
4. Bone mineral density (BMD)
5. Growth
6. Clinical status of OI
7. Biochemical bone turnover

Exploratory objectives

1. To study the impact of 4 doses of fetal MSC on Quality of Life (QoL)
2. To study the extent of donor cell engraftment in tissue samples
3. To study paracrine effects of 4 doses of fetal MSC
4. To study the effect of 4 doses of fetal MSC on endogenous immune cells
5. To study non-invasive prenatal diagnosis of OI (will not be used in the diagnostic procedure in the trial)

TRIAL ENDPOINTS

Primary Endpoints:

The primary endpoint is safety and tolerability measured as seriousness, severity and frequency of MSC administration related adverse events (AEs), with specific focus on the following:

For peer review only - http://bmjopen.bmj.com/site/about/guidelines.xhtml

1. Vital signs in conjunction with the MSC administration
2. Transfusion reactions (administration toxicity, allergy, embolism)
3. Immune reaction with or without symptoms of inflammation, potentially resulting in rejection of the cells or development of donor-specific antibodies:
   - Allergy or Hypersensitivity responses to antibiotics or antimycotics
   - Development of Fetal Bovine Serum-specific antibodies
   - Hypersensitivity responses to Human Serum Albumin
   - Hypersensitivity to impurities in the IMP
4. Prenatal complications (miscarriage/intrauterine fetal death, premature birth, infection *in utero* or persistent [>1 min] fetal bradycardia) in the prenatal group
5. Adverse effects of feto-maternal transmission of donor cells in the prenatal group
6. Tumourigenicity
7. Mortality/morbidity

Key Secondary Endpoint:

1. Number of fractures from baseline to primary and long-time follow-up

Secondary Endpoints:

1. Time (days) to first fracture after last dose
2. Number of fractures at birth (prenatal group, and postnatal group when available)
3. Change in BMD (g/cm^2)
4. Growth (cm and kg)
5. Change in clinical status of OI based on parameters defined under efficacy assessments described below
6. Change in biochemical bone turnover

Exploratory endpoints:

1. The QoL will be assessed through the Infant Toddler Quality of Life Questionnaire™ (ITQOL)
2. Tissue samples (bone, bone marrow, muscle and skin, placenta, amniotic fluid, umbilical cord, umbilical cord blood, peripheral blood) will be collected in conjunction with clinically indicated surgery and at birth (prenatal group only) and analysed for donor cell engraftment
3. Paracrine effects will be analysed from plasma isolated from peripheral blood
4. Effect on immune cells will be analysed from peripheral blood
5. Non-invasive prenatal diagnosis will be studied during the trial

POPULATION OF TRIAL SUBJECTS

Description of Trial Subjects:

1. Infants diagnosed with OI type 3 or severe type 4 on clinical grounds with a confirmatory sequence variant (mutation) in one or other of the type I collagen genes (*COL1A1* and *COL1A2*) (n=15)
2. Pregnant women whose fetus has been diagnosed with OI type 3 or severe type 4 prenatally on ultrasound parameters with a confirmatory sequence variant (mutation) in one or other of the type I collagen genes (*COL1A1* and *COL1A2*) (n=3)
3. Controls diagnosed with OI type 3 or severe type 4 on clinical grounds with a confirmatory sequence variant (mutation) in one or other of the type I collagen genes (*COL1A1* and *COL1A2*):
   a) Matched historical controls (n=30-150) will be identified in national registries
   b) Prospective untreated controls (n=up to 30). Subjects eligible for the trial but not willing/able to participate.

For peer review only

For peer review only - http://bmjopen.bmj.com/site/about/guidelines.xhtml

Only patients diagnosed with qualitative (Glycine) substitution in the collagen triple-helix encoding region of either the *COL1A1* or *COL1A2* gene will be included in the trial. The diagnosis will be made both clinically and with molecular analysis by a multidisciplinary team.

No formal power calculations have been performed. OI is a rare disease and a limited number of subjects can be included each year. Based on the incidence, it is reasonable to include 30 patients in this first trial on the grounds of feasibility, suitable time period and for the application of descriptive statistical analyses.

INCLUSION CRITERIA

For inclusion, all criteria should apply.

Postnatal group

1. Parent's/legal guardian's signed informed-consent form
2. Clinical diagnosis of OI type 3 or 4 AND
3. Molecular diagnosis of OI (Glycine substitution in the collagen triple-helix encoding region of either the *COL1A1* or *COL1A2* gene)
4. Age less than 18 months (calculated from gestational week 40+0, i.e. the corrected age)
5. Parent/legal guardian over 18 years of age

Prenatal group:

1. Woman has signed the patient consent form
2. Only women where termination of the pregnancy is no longer possible or where the women are committed to continue the pregnancy may be included in the trial
3. Suspicion of OI type 3 or 4 in the fetus on ultrasound findings AND
4. Molecular diagnosis of OI in the fetus (Glycine substitution in the collagen triple-helix encoding region of either the *COL1A1* or *COL1A2* gene)
5. Gestation age between 16+0 and 35+6 weeks+days
6. Pregnant women over 18 years of age

After birth and before the 2nd dose, subjects in the prenatal group will be assessed for the inclusion criteria for the postnatal group.

Control group:

a) Matched historical controls

1. Parent's/legal guardian's signed informed-consent form
2. Clinical and molecular diagnosis of OI (Glycine substitution in the collagen triple-helix encoding region of either the *COL1A1* or *COL1A2* gene)
3. Data on fractures and growth is available
4. Parent/legal guardian over 18 years of age

b) Prospective untreated controls

Postnatal participation: The inclusion criteria for the postnatal group apply

Prenatal participation: The inclusion criteria for the prenatal group apply, except inclusion criteria 2.

For peer review only - http://bmjopen.bmj.com/site/about/guidelines.xhtml

EXCLUSION CRITERIA

For exclusion, one is enough to exclude.

Postnatal group:

1. Existence of other known disorder that might interfere with the treatment, such as, but not limited to organ disfunction (for example liver or renal failure or bronchopulmonary dysplasia), congenital heart defect, hypoxic encephalopathy I-III, severe neurological problems, immune deficiencies, muscle diseases, severe malformations or syndromes diagnosed by clinical examination
2. Any contraindication for invasive procedures such as a moderate/severe bleeding tendency
3. Known risk factors for clotting, such as, but not limited to previous blood clot, family history of clots, clotting disorder (inherited or acquired), heart failure, inflammatory disorders (for example lupus, rheumatoid arthritis, inflammatory bowel disease)
4. Positive Donor Specific Antibody-test
5. Known allergy/hypersensitivity to Fungizone and/or Gensumycin
6. Abnormal karyotype or other confirmed genetic syndromes
7. Oncologic disease (previous or current malignancy)
8. Inability to comply with the trial protocol and follow-up schedule
9. Inability to understand the information and to provide informed consent

Prenatal group:

1. Multiple pregnancy
2. Co-existence of other disorder that might interfere with the treatment, as judged by the Investigator or the patient's obstetrician
3. Abnormal fetal karyotype or other confirmed genetic syndrome
4. Any contraindication for invasive procedures such as a bleeding tendency or contagious infections, such as, but not limited to HIV, Syphilis, Hepatitis B, Hepatitis C or other known infectious diseases that can harm the fetus
5. Known risk factors for clotting, such as, but not limited to previous blood clot, family history of clots, clotting disorder (inherited or acquired), heart failure, inflammatory disorders (for example lupus, rheumatoid arthritis, inflammatory bowel disease)
6. Positive Donor Specific Antibody-test
7. Known allergy/hypersensitivity to Fungizone and/or Gensumycin
8. Oncologic disease in woman or fetus (previous or current malignancy)
9. Unwilling to or cannot undergo delivery by Caesarean section
10. Inability to comply with the trial protocol and follow-up schedule
11. Inability to understand the information and to provide informed consent

After birth and before the 2nd dose, subjects in the prenatal group will be assessed for the exclusion criteria for the postnatal group.

Control group:

a) Matched historical controls

1. Existence of other disorder that might interfere with the trial. No lung hypoplasia (type 2 OI).
2. Abnormal karyotype

b) Prospective untreated controls

Postnatal participation: The exclusion criteria, except exclusion criterium 2, 3, 4, and 5 (Contraindication for invasive procedure, Known risk factor for clotting, Positive Donor Specific

For peer review only - http://bmjopen.bmj.com/site/about/guidelines.xhtml

Antibody-test and Known allergy/hypersensitivity to Fungizone and/or Gensumycin), for the postnatal group apply.

Prenatal participation: The exclusion criteria, except exclusion criterium 1, 4, 5, 6 and 7 (Multiple pregnancy, Contraindication for invasive procedure, Known risk factor for clotting, Positive Donor Specific Antibody-test and Known allergy/hypersensitivity to Fungizone and/or Gensumycin), for the prenatal group apply.

MATCHING OF THE CONTROLS

Parents/authorized representatives of children diagnosed with OI type 3 or severe OI type 4 will be informed about the trial and asked to give consent to the child's participation in the trial as a matched historical control. Subjects eligible for the trial but not willing/able to participate in the trial will be asked for consent to be included as prospective controls.

The controls (historical and untreated prospective) will be individually matched to each trial subject:

1. Type of OI*:
   Type 3 with Type 3
   Severe Type 4 with Severe Type 4
   *No mixed OI types (for e.g. OI type 3/4, is allowed)
2. Age at onset of BP therapy:
   Start of BP treatment in the first 2 months of life.
   Start of BP treatment from 2–12 months of life.

If possible (if more than 5 controls have been identified), matching will also be made on:

3. Sex
4. Identical OI mutation
5. Time for diagnosis:
   A control matched with a subject in the postnatal group was diagnosed** postnatally
   A control matched with a subject in the prenatal group was diagnosed** prenatally
   **Clinical diagnosis, molecular diagnosis not required at this time point.

For peer review only

For peer review only - http://bmjopen.bmj.com/site/about/guidelines.xhtml

Karolinska Institutet BOOSTB4 Boost Brittle Bones Before Birth KAROLINSKA UNIVERSITETSSJUKHUSET

BOOSTB4: A clinical research trial of a new intervention for brittle bone disease

SCREENING CONSENT FORM FOR THE POSTNATAL TRIAL GROUP

TO BE COMPLETED BEFORE SCREENING INVESTIGATIONS TAKE PLACE

Consent part 1

I confirm I have read and understood the Participant Information for the above trial. I confirm that I have received sufficient explanation of the BOOSTB4 Clinical Trial and have had the opportunity to consider the information and ask questions which have been answered satisfactorily. I have been given the Participant Information to keep.

I understand that my child's participation is voluntary and that I am free to withdraw them at any time without giving any reason, without their medical care or legal rights being affected.

I give consent for members of the BOOSTB4 Clinical Trial Team to review my child's medical records and use the data contained within, including access to all investigation results relevant for the purposes of this trial.

I give consent for members of the BOOSTB4 Clinical Trial Team to perform a screening investigation and a screening examination of my child as detailed in the Participant Information.

I agree that biological samples collected from my child are a gift, and can be used in the research described in the Participant Information.

I agree that all samples can be stored coded in the Stockholm Medical Biobank (reg. no. 914).

I agree that all coded samples can be transferred to other hospitals or laboratories in other countries for specialist tests.

For peer review only - http://bmjopen.bmj.com/site/about/guidelines.xhtml

Consent part 1

Patient Trial Identification Number: ___________________________ (filled in by the trial)

Date of Birth (dd/mm/yyyy) / social security no (personnummer): ____________________

Name of parent	Date (dd/mm/yyyy)	Signature
Name of parent (optional)	Date (dd/mm/yyyy)	Signature
Name of person taking consent	Date (dd/mm/yyyy)	Signature
Name of interpreter (if applicable)	Date (dd/mm/yyyy)	Signature

For peer review only

For peer review only - http://bmjopen.bmj.com/site/about/guidelines.xhtml

Karolinska Institutet | BOOSTB4 Boost Brittle Bones Before Birth | KAROLINSKA UNIVERSITETSSJUKHUSET

Consent part 2

Voluntary, mark the Yes/No boxes according to your consent.

	YES	NO
I agree that all biological samples can be stored coded at the Stockholm Medical Biobank (reg. no. 914) for future ethically approved research.	☐	☐

Name of parent	Date (dd/mm/yyyy)	Signature
Name of parent (optional)	Date (dd/mm/yyyy)	Signature
Name of person taking consent	Date (dd/mm/yyyy)	Signature
Name of interpreter (if applicable)	Date (dd/mm/yyyy)	Signature

For peer review only

For peer review only - http://bmjopen.bmj.com/site/about/guidelines.xhtml

BOOSTB4: A clinical research trial of a new intervention for brittle bone disease

MAIN TRIAL CONSENT FORM FOR THE POSTNATAL TRIAL GROUP

TO BE COMPLETED AFTER SCREENING INVESTIGATIONS HAVE BEEN PERFORMED AND ELIGIBILITY CONFIRMED

Consent part 1

I confirm I have read and understood the Participant Information for the above trial. I confirm that I have received sufficient explanation of the BOOSTB4 Clinical Trial and have had the opportunity to consider the information and ask questions which have been answered satisfactorily. I have been given the Participant Information to keep.

I understand that my child's participation is voluntary and that I am free to withdraw them at any time without giving any reason, without their medical care or legal rights being affected.

I understand that relevant sections of my child's medical notes and data collected during the trial may be looked at by responsible individuals from national and international competent authorities, the BOOSTB4 team medical doctors and clinical trial monitors that inspect trial safety, where it is relevant to my child's taking part in this research. I give permission for these individuals to have access to my child's records. This includes medical notes and data collected at the clinical research site, medical notes and data collected within the health care (i.e. the general/family doctor and other community services). I consent to that data from my child are treated as described in the Participant Information.

I agree to my child's doctor and local hospital being informed of my child's participation in the trial. I agree that they can be contacted for medical information or obtaining up-to-date contact details.

I agree to my child taking part in the BOOSTB4 Clinical Trial.

I agree that my child can not participate in another clinical trial before the 12-month follow-up after the last stem cell dose, and that any participation in other trials must be discussed with the trial doctor.

I agree that biological samples collected from my child are a gift as described in the Participant Information.

I agree that all samples can be stored coded in the Stockholm Medical Biobank (reg. no. 914).

For peer review only - http://bmjopen.bmj.com/site/about/guidelines.xhtml

I agree that all coded samples can be transferred to other hospitals or laboratories in other countries for specialist tests.

I understand that this study using the samples may include genetic research aimed at understanding the genes related to OI and how the environment affects them (epigenetics) but that the results of these investigations are unlikely to have any implications for my child personally.

I agree that all discoveries (intellectual property) are a gift to the researchers and understand that my child will not benefit financially if the research leads to a new treatment or medical test.

I agree that I can be contacted by telephone to collect follow-up information about my child.

Consent part 1

Patient Trial Identification Number: ______________________________ (filled in by the trial)

Date of Birth (dd/mm/yyyy) / social security no: ______________________________________

Name of parent	Date (dd/mm/yyyy)	Signature
Name of parent (optional)	Date (dd/mm/yyyy)	Signature
Name of person taking consent	Date (dd/mm/yyyy)	Signature
Name of interpreter (if applicable)	Date (dd/mm/yyyy)	Signature

2 copies required: Original for Investigator Site File and copy for participant.

For peer review only - http://bmjopen.bmj.com/site/about/guidelines.xhtml

Consent part 2

Voluntary, mark the Yes/No boxes according to your consent.

	YES	NO
I agree that all biological samples can be stored coded in the Stockholm Medical Biobank (reg. no. 914) for future ethically approved research.	☐	☐
I agree to give consent for photographs and video footage to be taken of trial procedures; the anonymised images may be used for educational purposes or be published.	☐	☐
I agree to give contact details for a relative / friend that the BOOSTB4 trial team can contact if they are unable to get in touch with me in the future.	☐	☐
I agree to the BOOSTB4 trial team contacting me in the future about trial related activities and events.	☐	☐
I would like to receive a summary of the trial results (it will take up to five years).	☐	☐

Name of parent	Date (dd/mm/yyyy)	Signature
Name of parent (optional)	Date (dd/mm/yyyy)	Signature
Name of person taking consent	Date (dd/mm/yyyy)	Signature
Name of interpreter (if applicable)	Date (dd/mm/yyyy)	Signature

For peer review only - http://bmjopen.bmj.com/site/about/guidelines.xhtml

Karolinska Institutet   BOOSTB4 Boost Brittle Bones Before Birth   KAROLINSKA UNIVERSITETSSJUKHUSET

BOOSTB4: A clinical research trial of a new intervention for brittle bone disease

SCREENING CONSENT FORM FOR THE PRENATAL TRIAL GROUP

TO BE COMPLETED BEFORE SCREENING INVESTIGATIONS TAKE PLACE

Consent part 1

I confirm I have read and understood the Participant Information for the above trial. I confirm that I have received sufficient explanation of the BOOSTB4 Clinical Trial and have had the opportunity to consider the information and ask questions which have been answered satisfactorily. I have been given the Participant Information to keep.

I understand that my participation is voluntary and that I am free to withdraw my consent at any time without giving any reason, without my medical care or legal rights being affected.

I give consent for members of the BOOSTB4 Clinical Trial Team to review my medical records and use the data contained within, including access to all prenatal diagnostic tests. I consent to that information about me is processed as described in the participant information.

I give consent for members of the BOOSTB4 Clinical Trial Team to perform a pre investigation (screening) for the above mentioned trial, including investigations such as ultrasound, as it is described in the participant information.

I agree that biological samples collected from me and my child are a gift, and can be used in the research on stem cell infusion and OI as described in the Participant Information.

I agree that all samples can be stored coded in the Stockholm Medical Biobank (reg. no. 914).

I agree that all coded samples can be transferred to other hospitals or laboratories in other countries for specialist tests.

For peer review only - http://bmjopen.bmj.com/site/about/guidelines.xhtml

Consent part 1

Patient Trial Identification Number: ______________________________ (filled in by the trial)

Date of Birth (dd/mm/yyyy) / social security number pregnant woman:

____

Name of patient (pregnant woman)	Date (dd/mm/yyyy)	Signature
Name of parent (optional)	Date (dd/mm/yyyy)	Signature
Name of person taking consent	Date (dd/mm/yyyy)	Signature
Name of interpreter (if applicable)	Date (dd/mm/yyyy)	Signature

For peer review only - http://bmjopen.bmj.com/site/about/guidelines.xhtml

Karolinska Institutet | BOOSTB4 Boost Brittle Bones Before Birth | KAROLINSKA UNIVERSITETSSJUKHUSET

Consent part 2

Voluntary, mark the Yes/No boxes according to your consent.

	YES	NO
I agree that the following samples are used for the development of a non-invasive prenatal test of OI:<br>- A blood sample<br>- Leftover amniotic fluid / placenta / DNA (no new samples are taken)<br>I also agree that the biological samples can be stored coded in Stockholm's medical biobank (reg. No. 914) to be used for research related to the development of a non-invasive prenatal test, as described in the participant information, and that coded samples can sent to hospitals or laboratories in other countries for specific tests.	☐	☐
I agree that all biological samples can be stored coded at the Stockholm Medical Biobank (reg. no. 914) for future ethically approved research.	☐	☐

Name of patient (pregnant woman)	Date (dd/mm/yyyy)	Signature
Name of parent (optional)	Date (dd/mm/yyyy)	Signature
Name of person taking consent	Date (dd/mm/yyyy)	Signature
Name of interpreter (if applicable)	Date (dd/mm/yyyy)	Signature

2 copies required: Original for Investigator Site File and copy for participant.

#3A BOOSTB4 Screening Consent Form Prenatal Trial Group [29-10-2020] v1.1 SE to the
#2 BOOSTB4 Participant Information Prenatal Trial Group [29-10-2020] v1.1 SE

For peer review only - http://bmjopen.bmj.com/site/about/guidelines.xhtml

Karolinska Institutet | BOOSTB4 Boost Brittle Bones Before Birth | KAROLINSKA UNIVERSITETSSJUKHUSET

BOOSTB4: A clinical research trial of a new intervention for brittle bone disease

<u>SCREENING</u> CONSENT FORM FOR THE PRENATAL TRIAL GROUP (FATHER)

TO BE COMPLETED BEFORE SCREENING INVESTIGATIONS TAKE PLACE

Consent part 1

I confirm I have read and understood the Participant Information for the above trial. I confirm that I have received sufficient explanation of the BOOSTB4 Clinical Trial and have had the opportunity to consider the information and ask questions which have been answered satisfactorily. I have been given the Participant Information to keep.

I understand that my participation is voluntary and that I am free to withdraw my consent at any time without giving any reason, without my medical care or legal rights being affected.

I give consent for members of the BOOSTB4 Clinical Trial Team to review my medical records and use the data contained within. I consent to that information about me is processed as described in the participant information.

I give consent to donate the following for the development of a non-invasive prenatal test: A blood sample.

I give consent to that the blood sample collected from me may be used for the described research related to the development of a non-invasive prenatal test, as described in the participant information.

I agree that all samples can be stored coded in the Stockholm Medical Biobank (reg. no. 914).

I agree that all coded samples can be transferred to other hospitals or laboratories in other countries for specialist tests.

For peer review only

For peer review only - http://bmjopen.bmj.com/site/about/guidelines.xhtml

Consent part 1

Patient Trial Identification Number: ____________________________ (filled in by the trial)

Date of Birth (dd/mm/yyyy) / social security number: ______________________________

Name of patient (father)	Date (dd/mm/yyyy)	Signature
Name of person taking consent	Date (dd/mm/yyyy)	Signature
Name of interpreter (if applicable)	Date (dd/mm/yyyy)	Signature

Consent part 2

Voluntary, mark the Yes/No boxes according to your consent.

	YES	NO
I agree that all biological samples can be stored coded at the Stockholm Medical Biobank (reg. no. 914) for future ethically approved research.	☐	☐

Name of patient (father)	Date (dd/mm/yyyy)	Signature
Name of person taking consent	Date (dd/mm/yyyy)	Signature
Name of interpreter (if applicable)	Date (dd/mm/yyyy)	Signature

2 copies required: Original for Investigator Site File and copy for participant.

For peer review only

For peer review only - http://bmjopen.bmj.com/site/about/guidelines.xhtml

BOOSTB4: A clinical research trial of a new intervention for brittle bone disease

MAIN TRIAL CONSENT FORM FOR THE PRENATAL TRIAL GROUP

TO BE COMPLETED AFTER SCREENING INVESTIGATIONS HAVE BEEN PERFORMED AND ELIGIBILITY CONFIRMED

Consent part 1

I confirm I have read and understood the Participant Information for the above trial. I confirm that I have received sufficient explanation of the BOOSTB4 clinical trial and have had the opportunity to consider the information and ask questions which have been answered satisfactorily. I have been given the Participant Information to keep.

I understand that mine and my child's participation is voluntary and that I am free to withdraw them at any time without giving any reason, without our medical care or legal rights being affected.

I understand that relevant sections of mine and my child's medical notes and data collected during the trial may be looked at by responsible individuals from national and international competent authorities, the BOOSTB4 team medical doctors and clinical trial monitors that inspect trial safety, where it is relevant to mine and my child's participation in this research. I give permission for these individuals to have access to mine and my child's records. This includes medical notes and data collected at the clinical research site, medical notes and data collected within the health care (i.e. the general/family doctor and other community services). I consent to that data from me and my child are treated as described in the Participant Information.

I agree to mine and my child's doctor and local hospital being informed of our participation in the trial. I agree that they can be contacted for medical information or obtaining up-to-date contact details.

I agree to me and my child taking part in the BOOSTB4 clinical trial including the follow-up.

I agree that my child maybe can not participate in another clinical trial before the 12-month follow-up after the last stem cell dose, and that any participation in other trials must be discussed with the trial doctor.

I agree that biological samples collected from me and my child, including the placenta, amniotic fluid and umbilical cord, can be used in the described research related to stem cell infusion or OI, as described in the Participant Information.

For peer review only - http://bmjopen.bmj.com/site/about/guidelines.xhtml

I agree that all samples can be stored coded in the Stockholm Medical Biobank (reg. no. 914).

I agree that all coded samples can be transferred to other hospitals or laboratories in other countries for specialist tests.

I understand that this study using the samples may include genetic research aimed at understanding the genes related to OI and how the environment affects them (epigenetics) but that the results of these investigations are unlikely to have any implications for me or my child personally.

I agree that all discoveries (intellectual property) are a gift to the researchers and understand that I and my child will not benefit financially if the research leads to a new treatment or medical test.

I agree that I can be contacted by telephone to collect follow-up information about myself and my child.

Consent part 1

Patient Trial Identification Number: ___________________________ (filled in by the trial)

Date of Birth (dd/mm/yyyy) / social security number: ______________________________

Name of patient (pregnant woman)	Date (dd/mm/yyyy)	Signature
Name of parent (optional)	Date (dd/mm/yyyy)	Signature
Name of person taking consent	Date (dd/mm/yyyy)	Signature
Name of interpreter (if applicable)	Date (dd/mm/yyyy)	Signature

For peer review only - http://bmjopen.bmj.com/site/about/guidelines.xhtml

Consent part 2

Voluntary, mark the Yes/No boxes according to your consent.

	YES	NO
I agree that all biological samples can be stored coded in the Stockholm Medical Biobank (reg. no. 914) for future ethically approved research.	☐	☐
I agree to give consent for photographs and video footage to be taken of trial procedures; the anonymised images may be used for educational purposes or be published.	☐	☐
I agree to give contact details for a relative / friend that the BOOSTB4 trial team can contact if they are unable to get in touch with me in the future.	☐	☐
I agree to the BOOSTB4 trial team contacting me in the future about trial related activities and events.	☐	☐
I would like to receive a summary of the trial results (it will take up to five years).	☐	☐

Name of patient (pregnant woman)	Date (dd/mm/yyyy)	Signature
Name of parent (optional)	Date (dd/mm/yyyy)	Signature
Name of person taking consent	Date (dd/mm/yyyy)	Signature
Name of interpreter (if applicable)	Date (dd/mm/yyyy)	Signature

2 copies required: Original for Investigator Site File and copy for participant.

For peer review only

For peer review only - http://bmjopen.bmj.com/site/about/guidelines.xhtml

Karolinska Institutet

BOOSTB4 Boost Brittle Bones Before Birth

KAROLINSKA UNIVERSITETSSJUKHUSET

BOOSTB4: A clinical research trial of a new intervention for brittle bone disease

POST BIRTH CONSENT CONFIRMATION FOR THE PRENATAL TRIAL GROUP

TO BE COMPLETED AFTER A BABY WHICH RECEIVED BOOSTB4 PRENATALLY HAS BEEN BORN AND ELIGIBILITY RE-CONFIRMED

Consent part 1

I confirm I have read and understood the Participant Information for the above trial. I confirm that I have received sufficient explanation of the BOOSTB4 clinical trial and have had the opportunity to consider the information and ask questions which have been answered satisfactorily. I have been given the Participant Information to keep.

I understand that my child's participation is voluntary and that I am free to withdraw them at any time without giving any reason, without their medical care or legal rights being affected.

I understand that relevant sections of my child's medical notes and data collected during the trial may be looked at by responsible individuals from national and international competent authorities, the BOOSTB4 team medical doctors and clinical trial monitors that inspect trial safety, where it is relevant to my child's taking part in this research. I give permission for these individuals to have access to my child's records. This includes medical notes and data collected at the clinical research site, medical notes and data collected within the health care (i.e. the general/family doctor and other community services). I consent to that data from my child are treated as described in the Participant Information.

I agree to my child's doctor and local hospital being informed of my child's participation in the trial. I agree that they can be contacted for medical information or obtaining up-to-date contact details.

I agree to my child taking part in the BOOSTB4 clinical trial.

I agree that my child can not participate in another clinical trial before the 12-month follow-up after the last stem cell dose, and that any participation in other trials must be discussed with the trial doctor.

I agree that biological samples collected from my child are a gift as described in the Participant Information.

I agree that all samples can be stored coded in the Stockholm Medical Biobank (reg. no. 914).

For peer review only - http://bmjopen.bmj.com/site/about/guidelines.xhtml

I agree that all coded samples can be transferred to other hospitals or laboratories in other countries for specialist tests.

I understand that this study using the samples may include genetic research aimed at understanding the genes related to OI and how the environment affects them (epigenetics) but that the results of these investigations are unlikely to have any implications for my child personally.

I agree that all discoveries (intellectual property) are a gift to the researchers and understand that my child will not benefit financially if the research leads to a new treatment or medical test.

I agree that I can be contacted by telephone to collect follow-up information about my child.

Consent part 1

Patient Trial Identification Number: ____________________________ (filled in by the trial)

Date of Birth (dd/mm/yyyy) / social security number: _______________________________

Name of parent	Date (dd/mm/yyyy)	Signature
Name of parent	Date (dd/mm/yyyy)	Signature
Name of person taking consent	Date (dd/mm/yyyy)	Signature
Name of interpreter (if applicable)	Date (dd/mm/yyyy)	Signature

For peer review only - http://bmjopen.bmj.com/site/about/guidelines.xhtml

Consent part 2

Voluntary, mark the Yes/No boxes according to your consent.

	YES	NO
I agree that all biological samples can be stored coded in the Stockholm Medical Biobank (reg. no. 914) for future ethically approved research.	☐	☐
I agree to give consent for photographs and video footage to be taken of trial procedures; the anonymised images may be used for educational purposes or be published.	☐	☐
I agree to give contact details for a relative / friend that the BOOSTB4 trial team can contact if they are unable to get in touch with me in the future.	☐	☐
I agree to the BOOSTB4 trial team contacting me in the future about trial related activities and events.	☐	☐
I would like to receive a summary of the trial results (it will take up to five years).	☐	☐

Name of parent	Date (dd/mm/yyyy)	Signature
Name of parent	Date (dd/mm/yyyy)	Signature
Name of person taking consent	Date (dd/mm/yyyy)	Signature
Name of interpreter (if applicable)	Date (dd/mm/yyyy)	Signature

For peer review only - http://bmjopen.bmj.com/site/about/guidelines.xhtml

SPIRIT

STANDARD PROTOCOL ITEMS: RECOMMENDATIONS FOR INTERVENTIONAL TRIALS

SPIRIT 2013 Checklist: Recommended items to address in a clinical trial protocol and related documents*

Section/item	Item No	Description	Addressed on page number
**Administrative information**			
Title	1	Descriptive title identifying the study design, population, interventions, and, if applicable, trial acronym	1
Trial registration	2a	Trial identifier and registry name. If not yet registered, name of intended registry	3
	2b	All items from the World Health Organization Trial Registration Data Set	Supplementary material
Protocol version	3	Date and version identifier	Supplementary material
Funding	4	Sources and types of financial, material, and other support	18_
Roles and responsibilities	5a	Names, affiliations, and roles of protocol contributors	1
	5b	Name and contact information for the trial sponsor	1, 9
	5c	Role of study sponsor and funders, if any, in study design; collection, management, analysis, and interpretation of data; writing of the report; and the decision to submit the report for publication, including whether they will have ultimate authority over any of these activities	9, 18-19

For peer review only

	5d	Composition, roles, and responsibilities of the coordinating centre, steering committee, endpoint adjudication committee, data management team, and other individuals or groups overseeing the trial, if applicable (see Item 21a for data monitoring committee)	9
**Introduction**			
Background and rationale	6a	Description of research question and justification for undertaking the trial, including summary of relevant studies (published and unpublished) examining benefits and harms for each intervention	5-8
	6b	Explanation for choice of comparators	Not applicable
Objectives	7	Specific objectives or hypotheses	13
Trial design	8	Description of trial design including type of trial (eg, parallel group, crossover, factorial, single group), allocation ratio, and framework (eg, superiority, equivalence, noninferiority, exploratory)	9
**Methods: Participants, interventions, and outcomes**			
Study setting	9	Description of study settings (eg, community clinic, academic hospital) and list of countries where data will be collected. Reference to where list of study sites can be obtained	9
Eligibility criteria	10	Inclusion and exclusion criteria for participants. If applicable, eligibility criteria for study centres and individuals who will perform the interventions (eg, surgeons, psychotherapists)	Table 1, 23-24
Interventions	11a	Interventions for each group with sufficient detail to allow replication, including how and when they will be administered	11, Figure 1
	11b	Criteria for discontinuing or modifying allocated interventions for a given trial participant (eg, drug dose change in response to harms, participant request, or improving/worsening disease)	11, 12
	11c	Strategies to improve adherence to intervention protocols, and any procedures for monitoring adherence (eg, drug tablet return, laboratory tests)	Not applicable
	11d	Relevant concomitant care and interventions that are permitted or prohibited during the trial	10

For peer review only - http://bmjopen.bmj.com/site/about/guidelines.xhtml

Outcomes	12	Primary, secondary, and other outcomes, including the specific measurement variable (eg, systolic blood pressure), analysis metric (eg, change from baseline, final value, time to event), method of aggregation (eg, median, proportion), and time point for each outcome. Explanation of the clinical relevance of chosen efficacy and harm outcomes is strongly recommended	12
Participant timeline	13	Time schedule of enrolment, interventions (including any run-ins and washouts), assessments, and visits for participants. A schematic diagram is highly recommended (see Figure)	Figure 1, 11-12
Sample size	14	Estimated number of participants needed to achieve study objectives and how it was determined, including clinical and statistical assumptions supporting any sample size calculations	11
Recruitment	15	Strategies for achieving adequate participant enrolment to reach target sample size	11,16

Methods: Assignment of interventions (for controlled trials)

Allocation:

Sequence generation	16a	Method of generating the allocation sequence (eg, computer-generated random numbers), and list of any factors for stratification. To reduce predictability of a random sequence, details of any planned restriction (eg, blocking) should be provided in a separate document that is unavailable to those who enrol participants or assign interventions	11
Allocation concealment mechanism	16b	Mechanism of implementing the allocation sequence (eg, central telephone; sequentially numbered, opaque, sealed envelopes), describing any steps to conceal the sequence until interventions are assigned	Not applicable
Implementation	16c	Who will generate the allocation sequence, who will enrol participants, and who will assign participants to interventions	11
Blinding (masking)	17a	Who will be blinded after assignment to interventions (eg, trial participants, care providers, outcome assessors, data analysts), and how	Not applicable
	17b	If blinded, circumstances under which unblinding is permissible, and procedure for revealing a participant's allocated intervention during the trial	Not applicable

Methods: Data collection, management, and analysis

Data collection methods	18a	Plans for assessment and collection of outcome, baseline, and other trial data, including any related processes to promote data quality (eg, duplicate measurements, training of assessors) and a description of study instruments (eg, questionnaires, laboratory tests) along with their reliability and validity, if known. Reference to where data collection forms can be found, if not in the protocol	12
	18b	Plans to promote participant retention and complete follow-up, including list of any outcome data to be collected for participants who discontinue or deviate from intervention protocols	16
Data management	19	Plans for data entry, coding, security, and storage, including any related processes to promote data quality (eg, double data entry; range checks for data values). Reference to where details of data management procedures can be found, if not in the protocol	9
Statistical methods	20a	Statistical methods for analysing primary and secondary outcomes. Reference to where other details of the statistical analysis plan can be found, if not in the protocol	14
	20b	Methods for any additional analyses (eg, subgroup and adjusted analyses)	Not applicable
	20c	Definition of analysis population relating to protocol non-adherence (eg, as randomised analysis), and any statistical methods to handle missing data (eg, multiple imputation)	14
**Methods: Monitoring**			
Data monitoring	21a	Composition of data monitoring committee (DMC); summary of its role and reporting structure; statement of whether it is independent from the sponsor and competing interests; and reference to where further details about its charter can be found, if not in the protocol. Alternatively, an explanation of why a DMC is not needed	9
	21b	Description of any interim analyses and stopping guidelines, including who will have access to these interim results and make the final decision to terminate the trial	14
Harms	22	Plans for collecting, assessing, reporting, and managing solicited and spontaneously reported adverse events and other unintended effects of trial interventions or trial conduct	12
Auditing	23	Frequency and procedures for auditing trial conduct, if any, and whether the process will be independent from investigators and the sponsor	9

Ethics and dissemination

Research ethics approval	24	Plans for seeking research ethics committee/institutional review board (REC/IRB) approval	9
Protocol amendments	25	Plans for communicating important protocol modifications (eg, changes to eligibility criteria, outcomes, analyses) to relevant parties (eg, investigators, REC/IRBs, trial participants, trial registries, journals, regulators)	9, 17
Consent or assent	26a	Who will obtain informed consent or assent from potential trial participants or authorised surrogates, and how (see Item 32)	10
	26b	Additional consent provisions for collection and use of participant data and biological specimens in ancillary studies, if applicable	Supplementary material
Confidentiality	27	How personal information about potential and enrolled participants will be collected, shared, and maintained in order to protect confidentiality before, during, and after the trial	Supplementary material
Declaration of interests	28	Financial and other competing interests for principal investigators for the overall trial and each study site	19
Access to data	29	Statement of who will have access to the final trial dataset, and disclosure of contractual agreements that limit such access for investigators	19
Ancillary and post-trial care	30	Provisions, if any, for ancillary and post-trial care, and for compensation to those who suffer harm from trial participation	11
Dissemination policy	31a	Plans for investigators and sponsor to communicate trial results to participants, healthcare professionals, the public, and other relevant groups (eg, via publication, reporting in results databases, or other data sharing arrangements), including any publication restrictions	17
	31b	Authorship eligibility guidelines and any intended use of professional writers	18, Not applicable
	31c	Plans, if any, for granting public access to the full protocol, participant-level dataset, and statistical code	Not applicable

Appendices

For peer review only - http://bmjopen.bmj.com/site/about/guidelines.xhtml

Informed consent materials	32	Model consent form and other related documentation given to participants and authorised surrogates	Supplementary material, 10, 17
Biological specimens	33	Plans for collection, laboratory evaluation, and storage of biological specimens for genetic or molecular analysis in the current trial and for future use in ancillary studies, if applicable	Supplementary material

*It is strongly recommended that this checklist be read in conjunction with the SPIRIT 2013 Explanation & Elaboration for important clarification on the items. Amendments to the protocol should be tracked and dated. The SPIRIT checklist is copyrighted by the SPIRIT Group under the Creative Commons "Attribution-NonCommercial-NoDerivs 3.0 Unported" license.

For peer review only
